# Supplementary material for: Push-Pull Structures Based on 2-Aryl/thienyl Substituted Quinazolin-4(3H)-ones and 4-Cyanoquinazolines
Source: Molecules. 2022 Oct 22;27(21):7156. doi: 10.3390/molecules27217156 (PMC9658551; doi:10.3390/molecules27217156)
Supplement: Supplementary file 1 [file molecules-27-07156-s001.zip › molecules-1981248-supplementary.pdf]

Push-pull structures based on 2-aryl/thienyl substituted quinazolin-4(3*H*)-ones and 4-cyanoquinazolines

Tatyana N. Moshkina, Emiliya V. Nosova, Julia V. Permyakova, Galina N. Lipunova, Ekaterina F. Zhilina, Grigory A. Kim, Pavel A. Slepukhin, Valery N. Charushin

CONTENTS

1. NMR and mass spectra of bromophenyl intermediates. 2
2. NMR and mass spectra of target compounds **4-6, 8, 9, 11**........ 4
3. Crystallographic data of compounds **11**........ 28
4. Absorption, excitation and emission spectra of chromophores in toluene and MeCN 30
5. Time-resolved fluorescence emission measurements 35
6. Emission spectra of chromophores in solid state 55
7. Quantum-chemical calculations 60

1. NMR and mass spectra of bromophenyl intermediates.

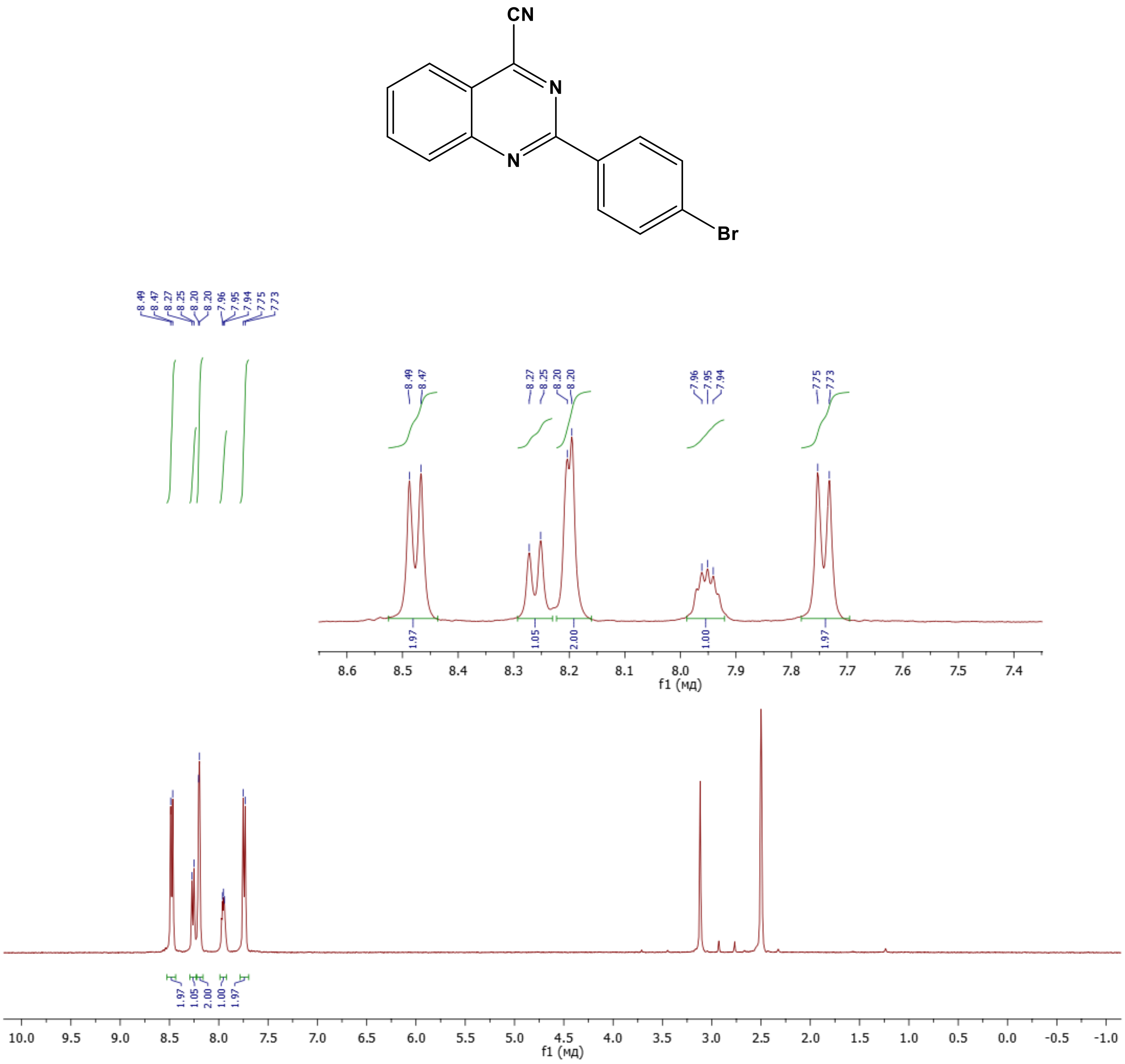

a

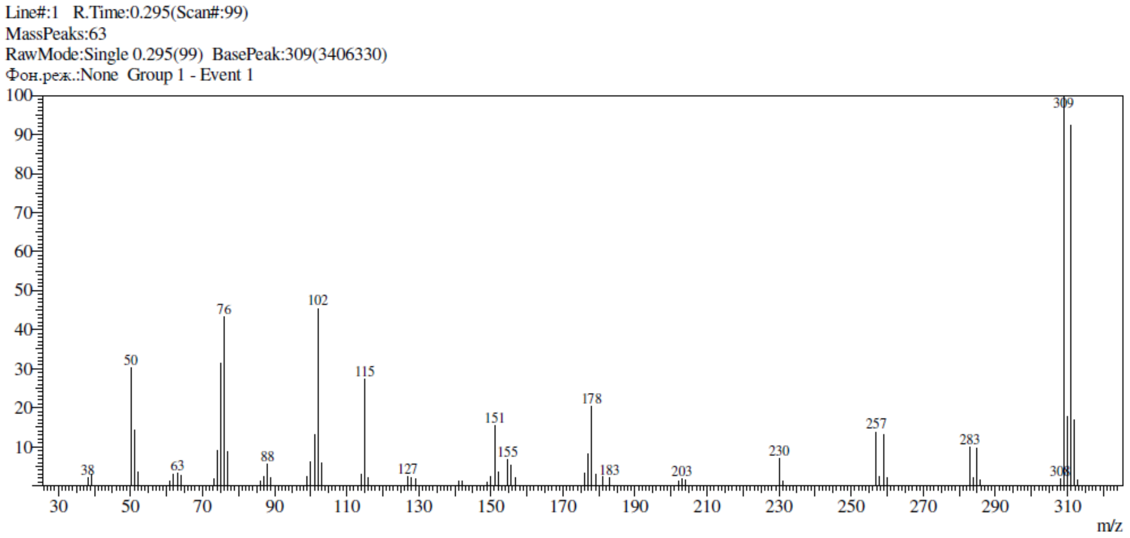

b

Figure S1. ^{1}H NMR spectrum (a) in DMSO-d$_6$ and mass spectrum (b) of 2-(4-bromophenyl)-4-cyanoquinazoline.

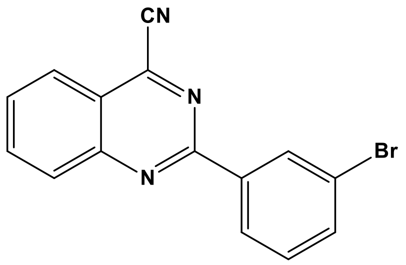

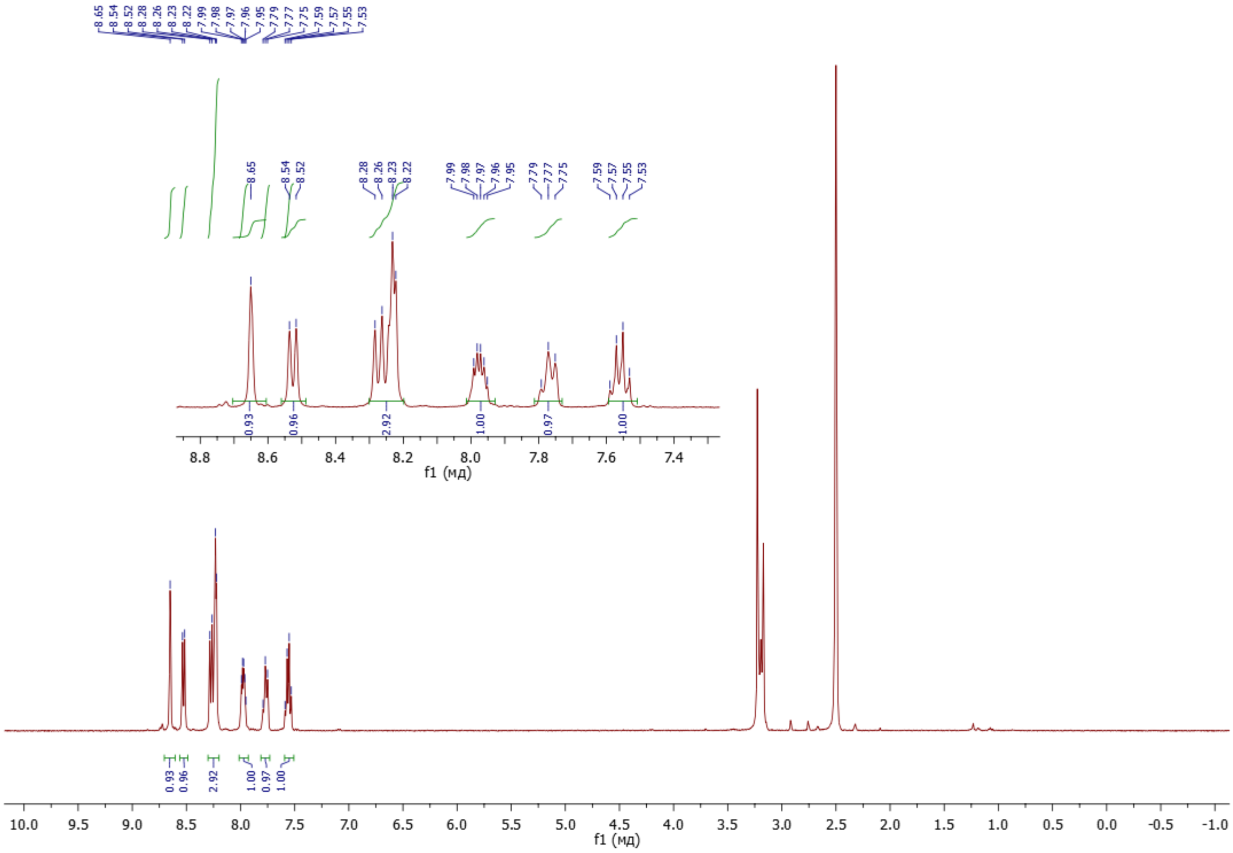

a

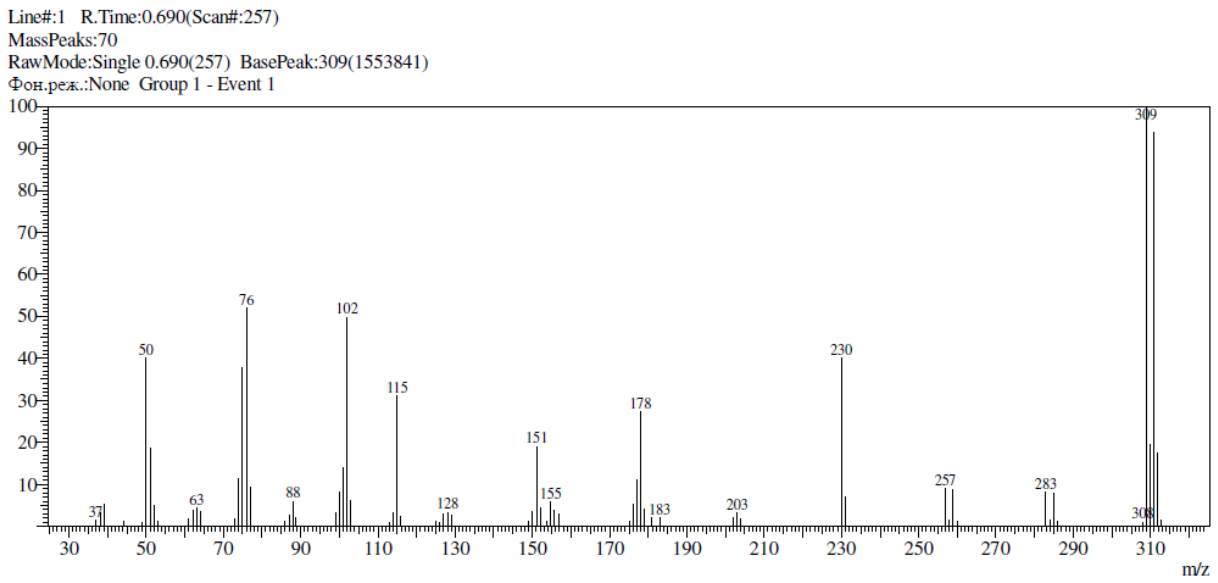

b

Figure S2. ^{1}H NMR spectrum (a) in DMSO-d_6 and mass spectrum (b) of 2-(3-bromophenyl)-4-cyanoquinazoline.

2. NMR and mass spectra of target compounds 4-6, 8, 9, 11.

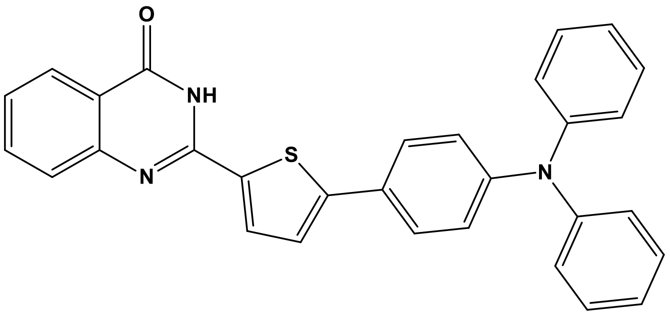

4b

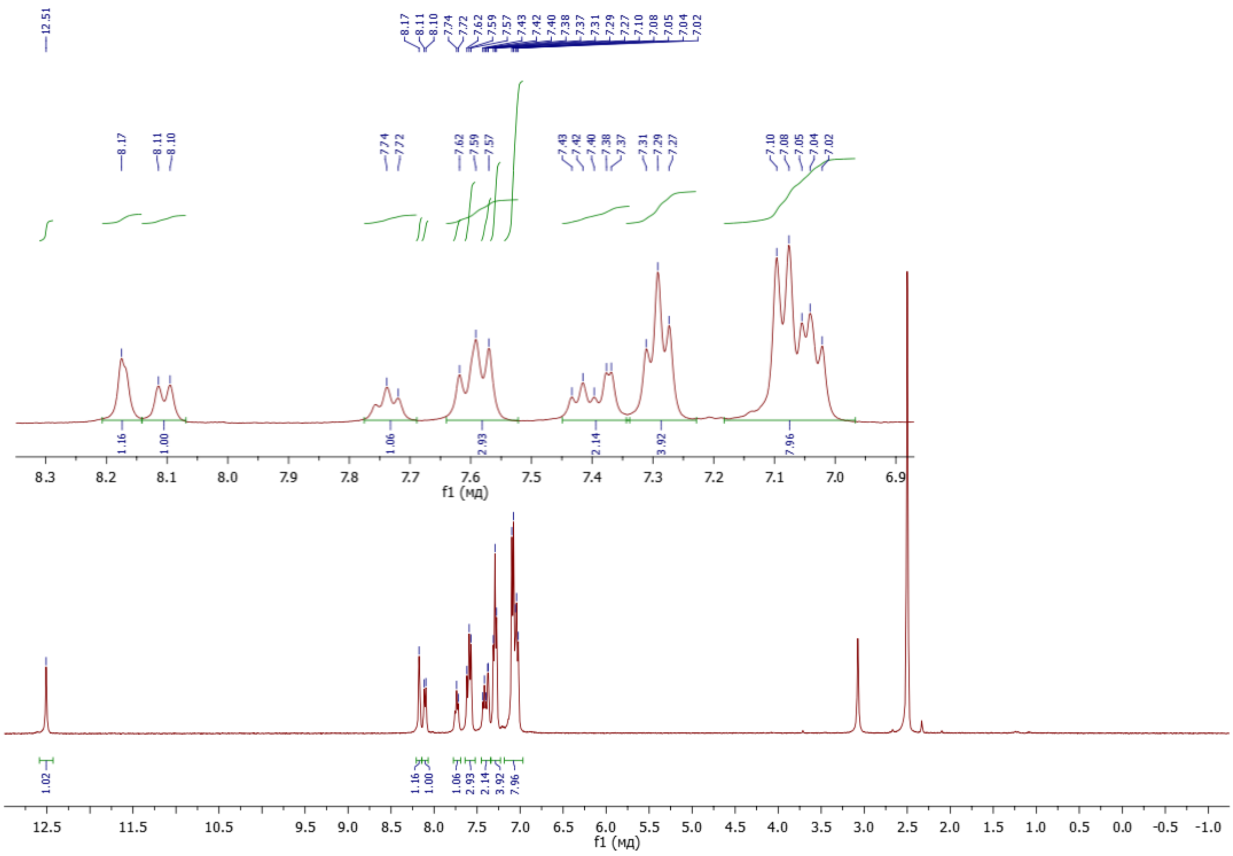

a

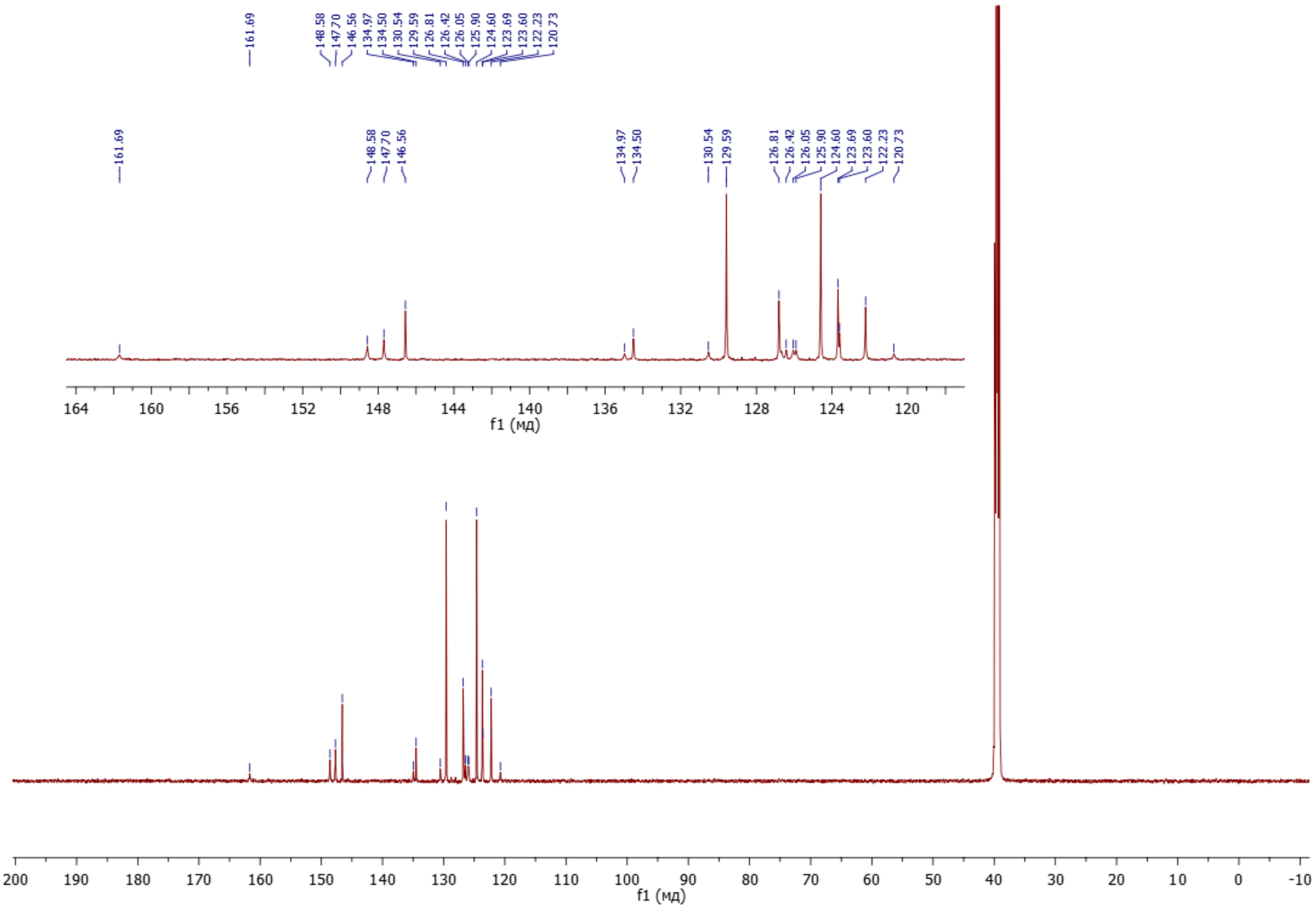

b

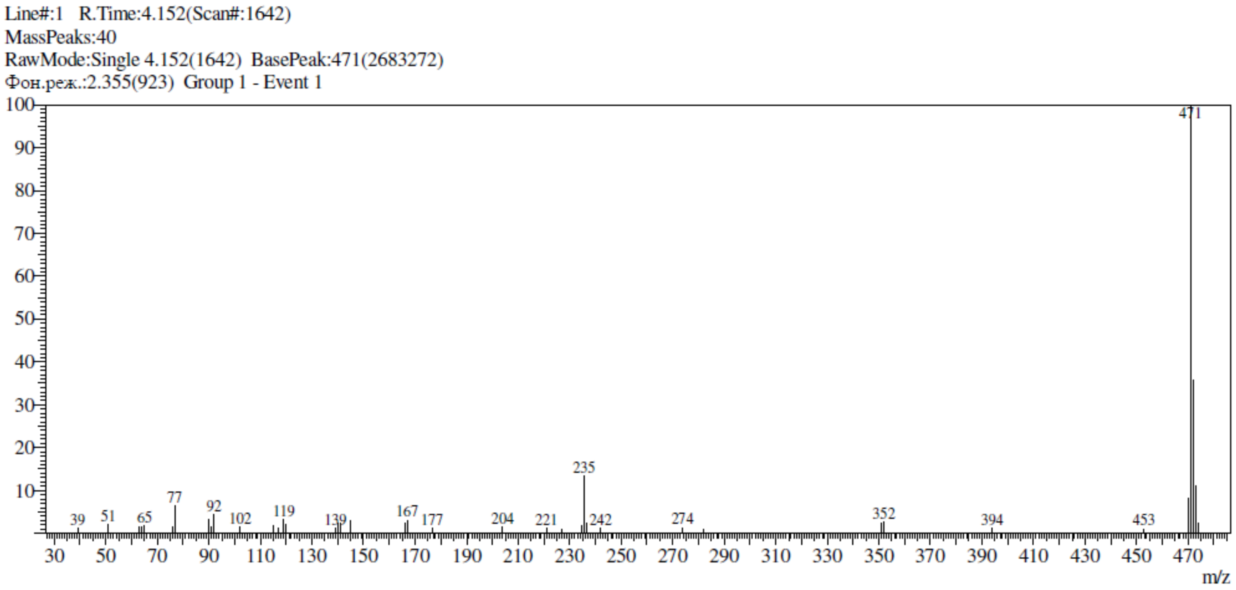

c

Figure S3. ^{1}H NMR spectrum (a) and ^{13}C NMR spectrum in DMSO-d_6 (b); mass spectrum (c) of **4b**.

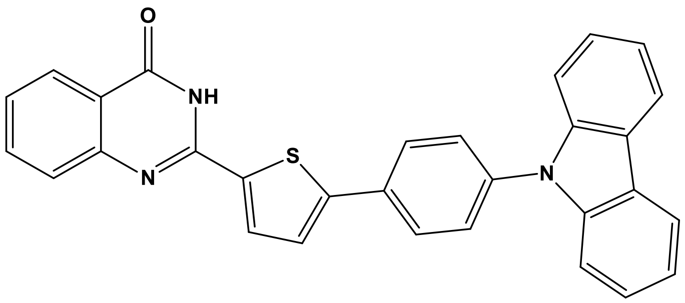

4c

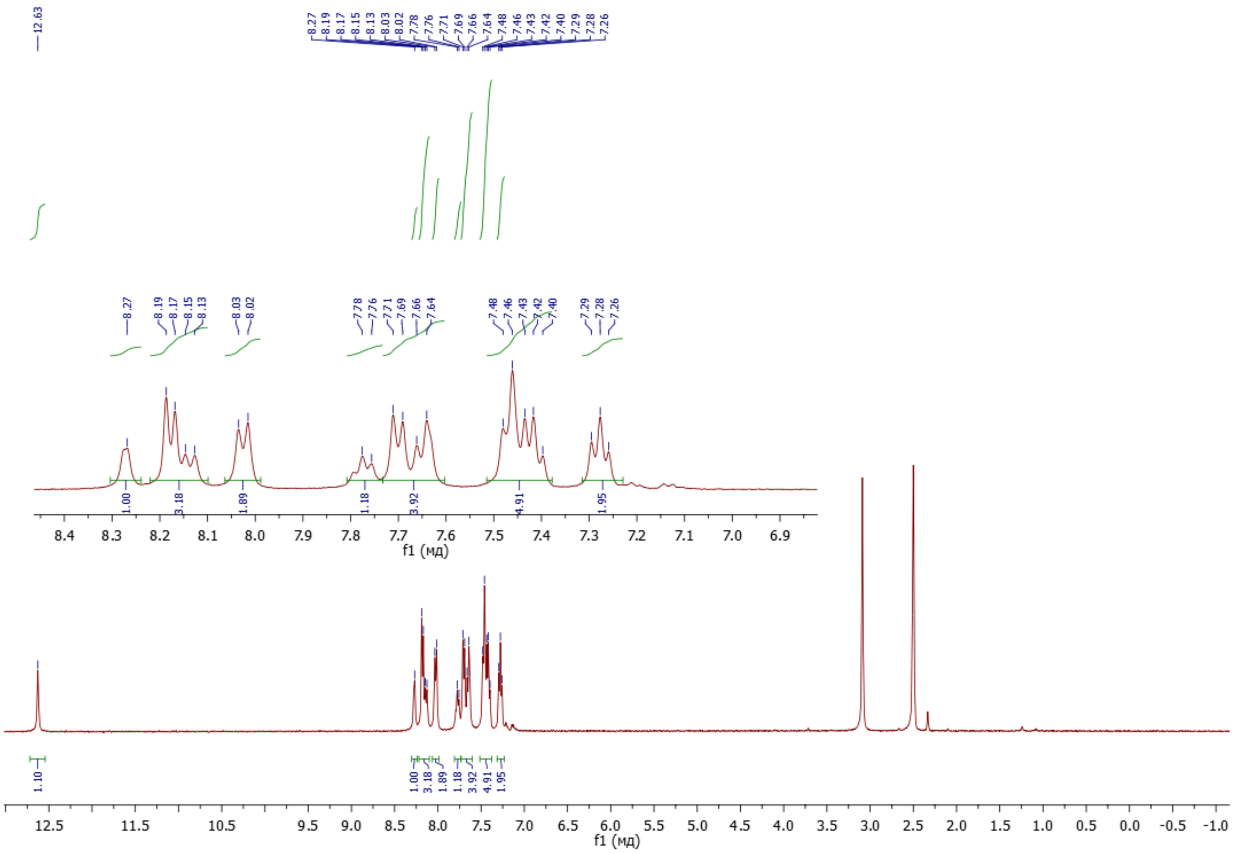

a

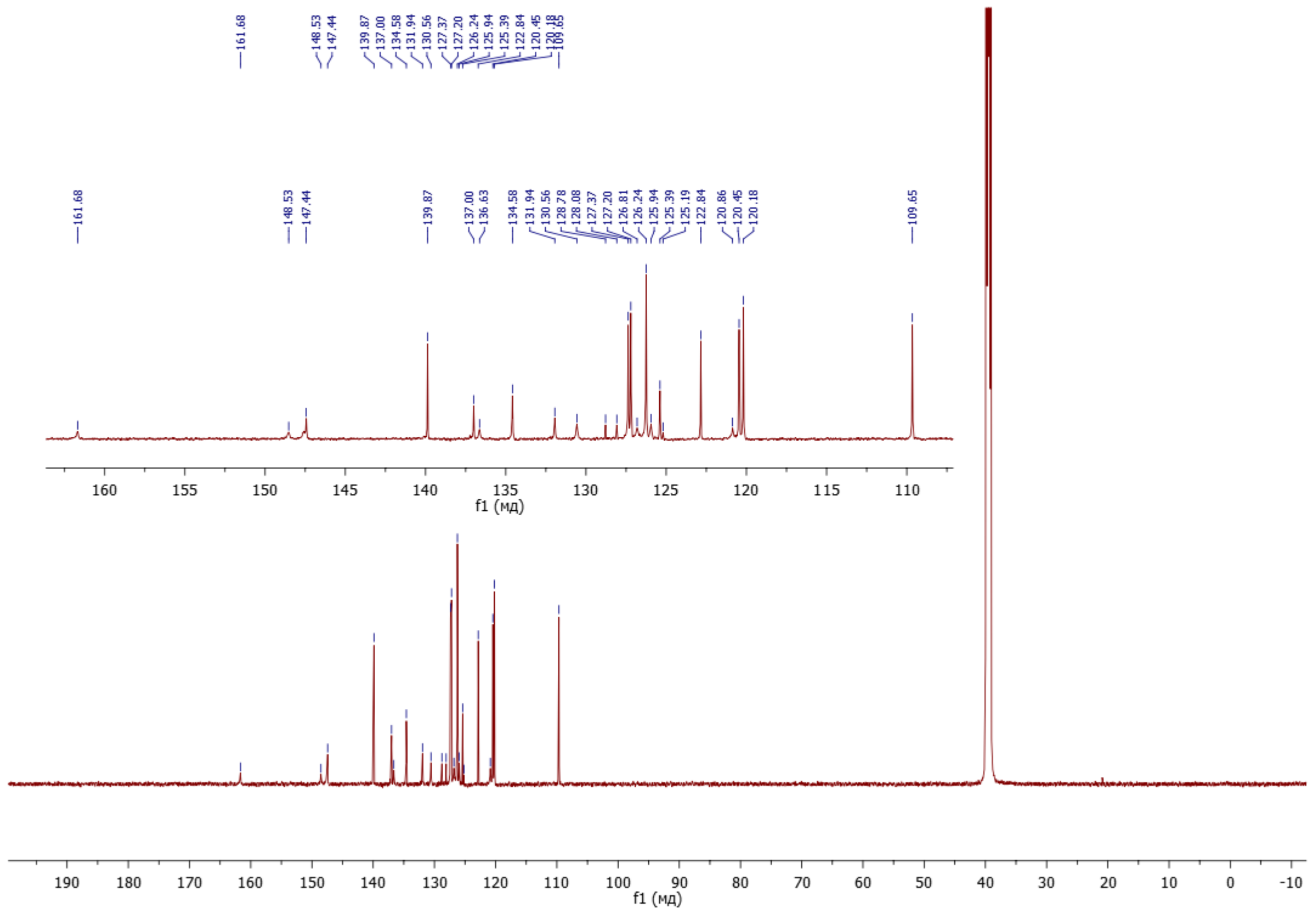

b

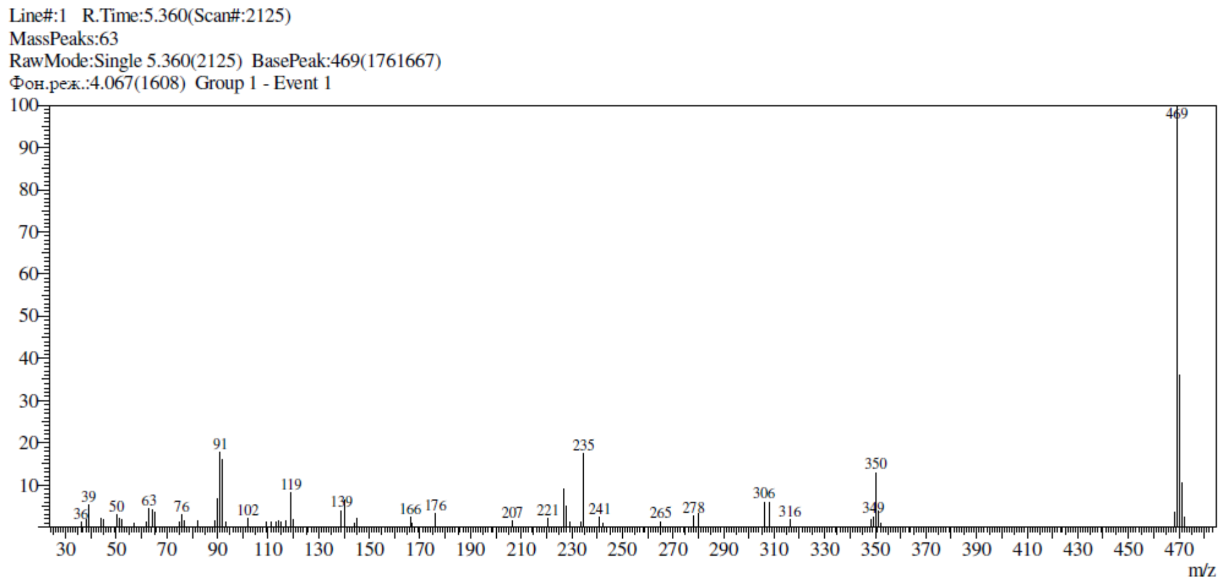

c

Figure S4. ^{1}H NMR spectrum (a) and ^{13}C NMR spectrum in DMSO-d_6 (b); mass spectrum (c) of **4c**.

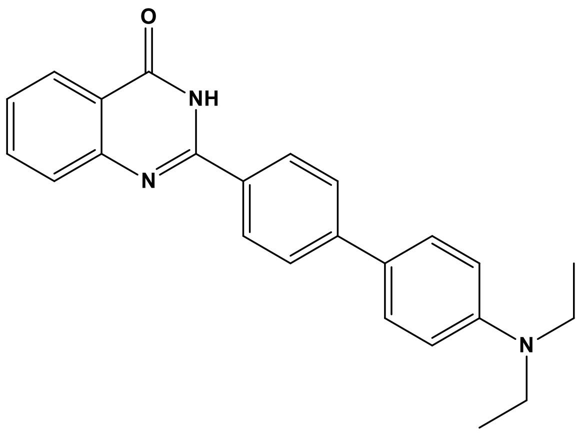
O
NH
N
N

5a

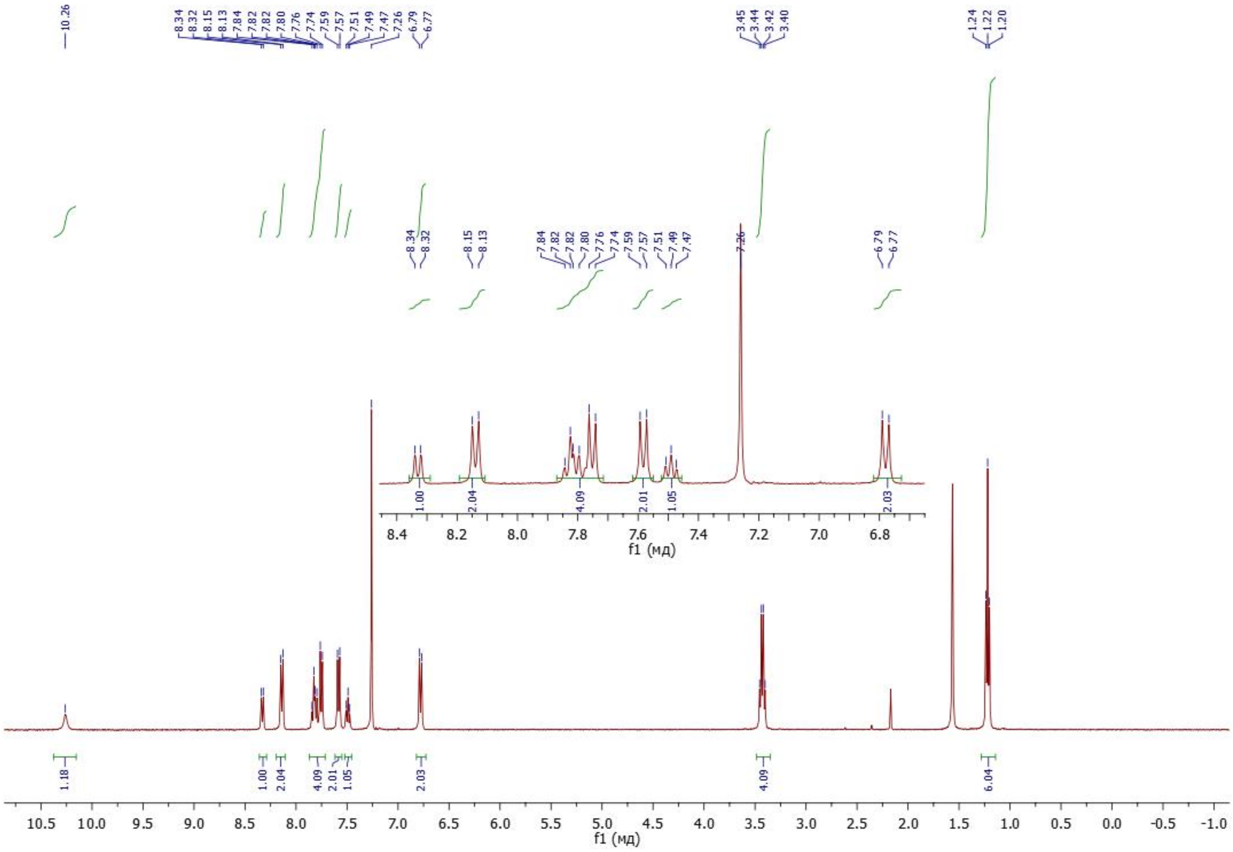
10.26
8.34
8.32
8.15
8.13
7.84
7.82
7.82
7.80
7.76
7.74
7.59
7.57
7.51
7.49
7.47
7.26
6.79
6.77
3.45
3.44
3.42
3.40
1.24
1.22
1.20
1.00
2.04
4.09
2.01
1.05
2.03
f1 (мд)
1.18
6.04

a

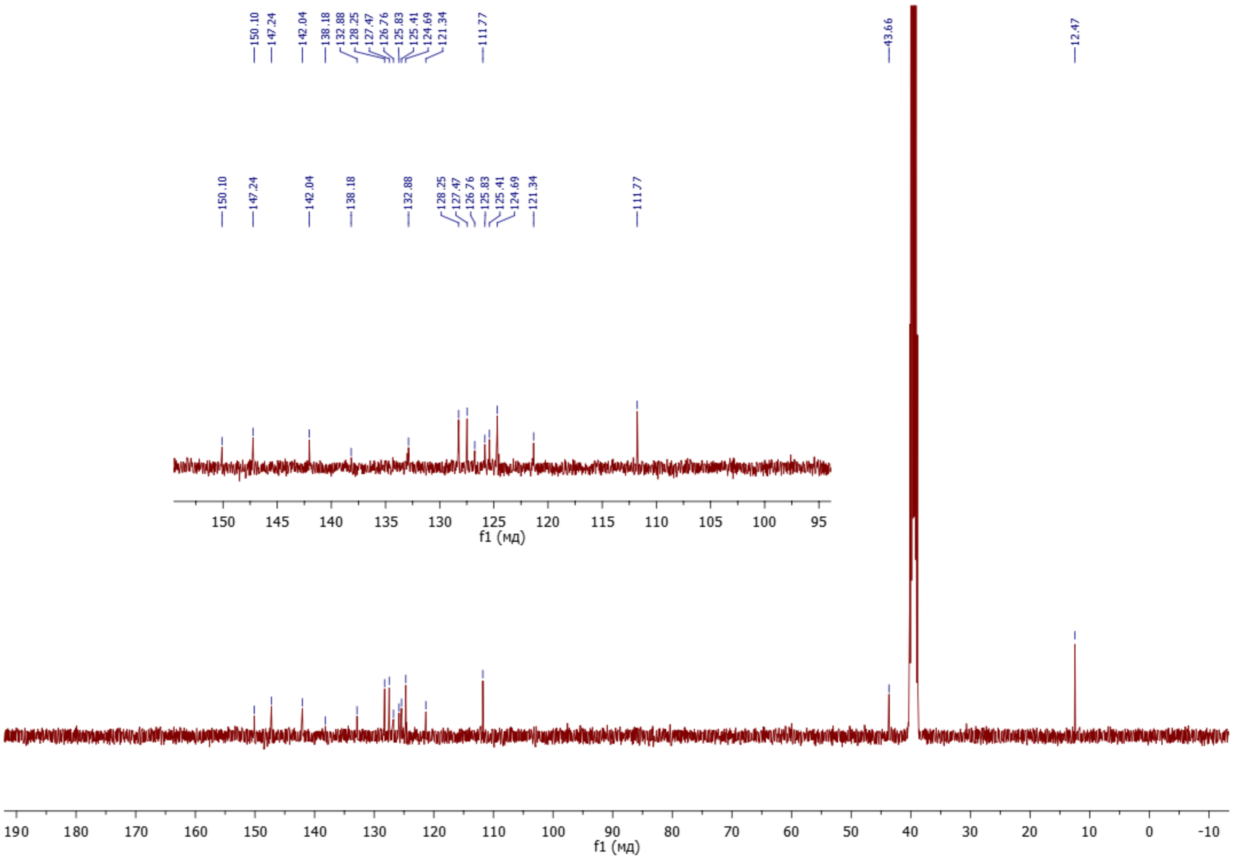

b

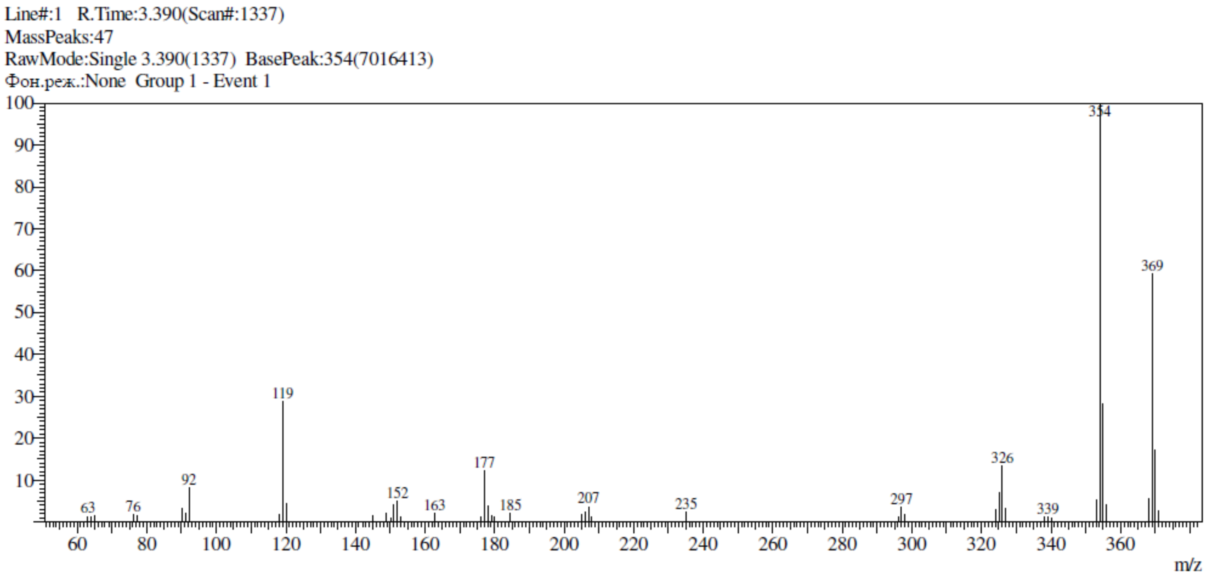

c

Figure S5. ^{1}H NMR spectrum in $DCCl_3$ (a) and ^{13}C NMR spectrum in DMSO-d_6 (b); mass spectrum (c) of **5a**.

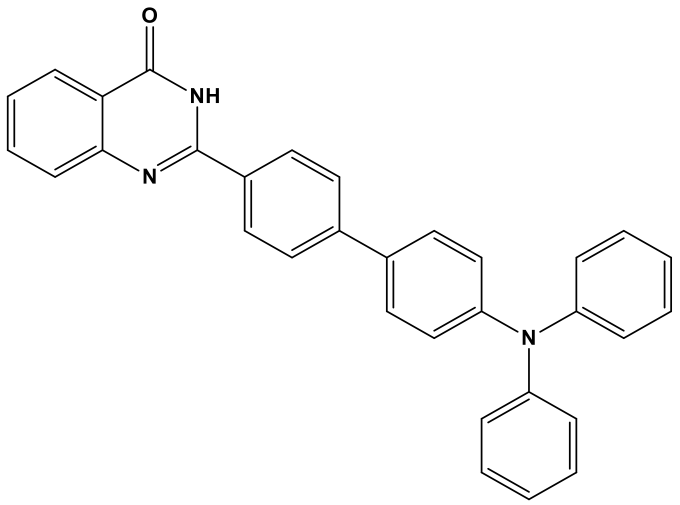

5b

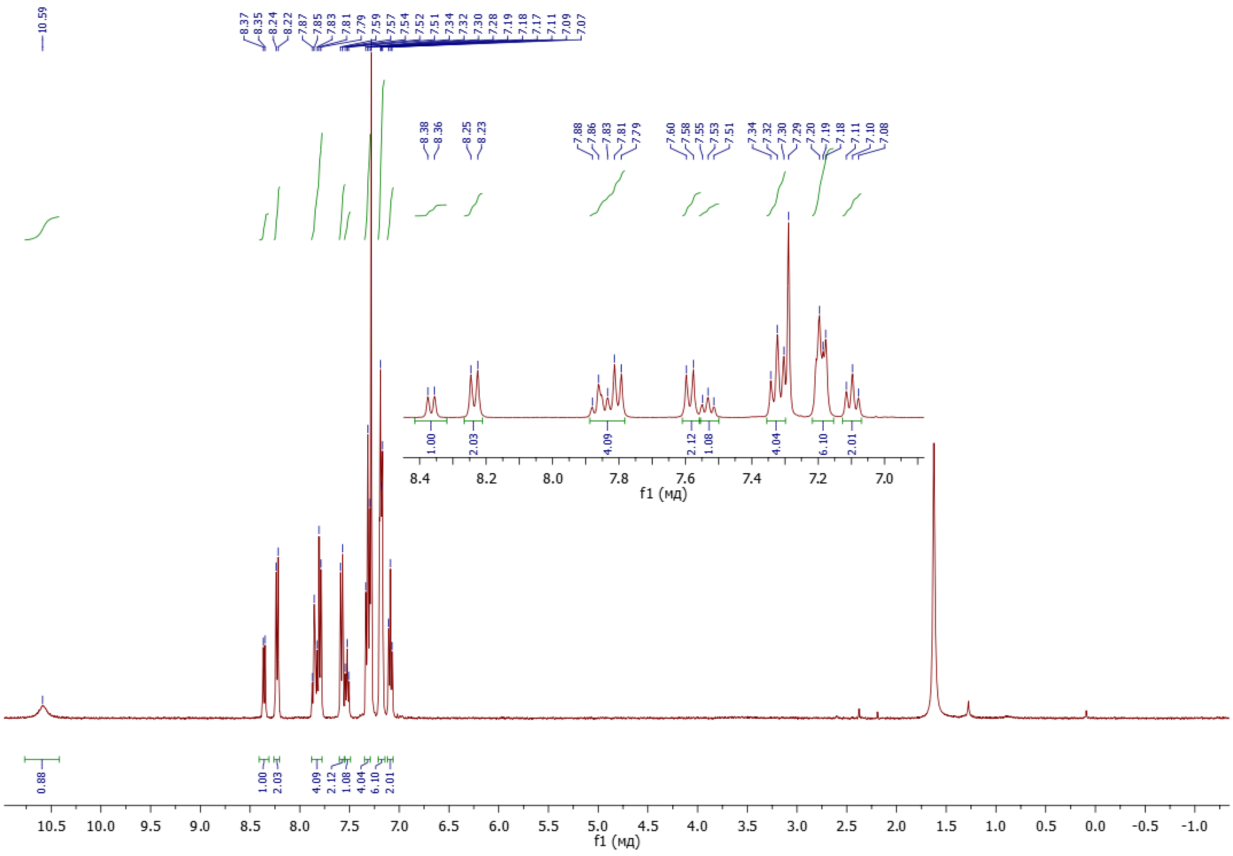

a

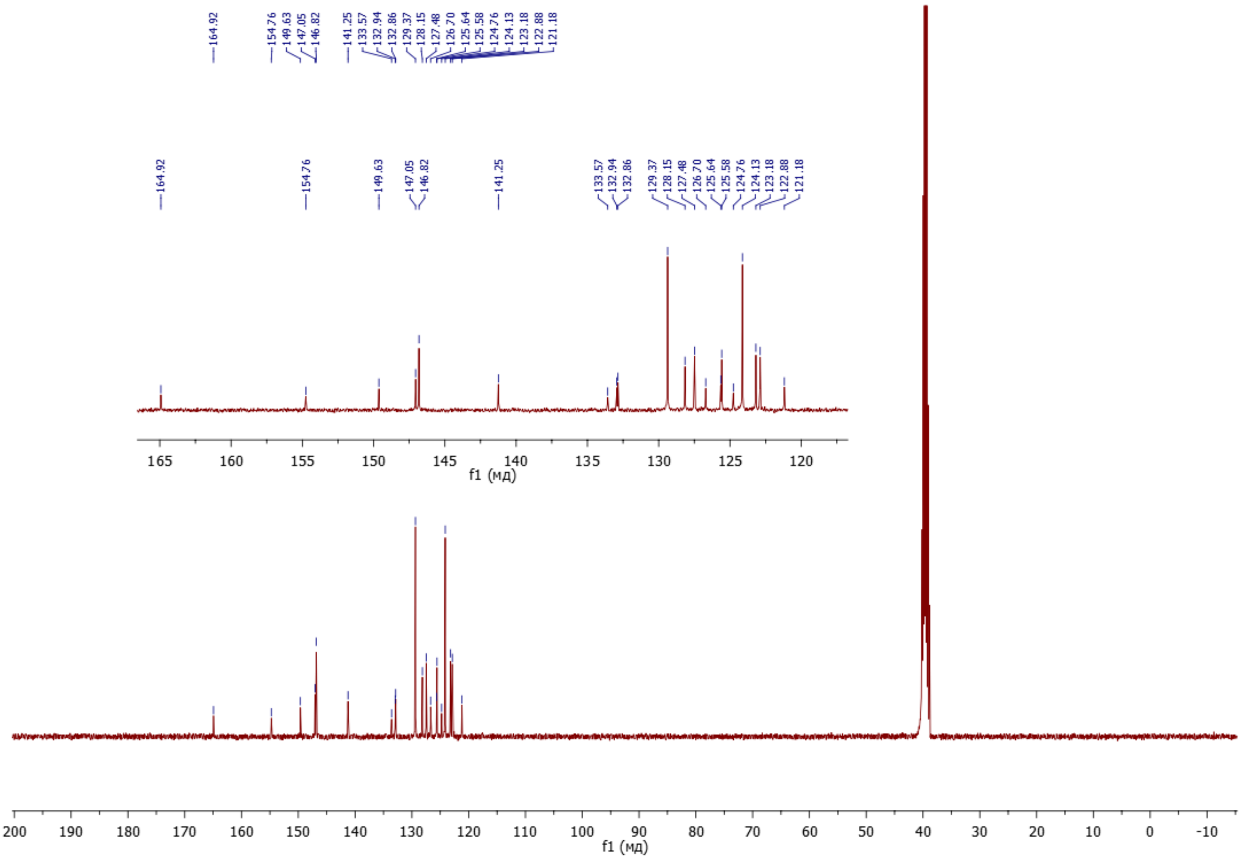

b

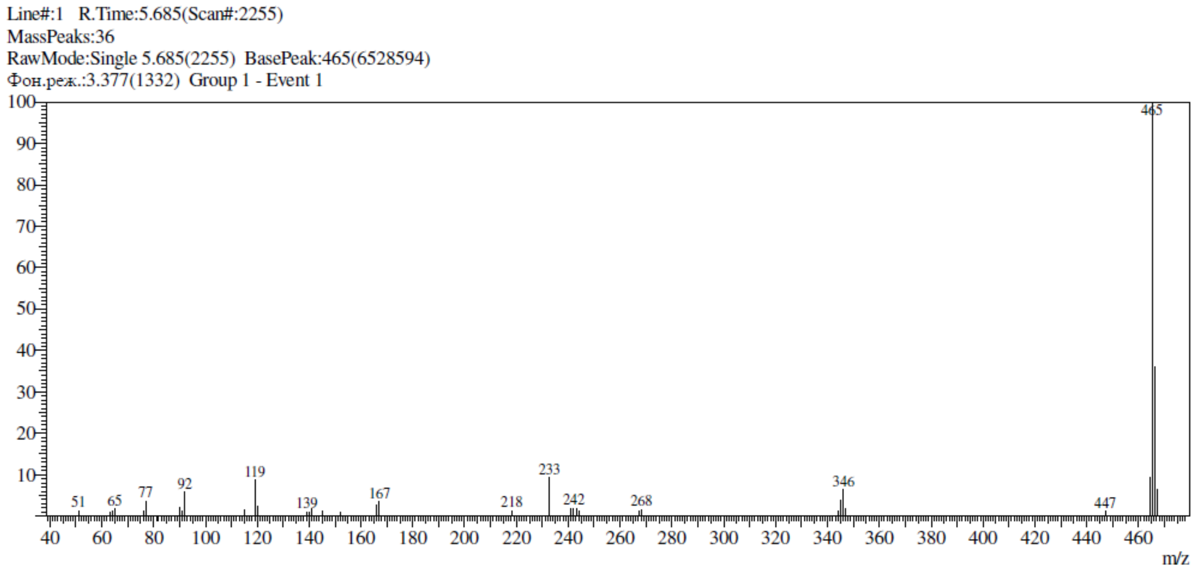

c

Figure S6. ^{1}H NMR spectrum in $DCCl_3$ (a) and ^{13}C NMR spectrum in DMSO-d_6 (b); mass spectrum (c) of **5b**.

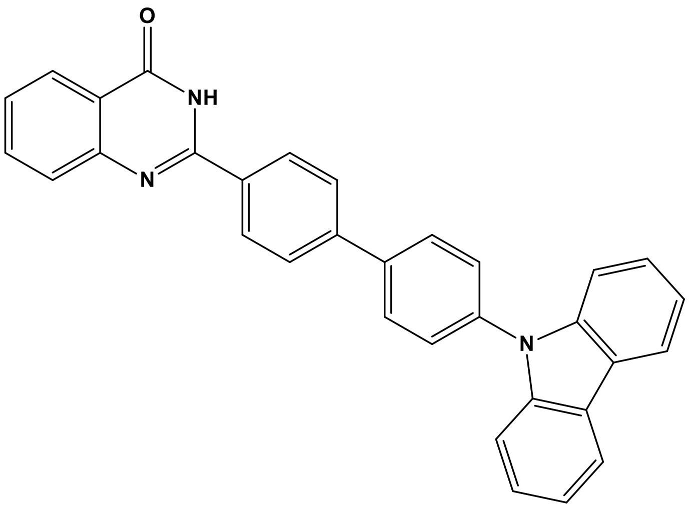

5c

a

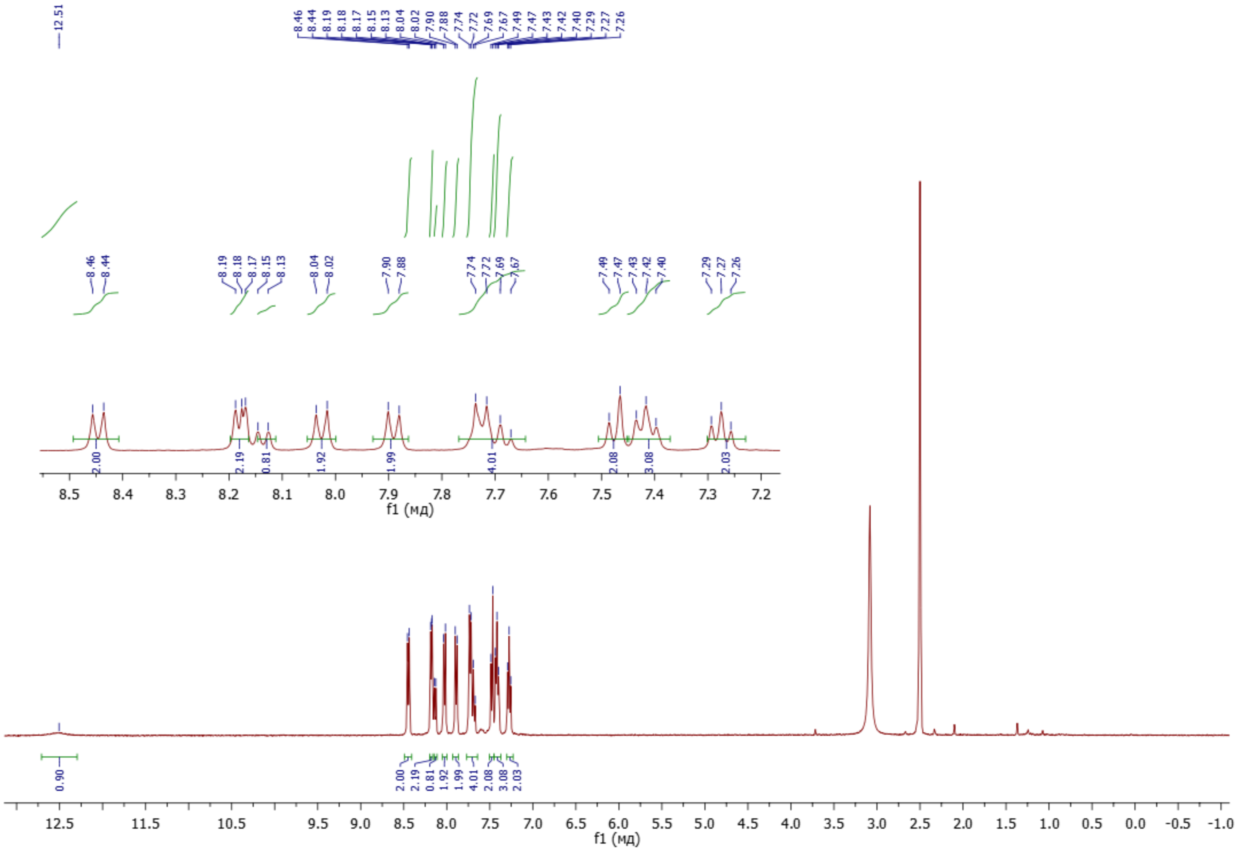

b

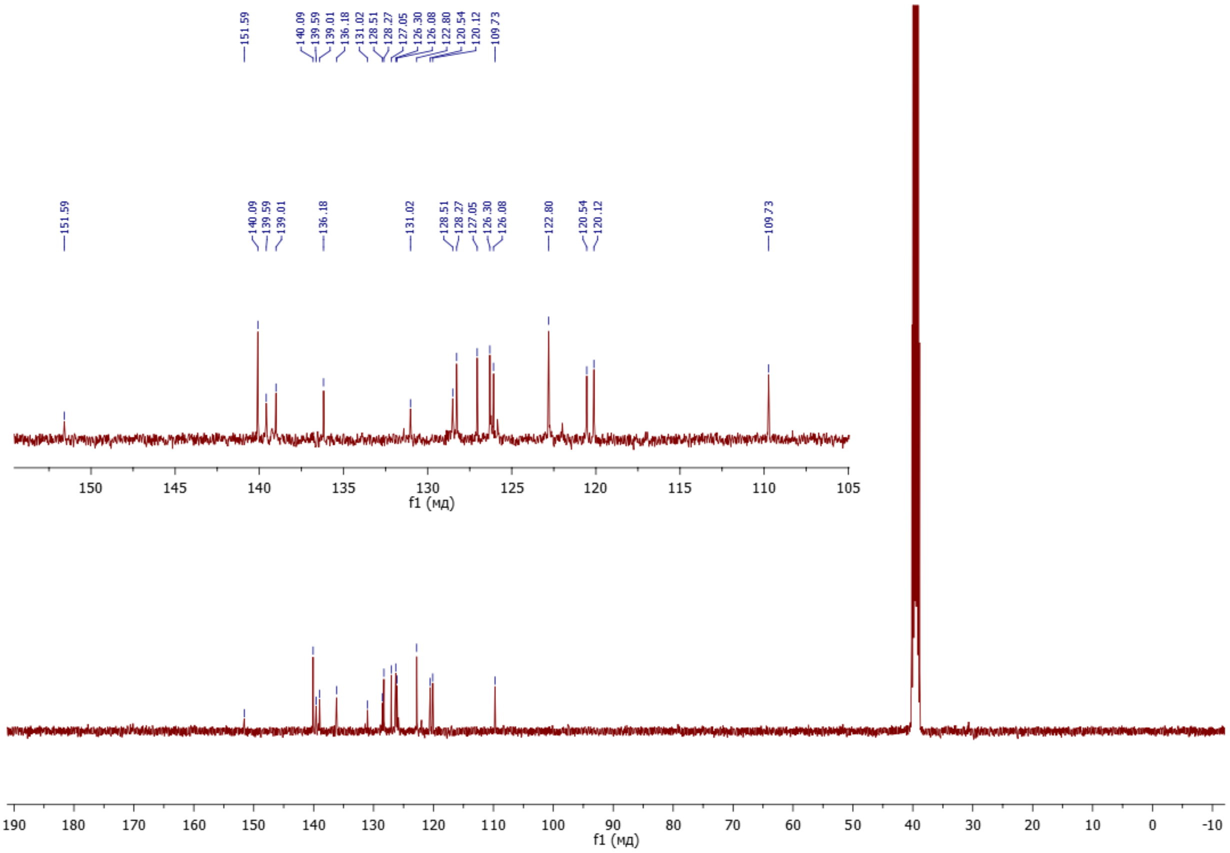

b

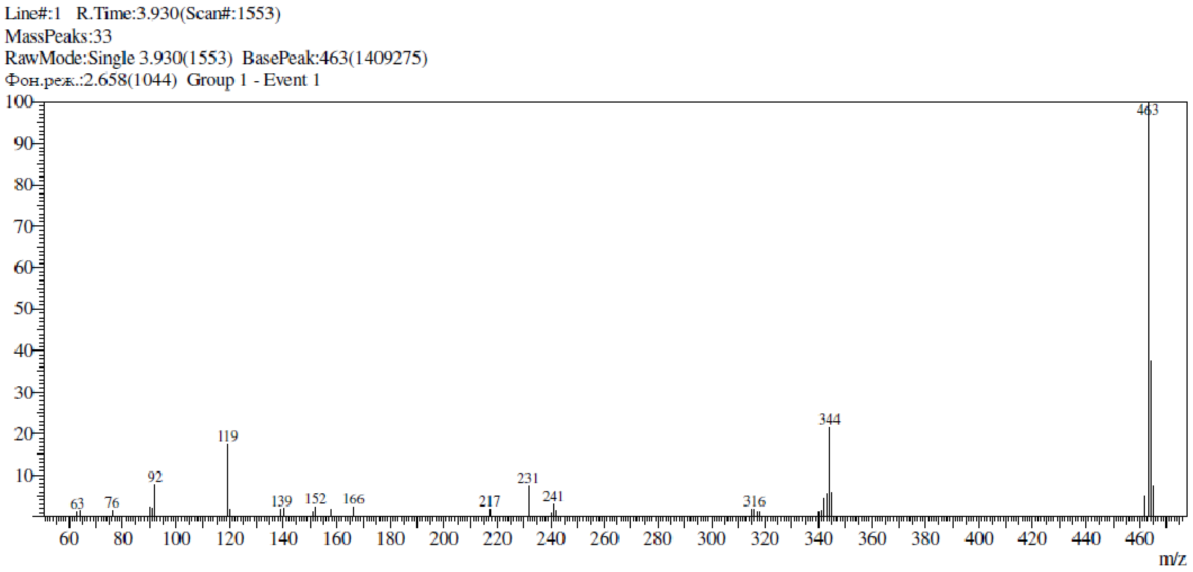

c

Figure S7. ^{1}H NMR spectrum (a) and ^{13}C NMR spectrum in DMSO-d_6 (b); mass spectrum (c) of **5c**.

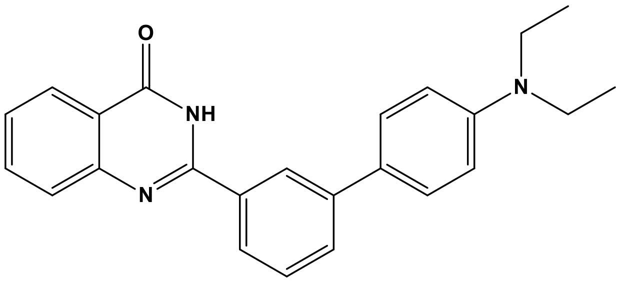
O
NH
N
N

6a

a

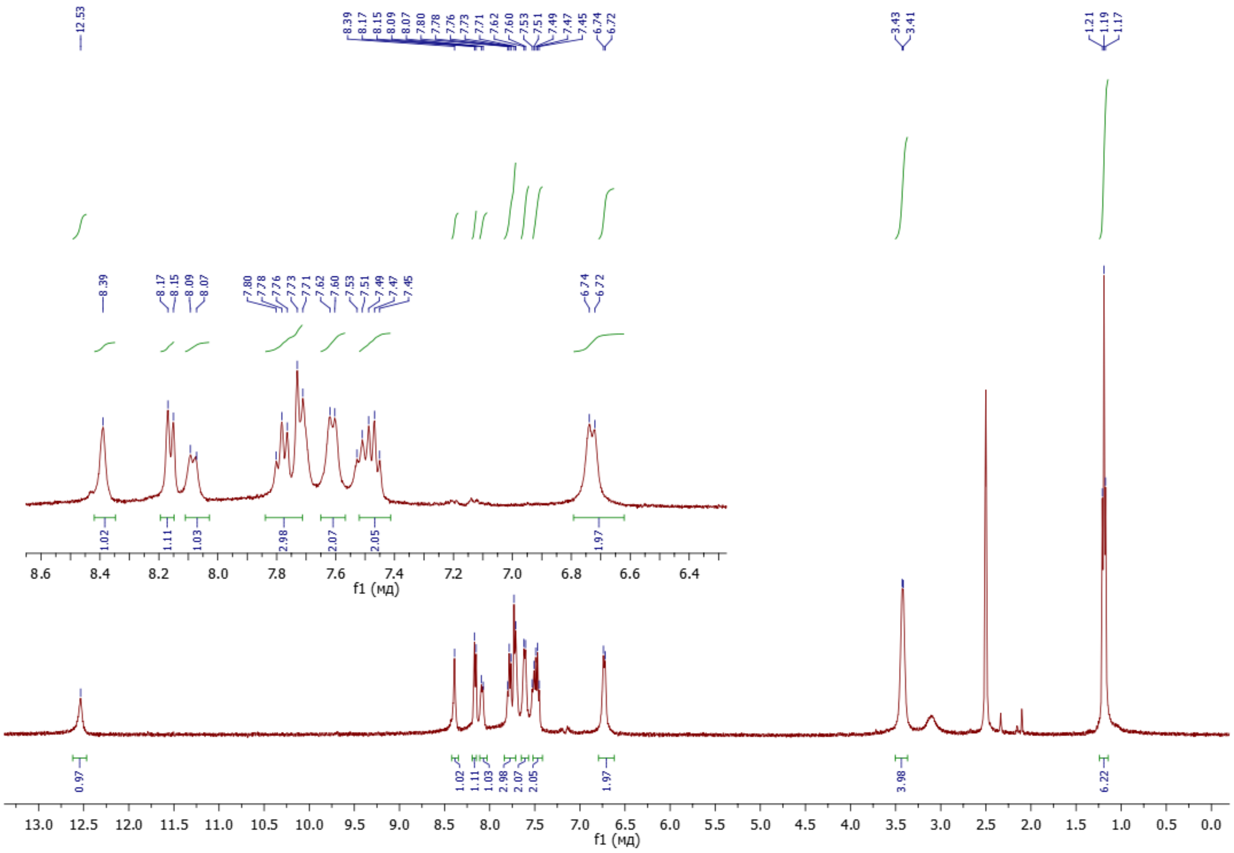
12.53
8.39 8.17 8.15 8.09 8.07 7.80 7.78 7.76 7.73 7.71 7.62 7.60 7.53 7.51 7.49 7.47 7.45 6.74 6.72
3.43 3.41
1.21 1.19 1.17
1.02 1.11 1.03 2.98 2.07 2.05 1.97
f1 (мд)
0.97 1.02 1.11 1.03 2.98 2.07 2.05 1.97 3.98 6.22
f1 (мд)

a

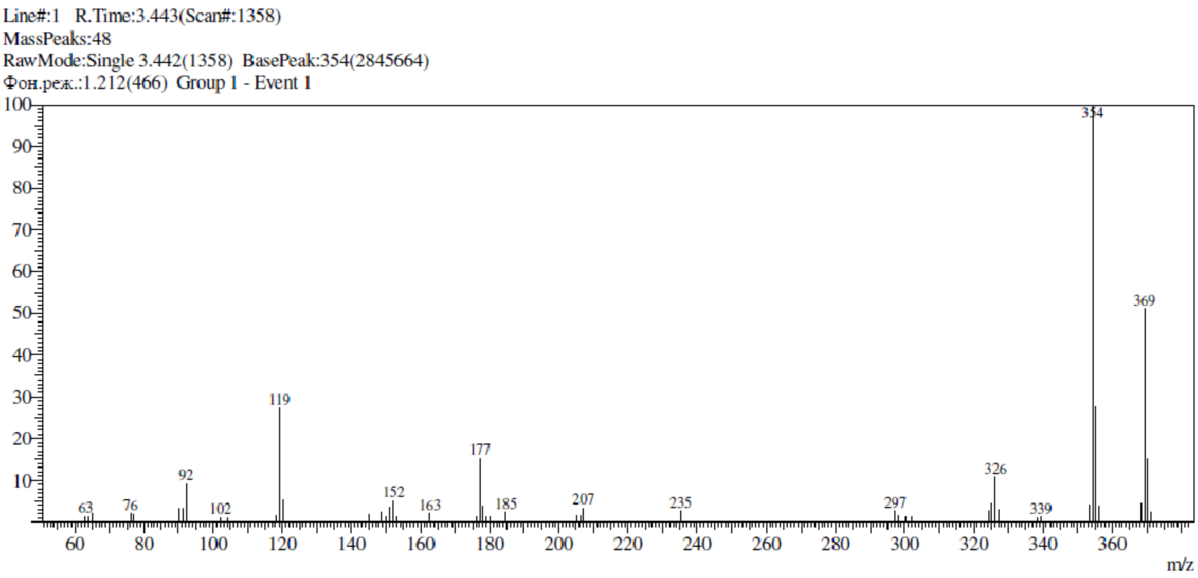

b

Figure S8. ^{1}H NMR spectrum (a) in DMSO-d$_6$; mass spectrum (b) of **6a**.

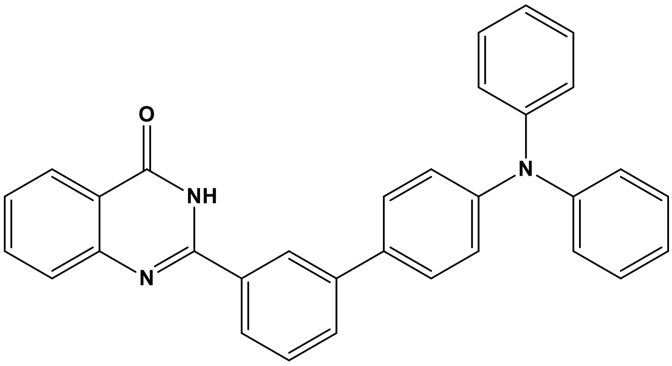

6b

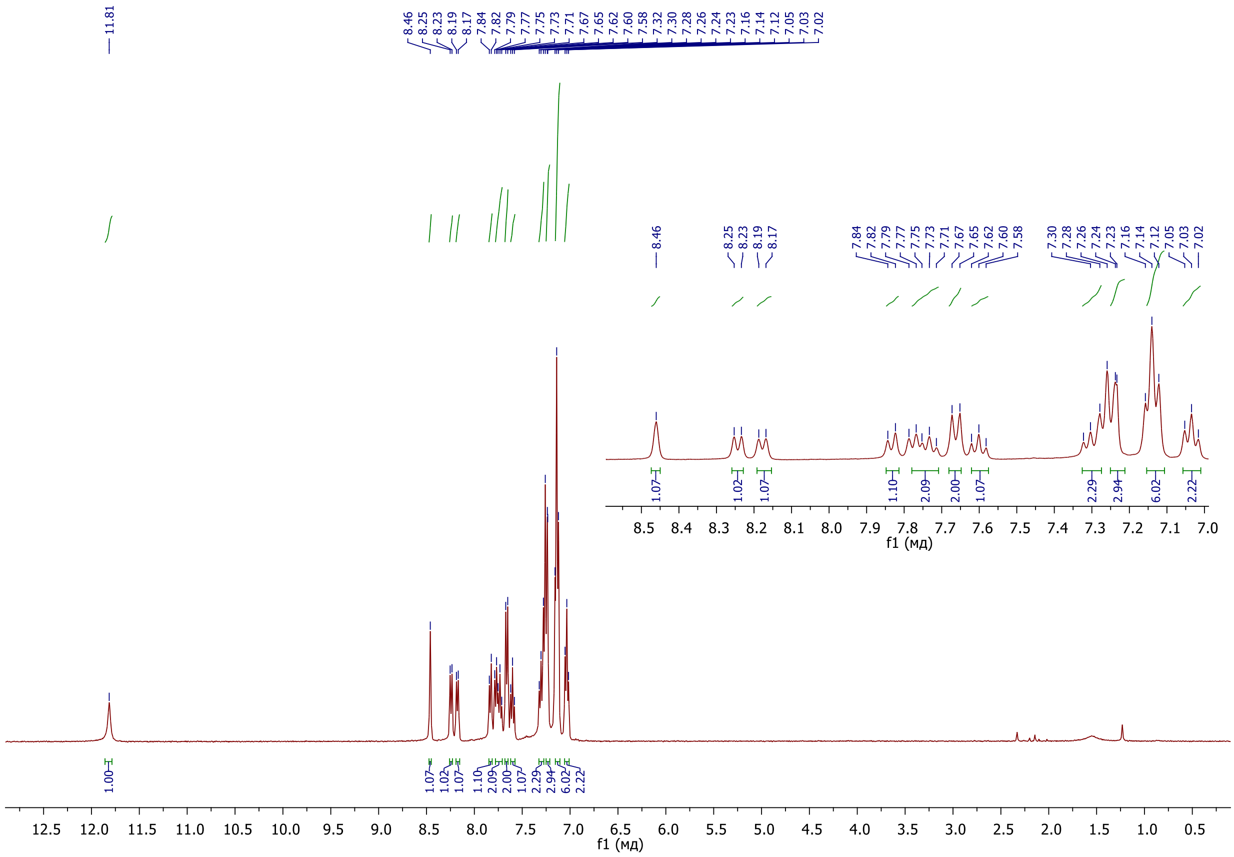
11.81
8.46 8.25 8.23 8.19 8.17 7.84 7.82 7.79 7.77 7.75 7.73 7.71 7.67 7.65 7.62 7.60 7.58 7.32 7.30 7.28 7.26 7.24 7.23 7.16 7.14 7.12 7.05 7.03 7.02
1.00 1.07 1.02 1.07 1.10 2.09 2.00 1.07 2.29 2.94 6.02 2.22
f1 (мд)

a

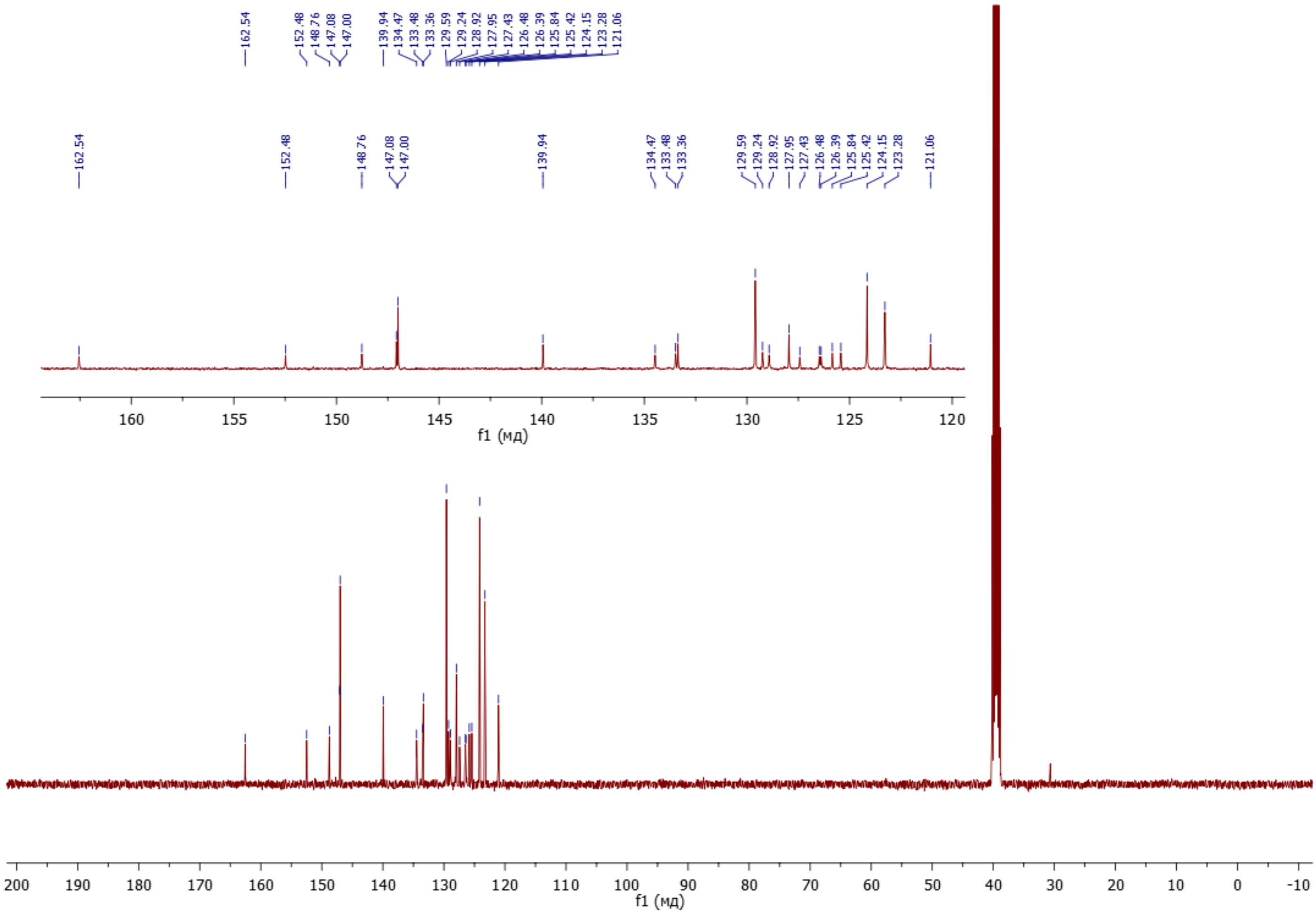
162.54 152.48 148.76 147.08 147.00 139.94 134.47 133.48 133.36 129.59 129.24 128.92 127.95 127.43 126.48 126.39 125.84 125.42 124.15 123.28 121.06
f1 (мд)

b

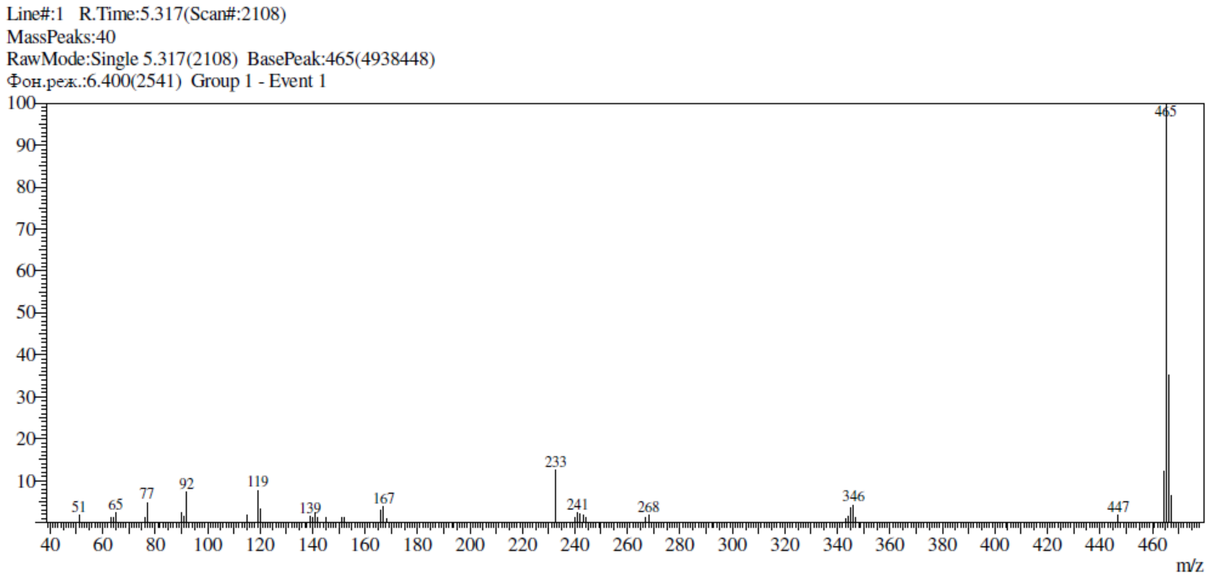

c

Figure S9. ^{1}H NMR spectrum in $DCCl_3$ (a), ^{13}C NMR spectrum in DMSO-d_6 (b) and mass spectrum (c) of **6b**.

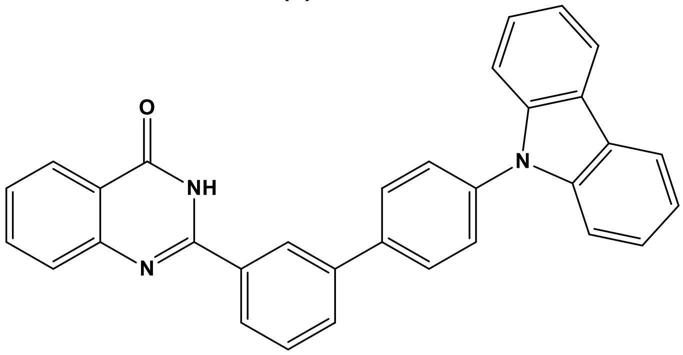

6c

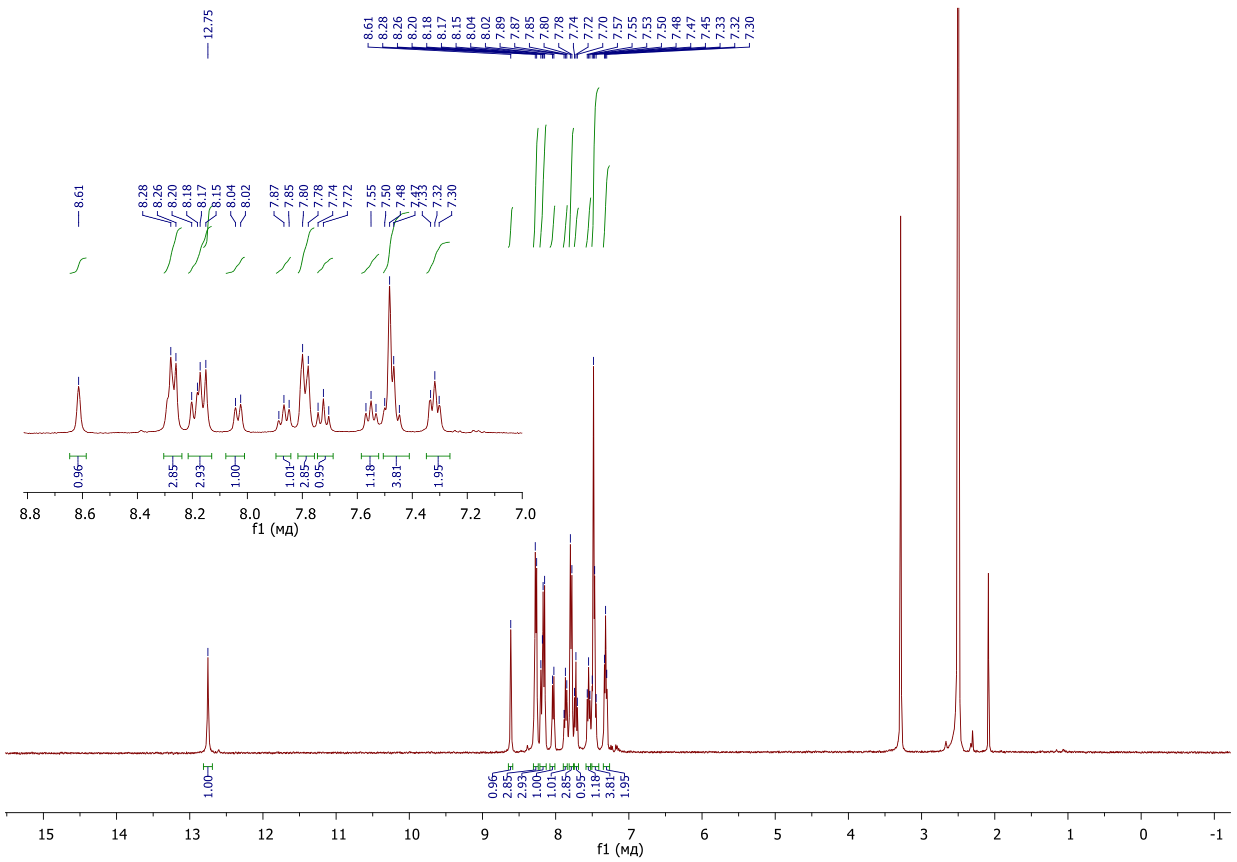

a

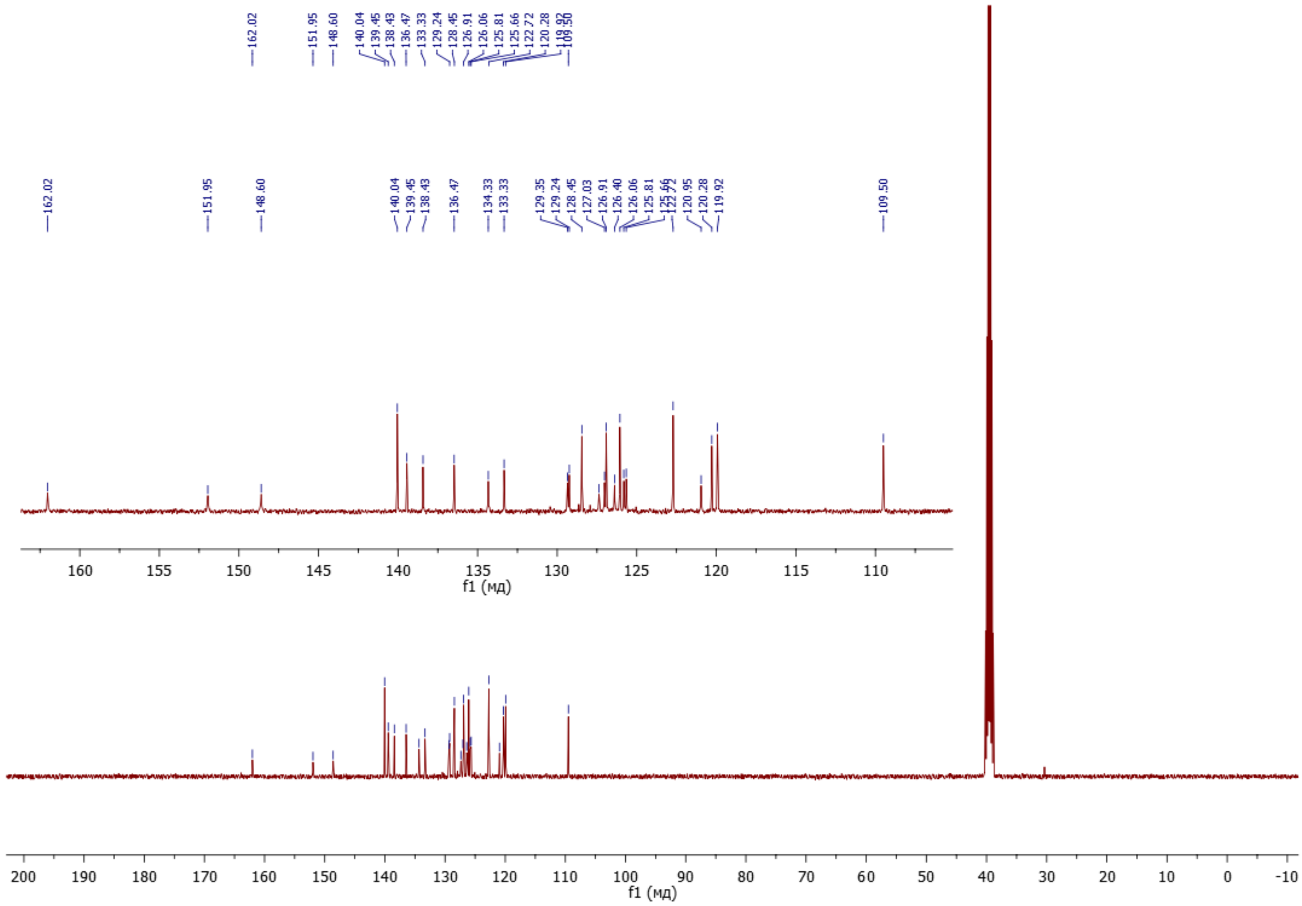

b

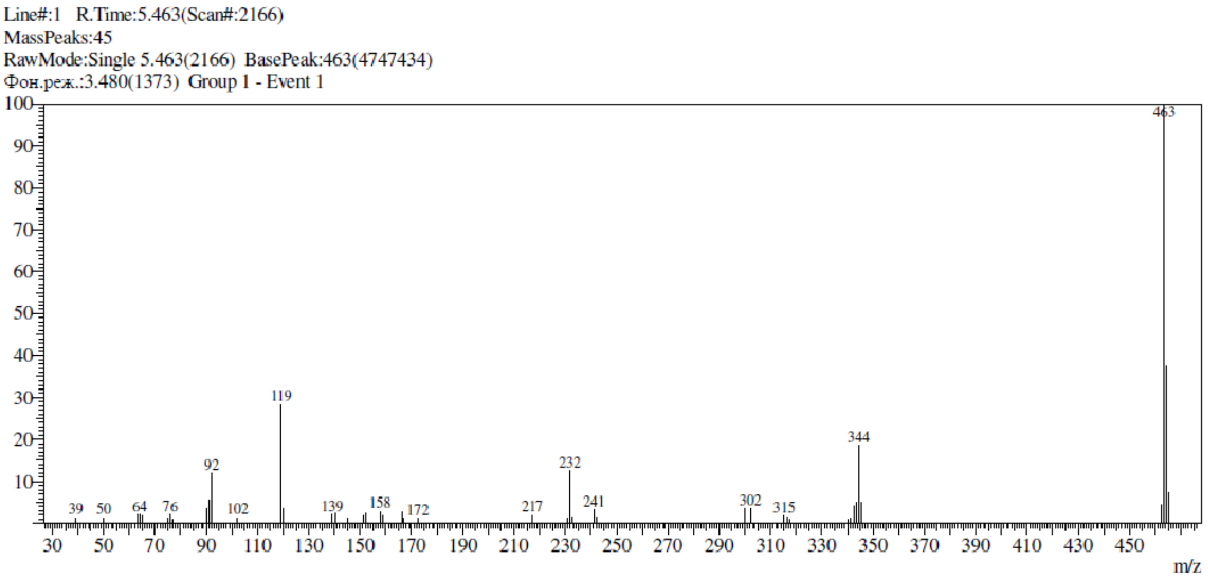

c

Figure S10. ^{1}H NMR spectrum (a) and ^{13}C NMR spectrum in DMSO-d$_6$ (b); mass spectrum (c) of **6c**.

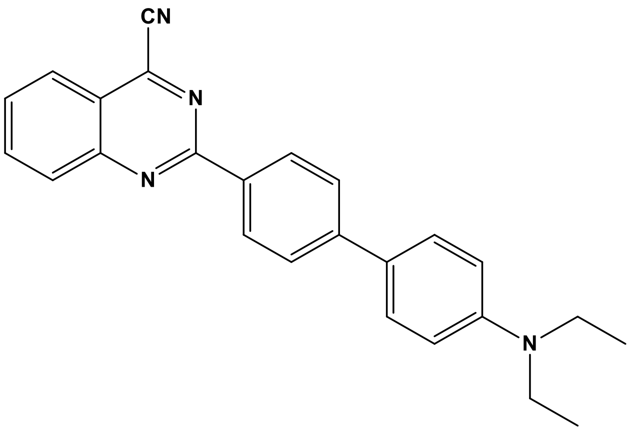

8a

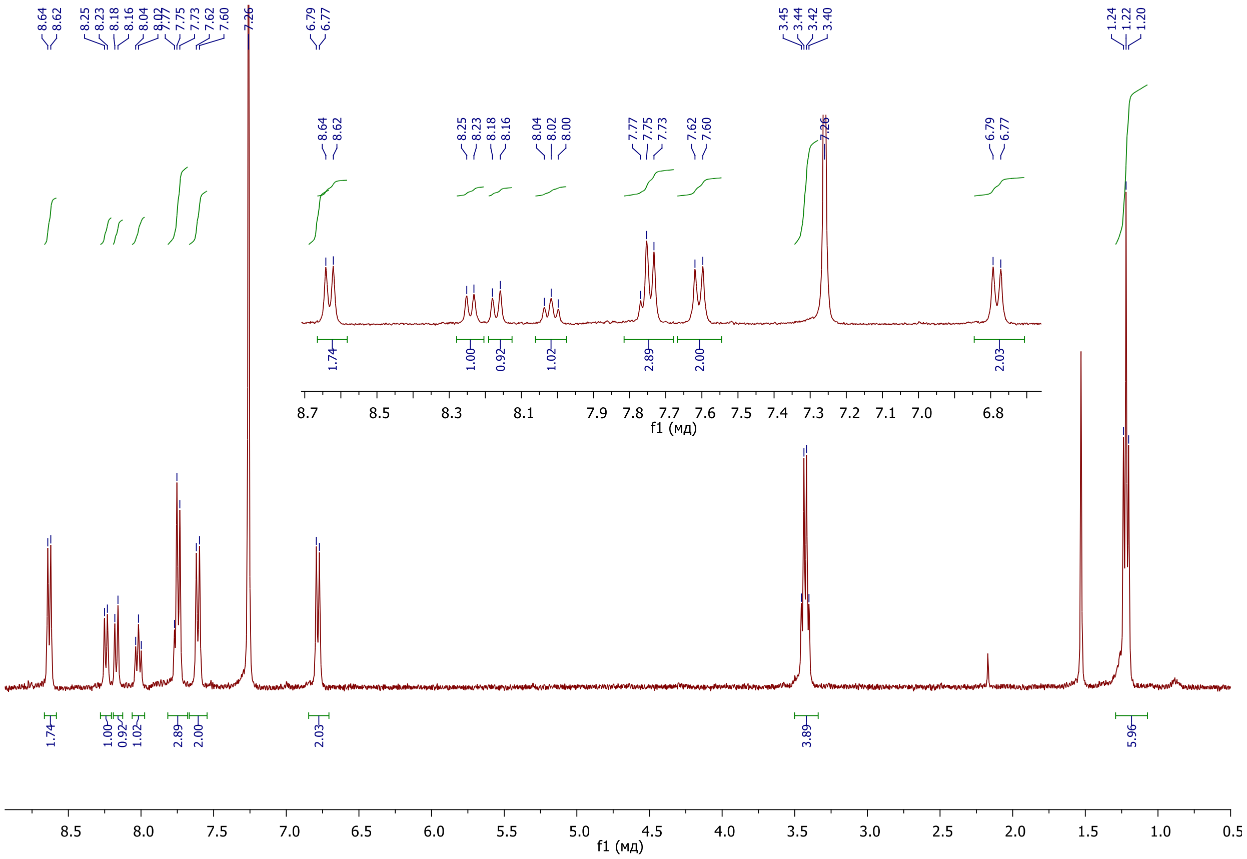

a

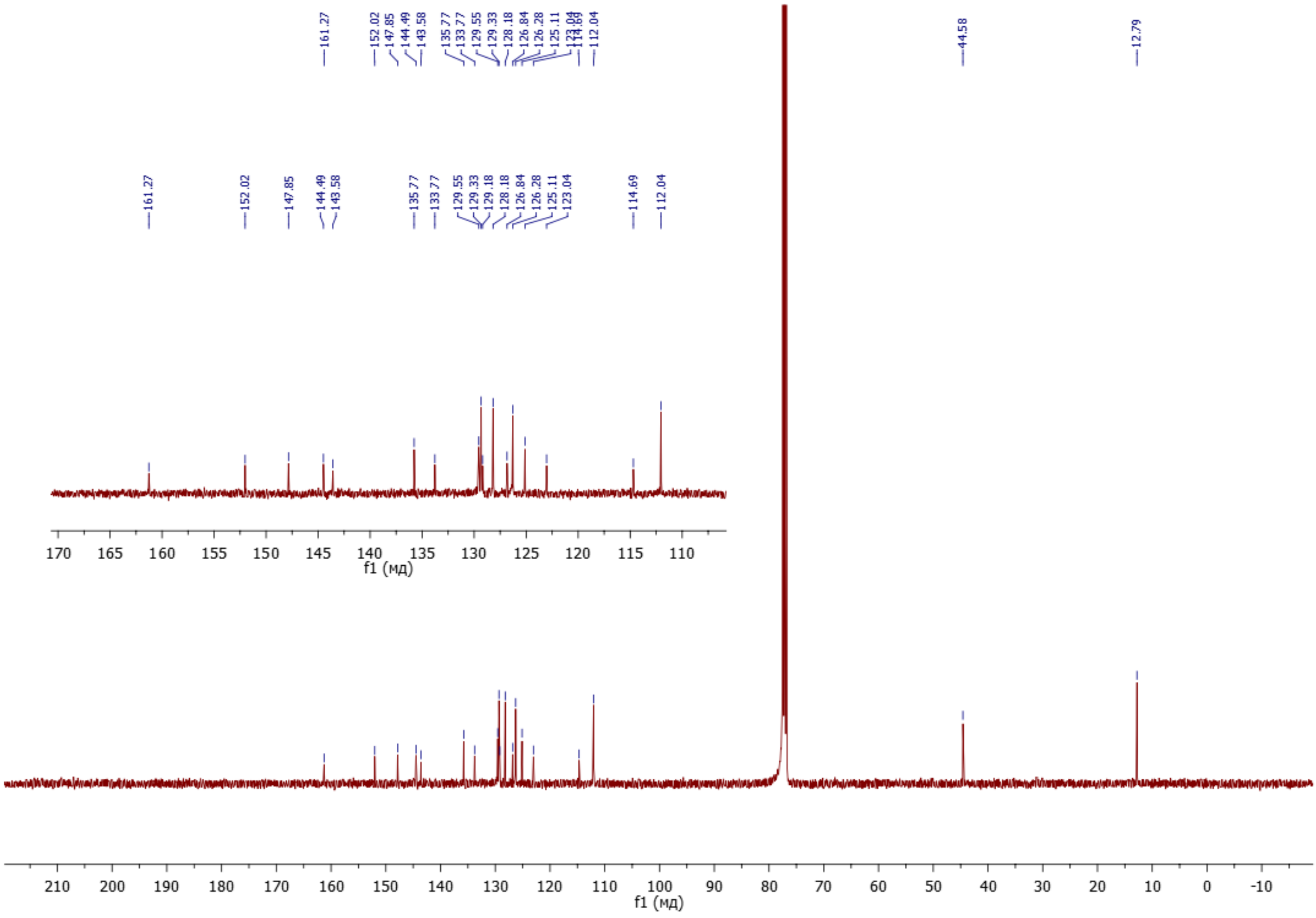

b

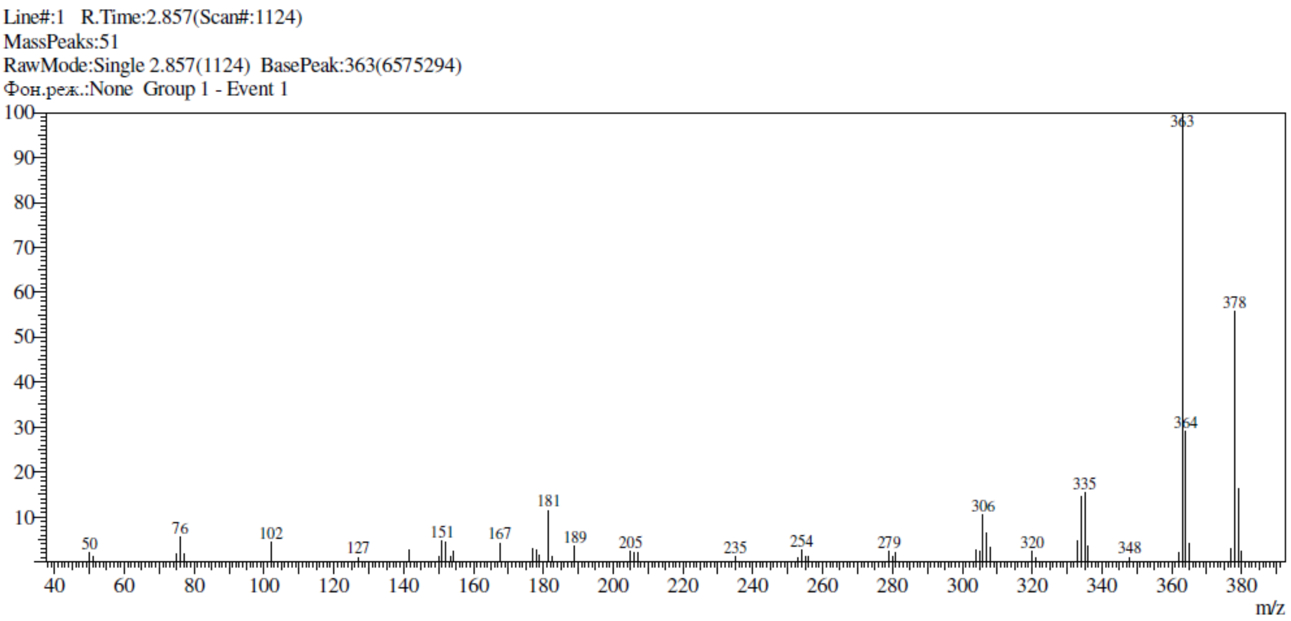

c

Figure S11. ^{1}H NMR spectrum (a) and ^{13}C NMR spectrum in DCCl3 (b); mass spectrum (c) of **8a**.

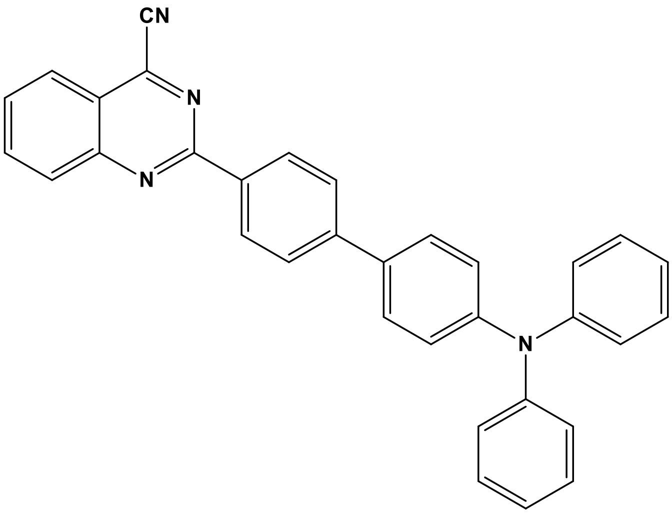

8b

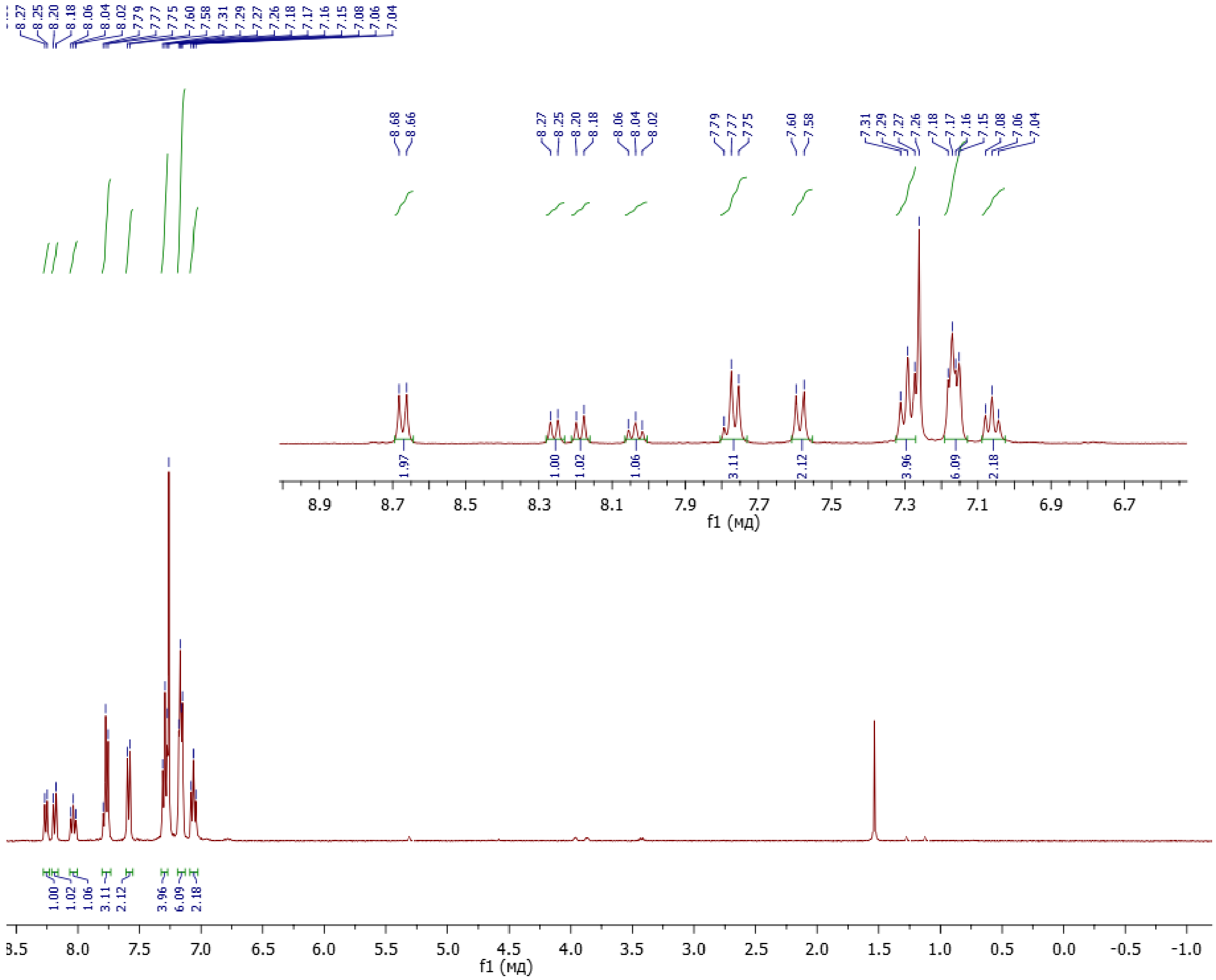

a

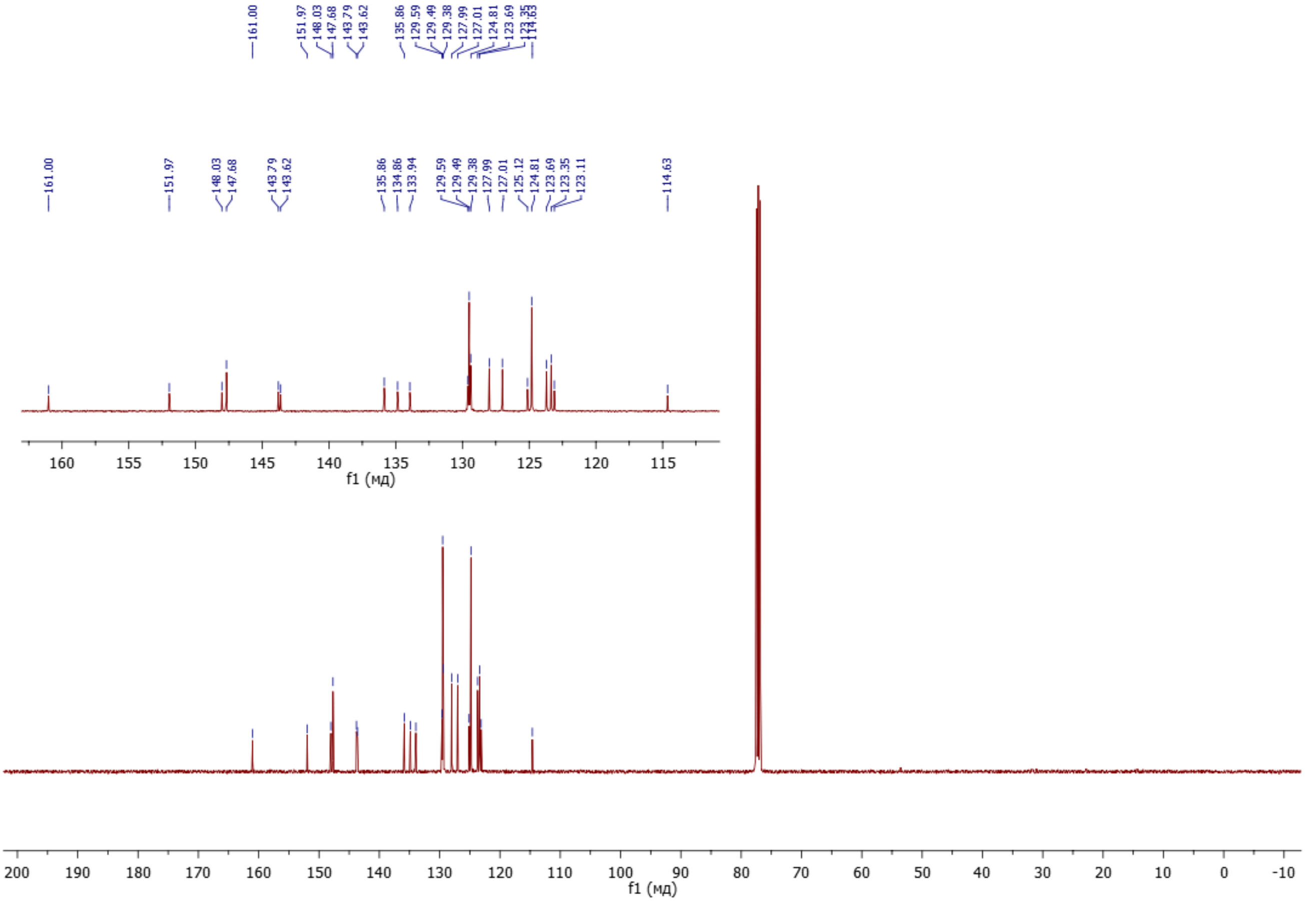

b

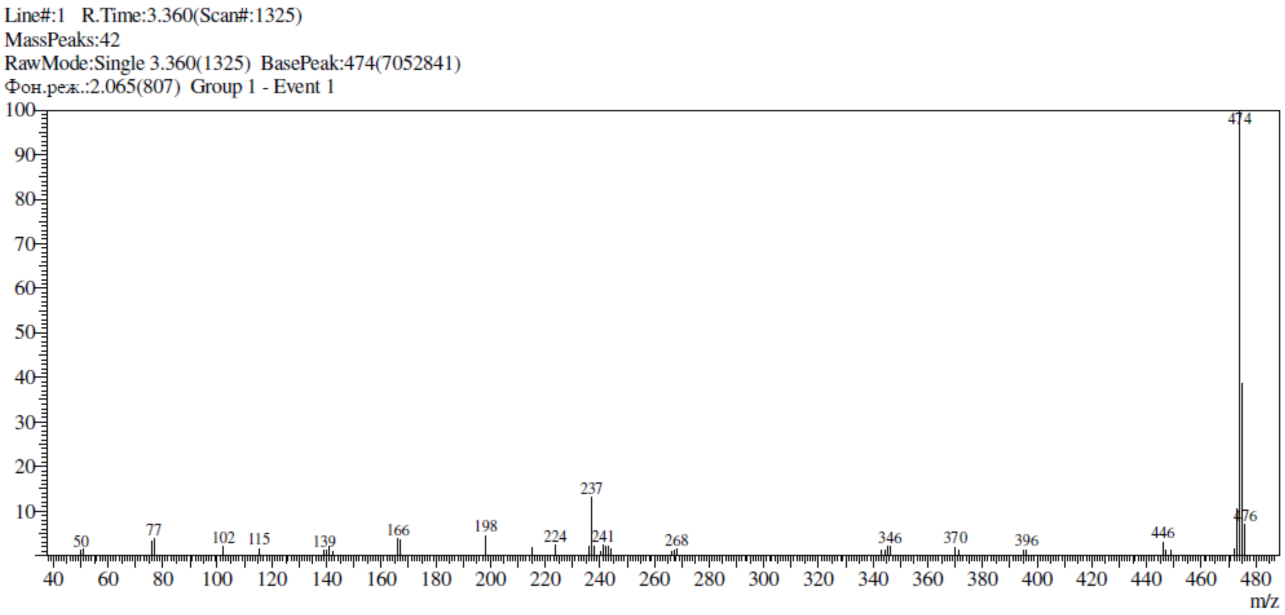

c

Figure S12. ^{1}H NMR spectrum (a) and ^{13}C NMR spectrum (b) in $DCCl_3$; mass spectrum (c) of **8b**.

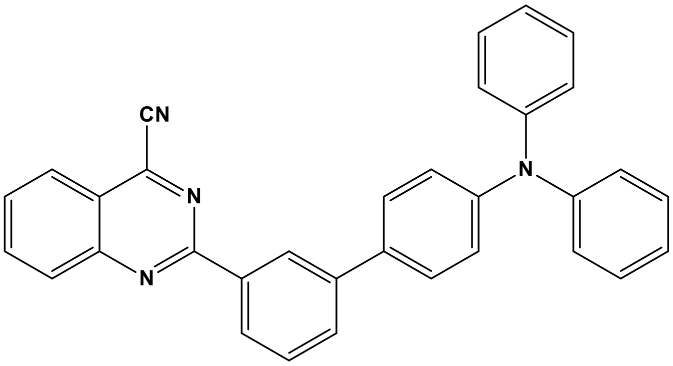

9

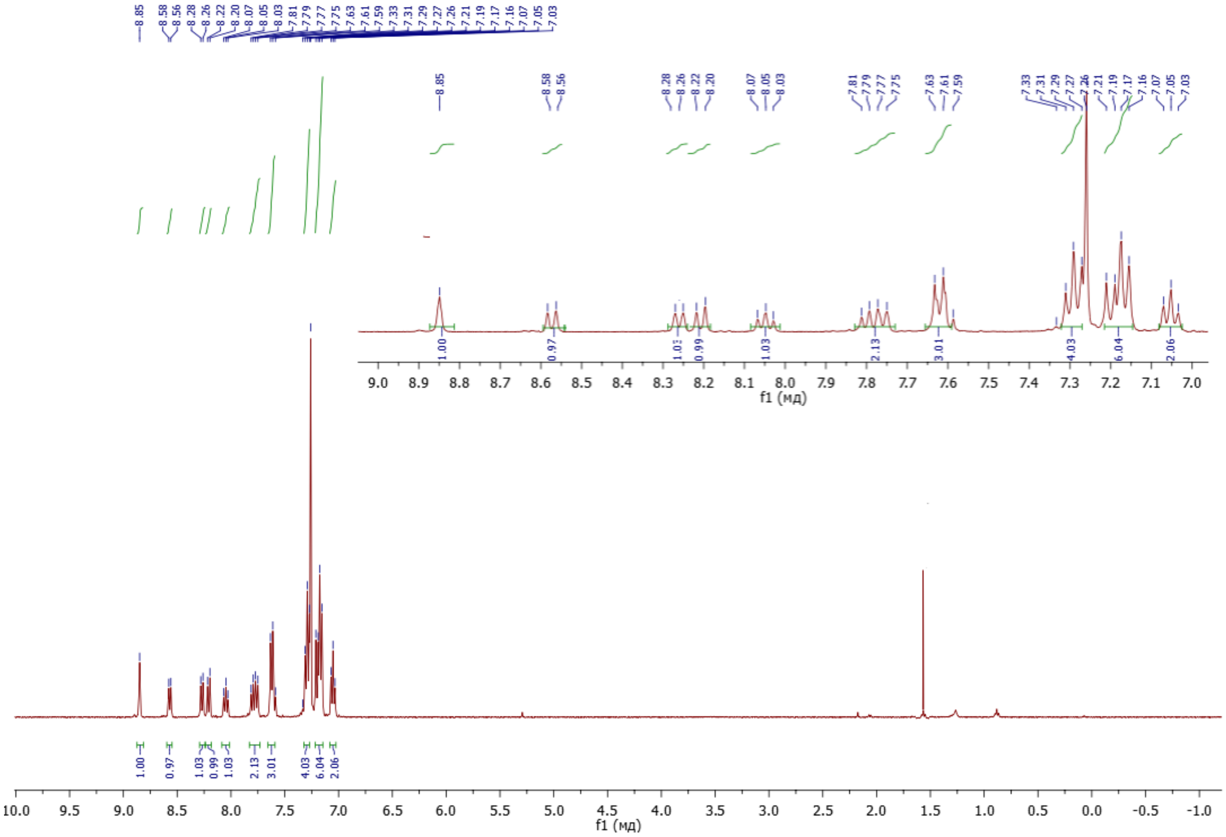

a

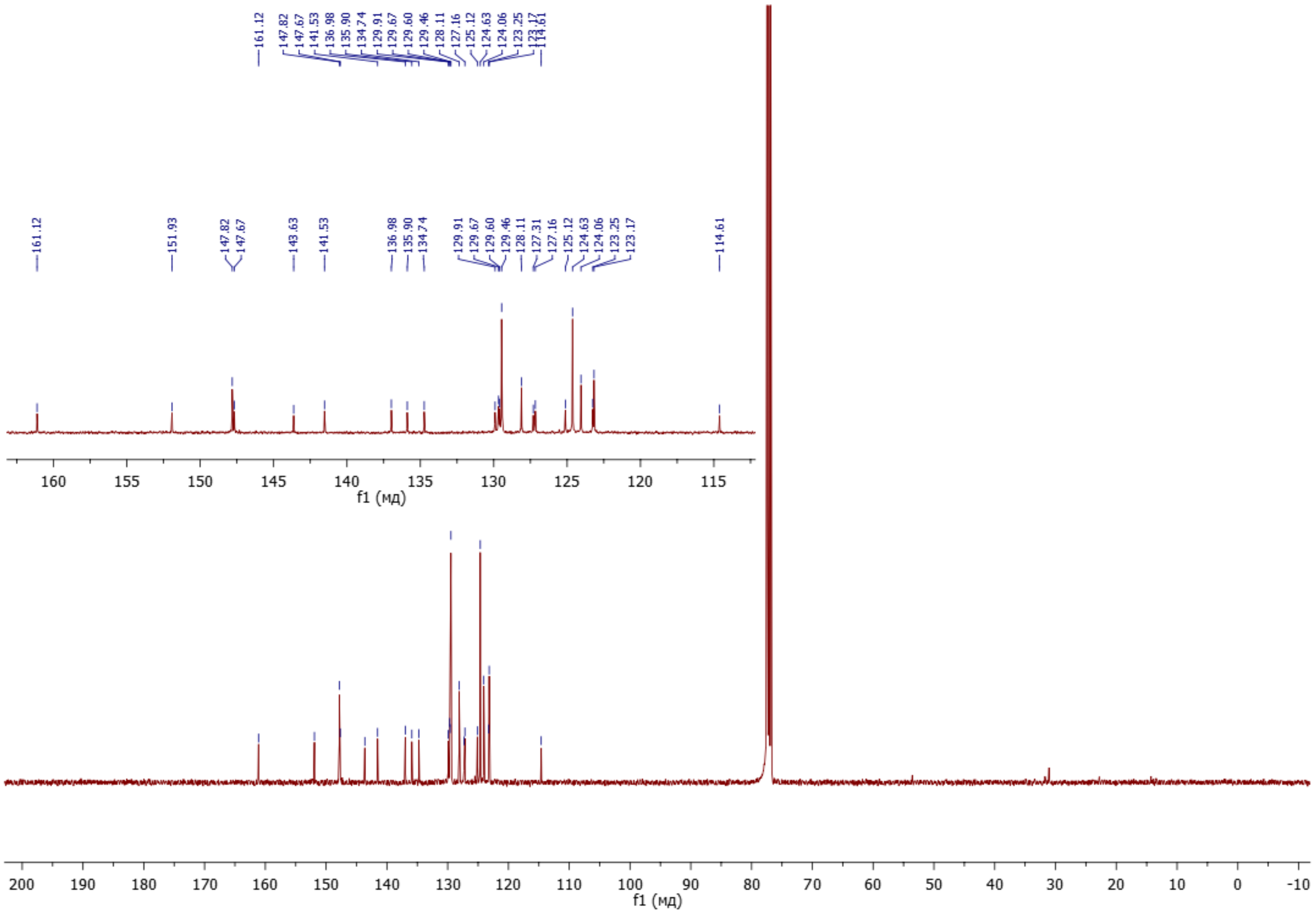

b

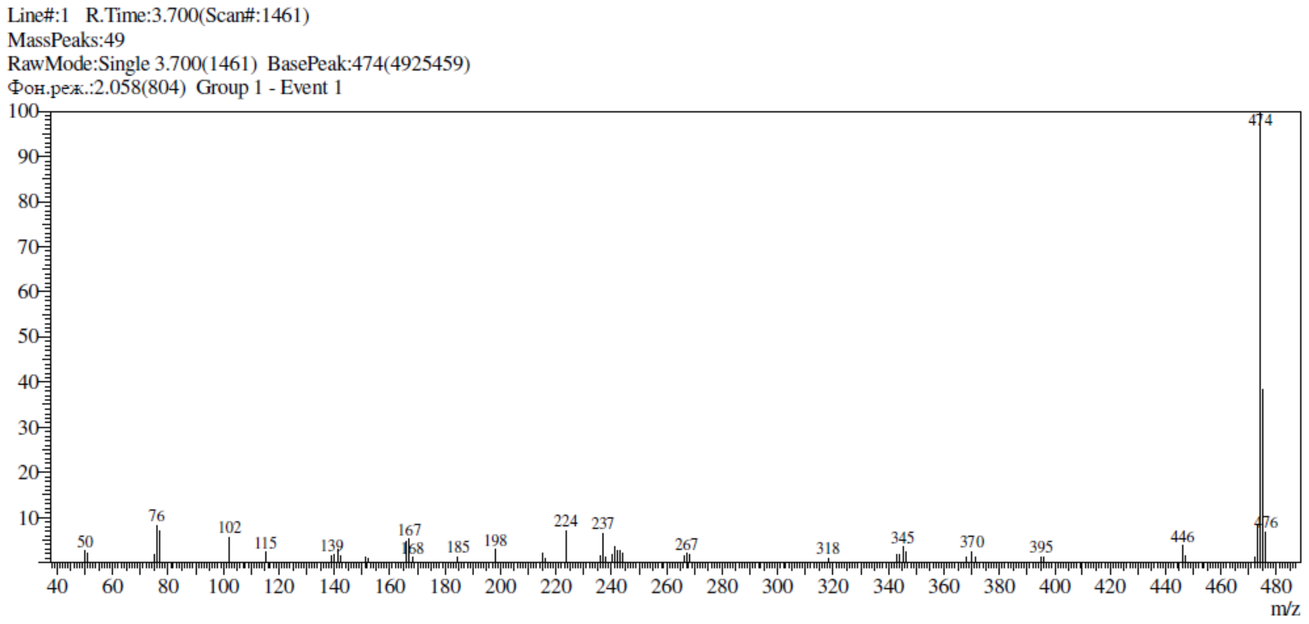

c

Figure S13. ^{1}H NMR spectrum (a) and ^{13}C NMR spectrum (b) in $DCCl_3$; mass spectrum (c) of **9**.

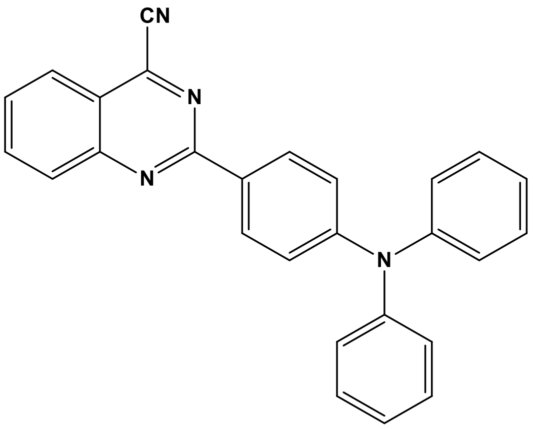
CN
N
N
N

11

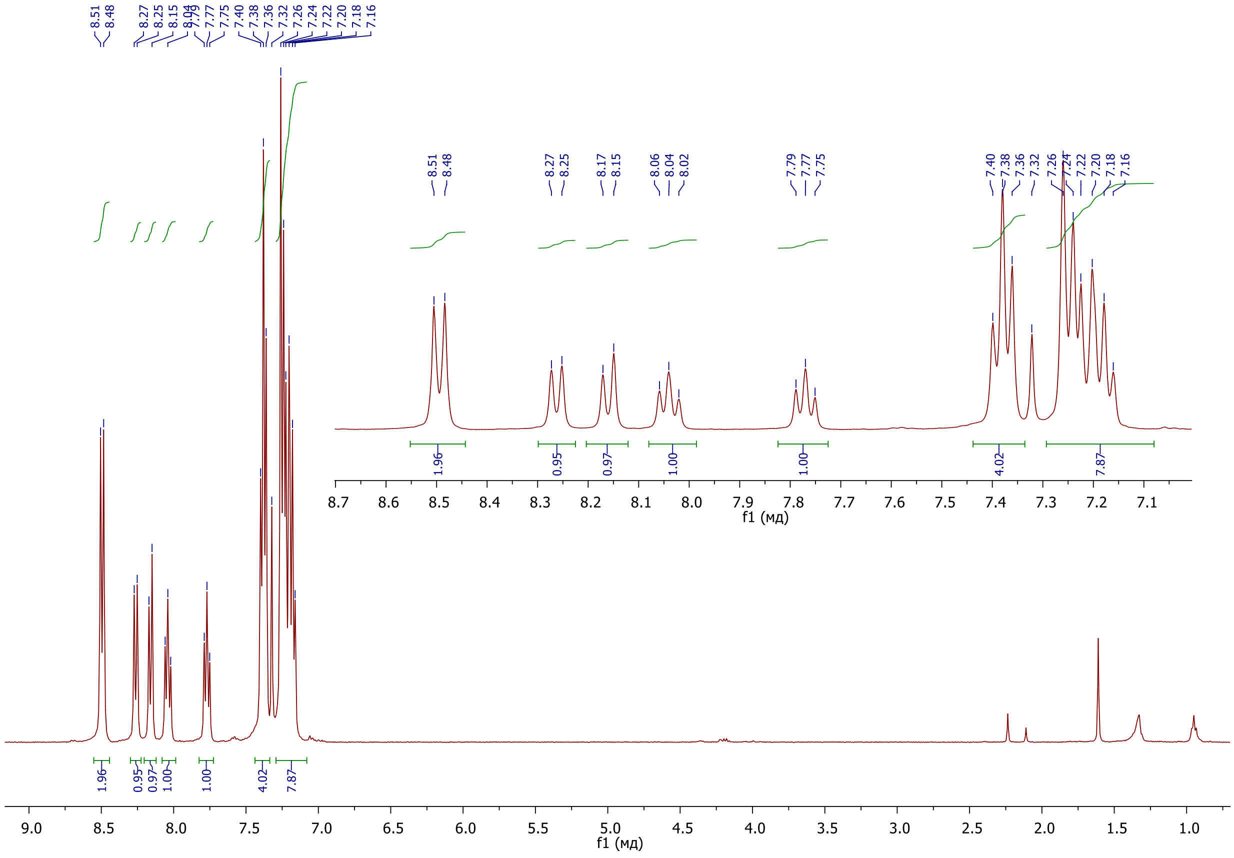
8.51
8.48
8.27
8.25
8.15
8.04
7.79
7.77
7.75
7.40
7.38
7.36
7.32
7.26
7.24
7.22
7.20
7.18
7.16
1.96
0.95
0.97
1.00
1.00
4.02
7.87
9.0 8.5 8.0 7.5 7.0 6.5 6.0 5.5 5.0 4.5 4.0 3.5 3.0 2.5 2.0 1.5 1.0
f1 (мд)
8.17
8.15
8.06
8.04
8.02
8.7 8.6 8.5 8.4 8.3 8.2 8.1 8.0 7.9 7.8 7.7 7.6 7.5 7.4 7.3 7.2 7.1
f1 (мд)

a

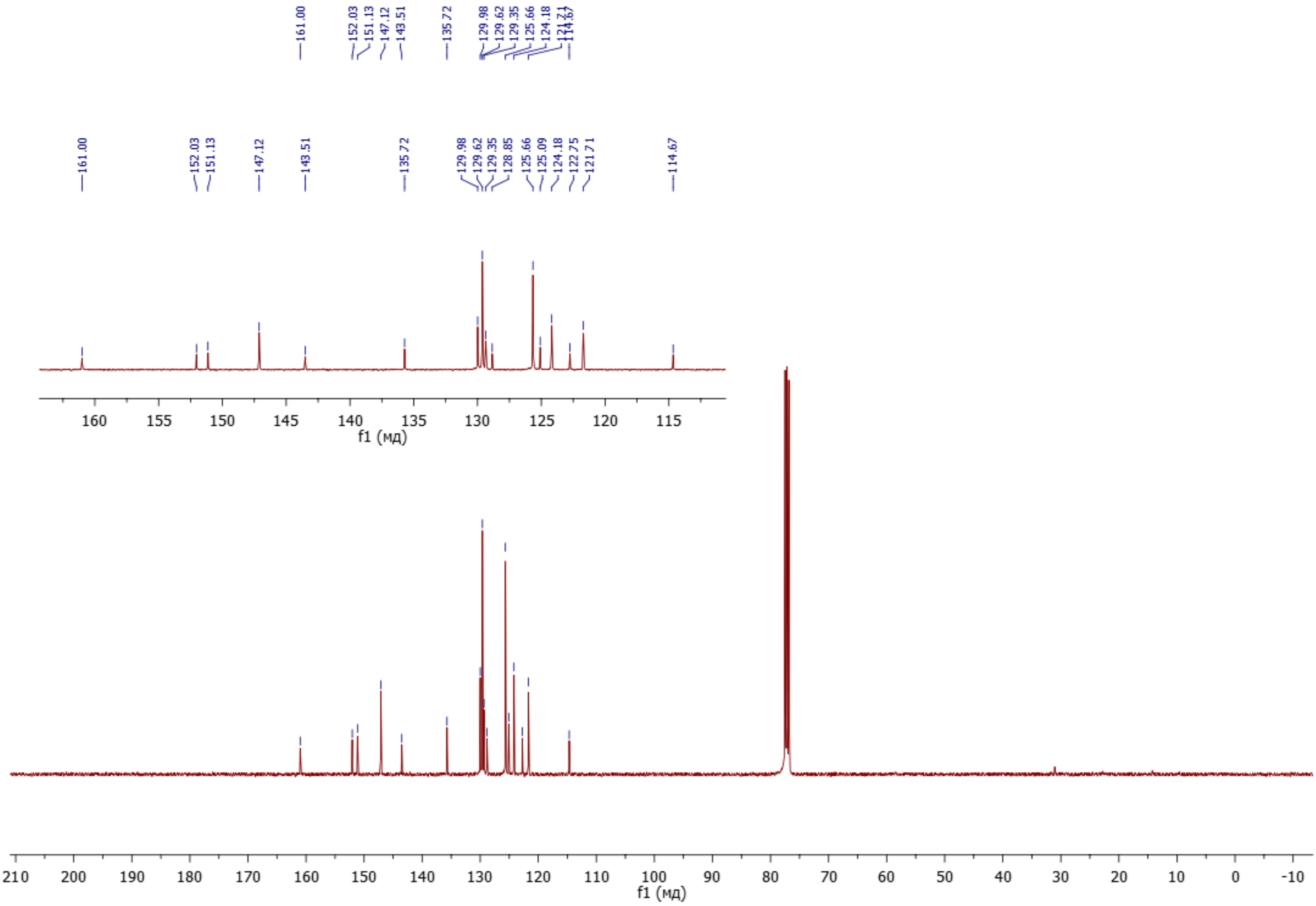

b

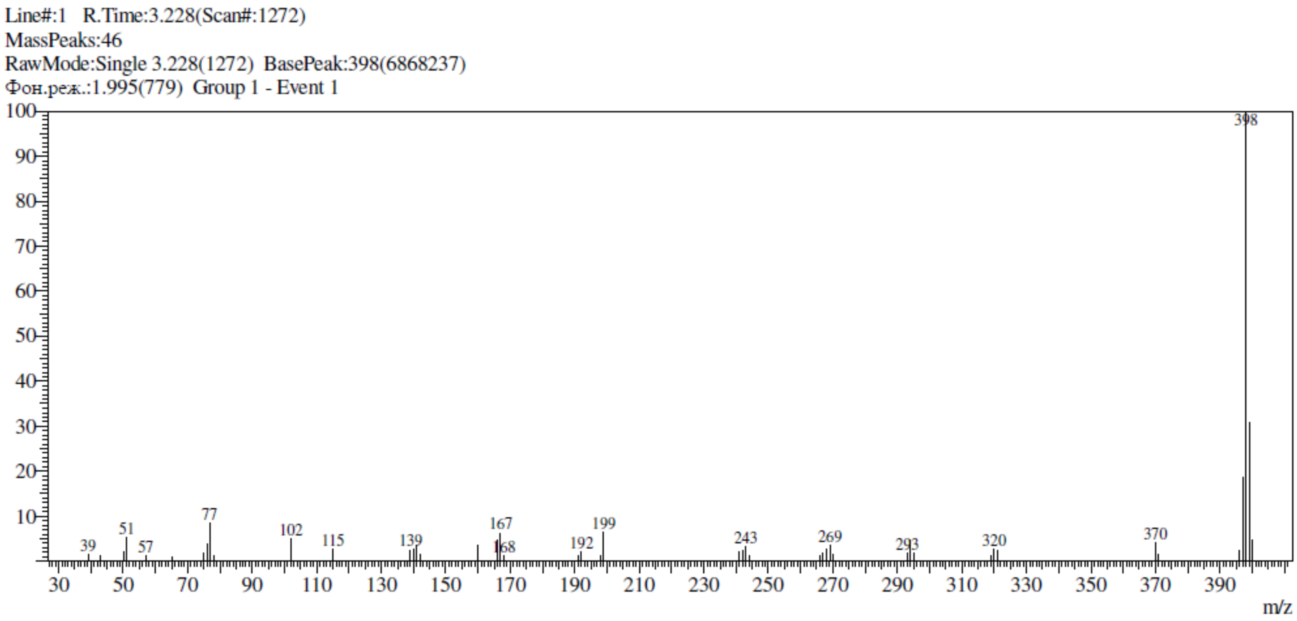

c

Figure S14. ^{1}H NMR spectrum (a) and ^{13}C NMR spectrum in $DCCl_3$ (b); mass spectrum (c) of **11**.

3. **Crystallographic data of compounds 11**

Table S1. Selected bond lengths of compound **11**.

**Bond**	**Bond length (Å)**	**Bond**	**Bond length (Å)**
N(3) – C(2)	1.374(2)	C(1) – C(5)	1.418(3)
N(3) – C(4)	1.314(3)	C(23) – C(24)	1.385(3)
N(1) – C(2)	1.325(3)	C(23) – C(28)	1.373(3)
N(1) – C(9)	1.368(3)	C(24) – C(25)	1.454(3)
N(4) – C(14)	1.402(3)	C(4) – C(10)	1.385(3)
N(4) – C(13)	1.433(3)	C(18) – C(19)	1.405(3)
N(4) – C(23)	1.430(2)	C(9) – C(8)	1.141(3)
C(12) – C(11)	1.391(3)	N(2) – C(10)	1.381(3)
C(12) – C(17)	1.376(3)	C(28) – C(27)	1.357(4)
C(11) – C(2)	1.469(3)	C(5) – C(6)	1.372(4)
C(11) – C(16)	1.396(3)	C(27) – C(26)	1.371(4)
C(14) – C(17)	1.401(3)	C(19) – C(20)	1.375(4)
C(14) – C(15)	1.398(3)	C(25) – C(26)	1.384(3)
C(13) – C(18)	1.382(3)	C(22) – C(21)	1.372(4)
C(13) – C(22)	1.371(3)	C(20) – C(21)	1.366(4)
C(15) – C(16)	1.381(3)	C(8) – C(7)	1.403(4)
C(1) – C(4)	1.415(3)	C(6) – C(7)	1.373(3)
C(1) – C(9)	1.409(3)		

Table S2. Selected bond angles of compound **11**.

**Angle**	**(°)**	**Angle**	(°)
C(4) – N(3) – C(2)	116.46(19)	N(1) – C(2) – N(3)	125.0(2)
C(2) – N(1) – C(9)	117.92(18)	N(1) – C(2) – C(11)	118.09(18)
C(14) – N(4) – C(13)	121.14(15)	C(15) – C(16) – C(11)	121.27(19)
C(14) – N(4) – C(23)	120.89(17)	C(25) – C(24) – C(23)	120.4(2)
C(23) – N(4) – C(13)	117.54(16)	N(3) – C(4) – C(1)	124.25(19)
C(17) – C(12) – C(11)	121.41(19)	N(3) – C(4) – C(10)	116.2(2)
C(12) – C(11) – C(2)	120.88(19)	C(1) – C(4) – C(10)	119.6(2)
C(12) – C(11) – C(16)	117.56(19)	C(13) – C(18) – C(19)	120.3(2)
C(16) – C(11) – C(2)	121.53(18)	N(1) – C(9) – C(1)	121.4(2)
C(17) – C(14) – N(4)	120.45(17)	N(1) – C(9) – C(8)	119.3(2)
C(15) – C(14) – N(4)	122.05(18)	C(8) – C(9) – C(1)	119.2(2)
C(15) – C(14) – C(17)	117.49(19)	C(27) – C(28) – C(23)	120.0(2)

C(12) – C(17) – C(14)	121.00(18)	N(2) – C(10) – C(4)	179.1(3)
C(18) – C(13) – N(4)	119.4(2)	C(6) – C(5) – C(1)	119.6(2)
C(22) – C(13) – N(4)	120.6(2)	C(26) – C(27) – C(28)	120.6(2)
C(22) – C(13) – C(18)	119.9(2)	C(20) – C(19) – C(18)	119.5(3)
C(16) – C(15) – C(14)	120.89(19)	C(24) – C(25) – C(26)	120.5(3)
C(4) – C(1) – C(5)	125.3(2)	C(13) – C(22) – C(21)	119.6(2)
C(9) – C(1) – C(4)	114.9(2)	C(19) – C(20) – C(21)	120.1(2)
C(9) – C(1) – C(5)	119.8(2)	C(27) – C(26) – C(25)	119.4(2)
C(24) – C(23) – N(4)	121.8(2)	C(20) – C(21) – C(22)	120.6(2)
C(24) – C(23) – C(28)	118.92(19)	C(7) – C(8) – C(9)	119.8(3)
C(28) – C(23) – N(4)	119.2(2)	C(5) – C(6) – C(7)	120.6(3)
N(3) – C(2) – C(11)	116.89(19)	C(8) – C(7) – C(6)	121.1(3)

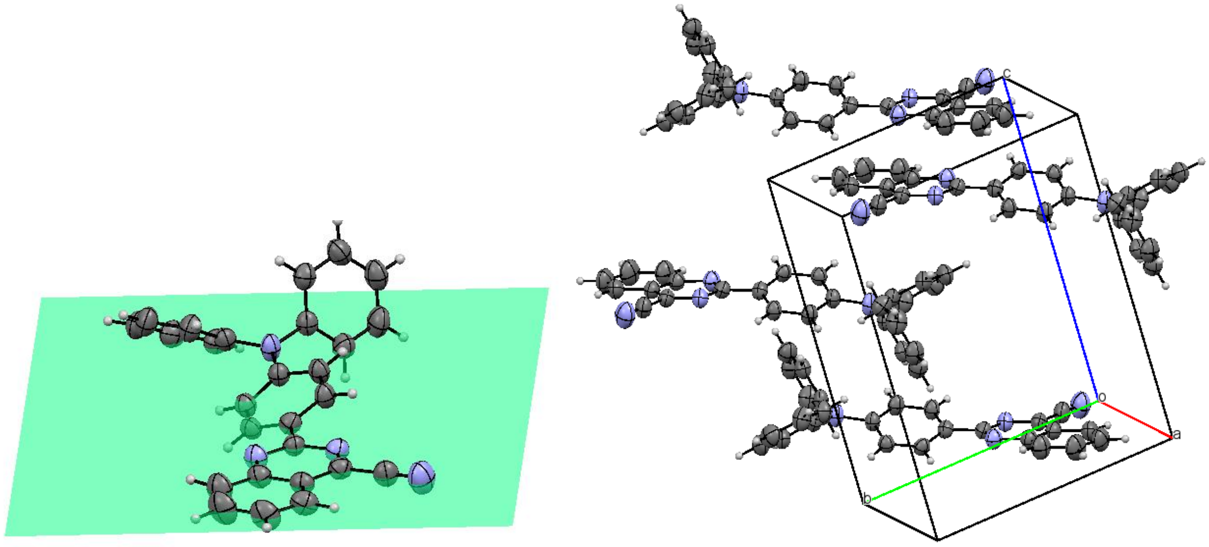

a b

Figure S15. Planarity (a) and packing (b) of compounds **11.**

4. Absorption, excitation and emission spectra of chromophores in toluene and MeCN

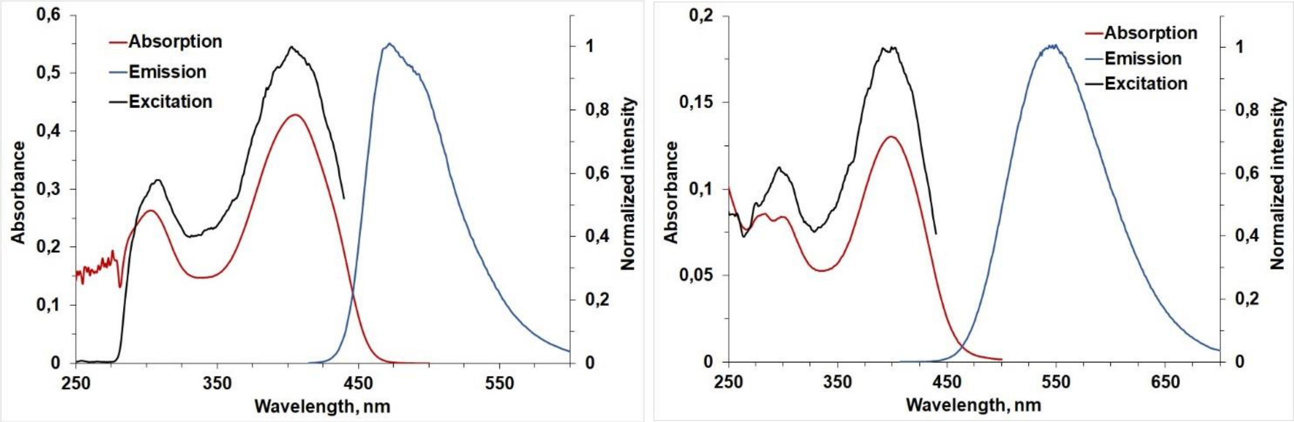

a b

Figure S16. Absorption, excitation and emission spectra of chromophore **4b** in toluene (a) and MeCN (b).

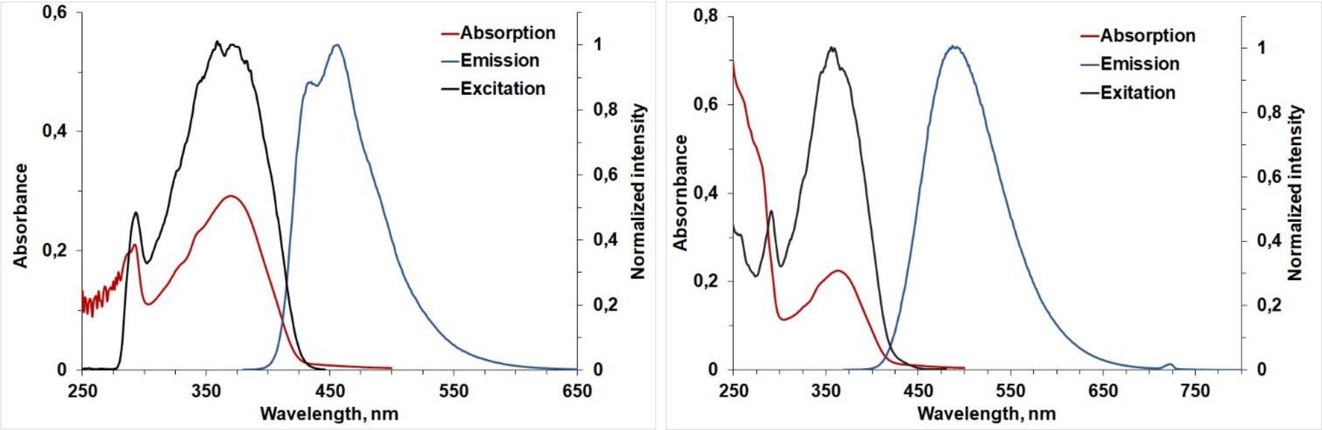

a b

Figure S17. Absorption, excitation and emission spectra of chromophore **4c** in toluene (a) and MeCN (b).

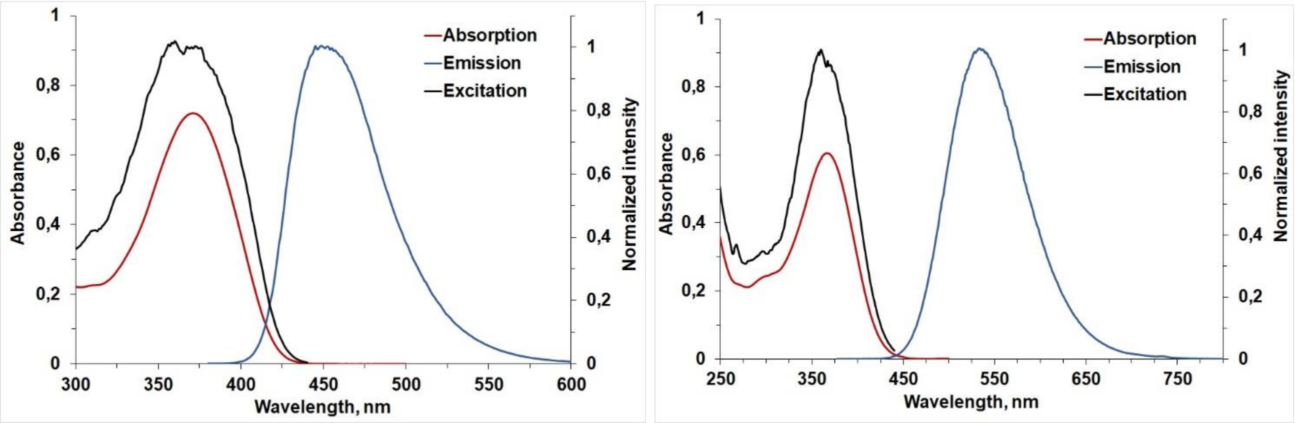

a b

Figure S18. Absorption, excitation and emission spectra of chromophore **5a** in toluene (a) and MeCN (b).

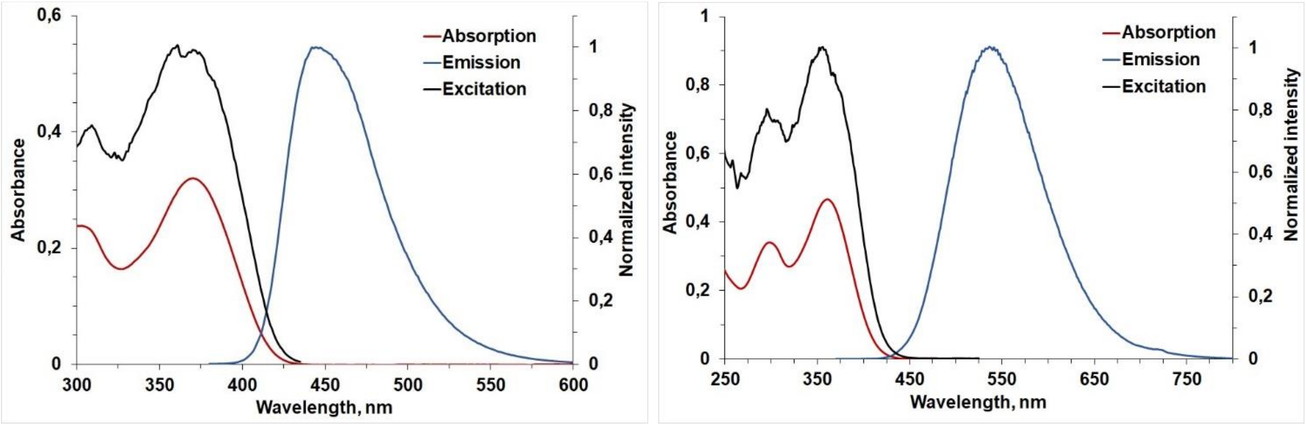

a b

Figure S19. Absorption, excitation and emission spectra of chromophore **5b** in toluene (a) and MeCN (b).

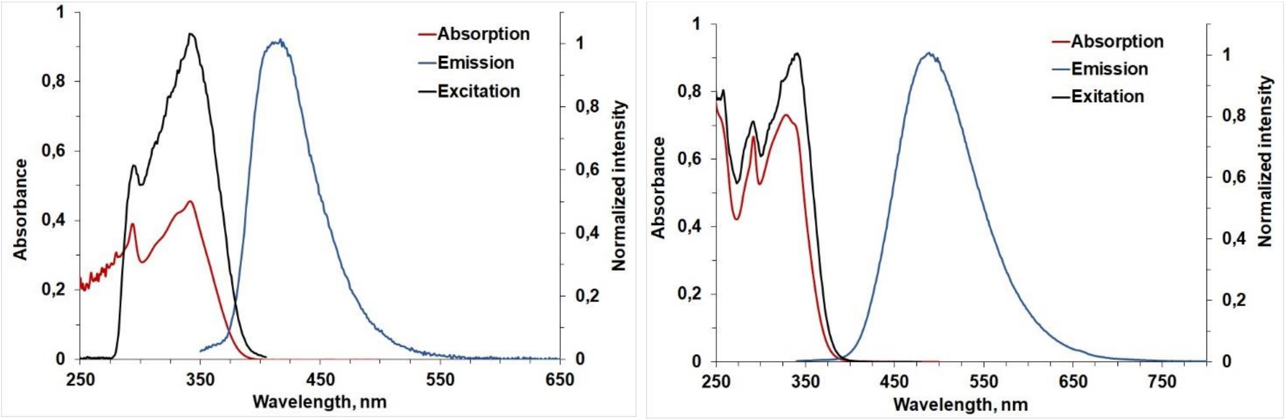

a b

Figure S20. Absorption, excitation and emission spectra of chromophore **5c** in toluene (a) and MeCN (b).

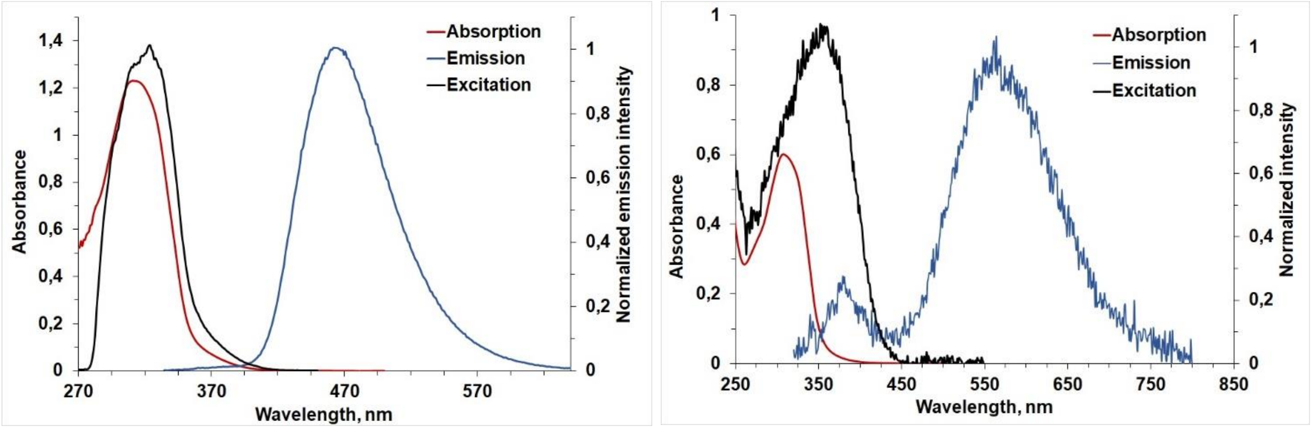

a b

Figure S21. Absorption, excitation and emission spectra of chromophore **6a** in toluene (a) and MeCN (b).

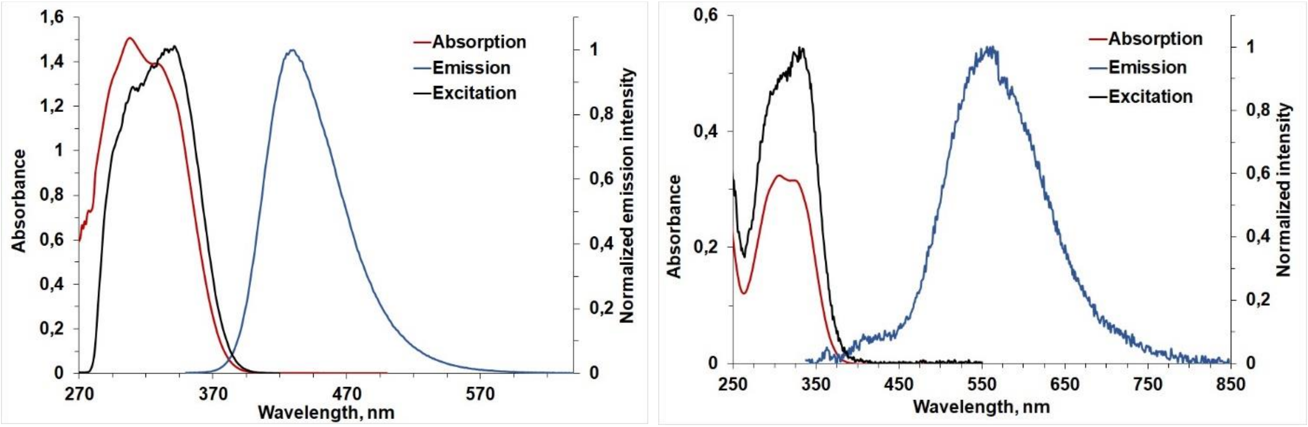

a b

Figure S22. Absorption, excitation and emission spectra of chromophore **6b** in toluene (a) and MeCN (b).

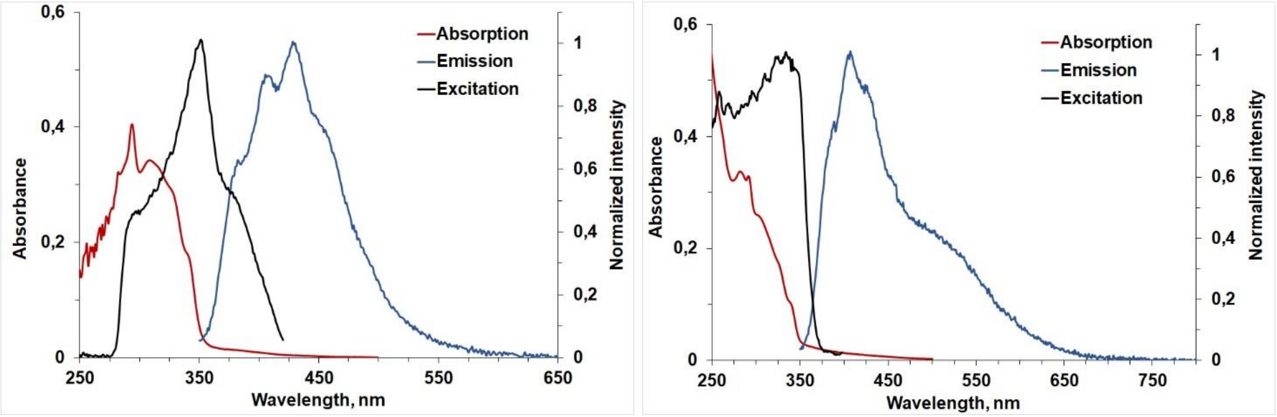

a b

Figure S23. Absorption, excitation and emission spectra of chromophore **6c** in toluene (a) and MeCN (b).

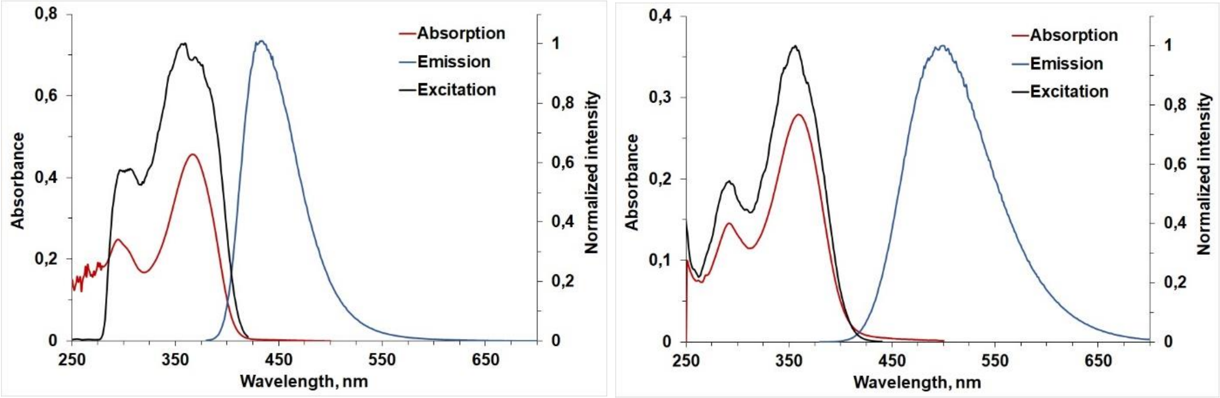

a b

Figure S24. Absorption, excitation and emission spectra of chromophore **10** in toluene (a) and MeCN (b).

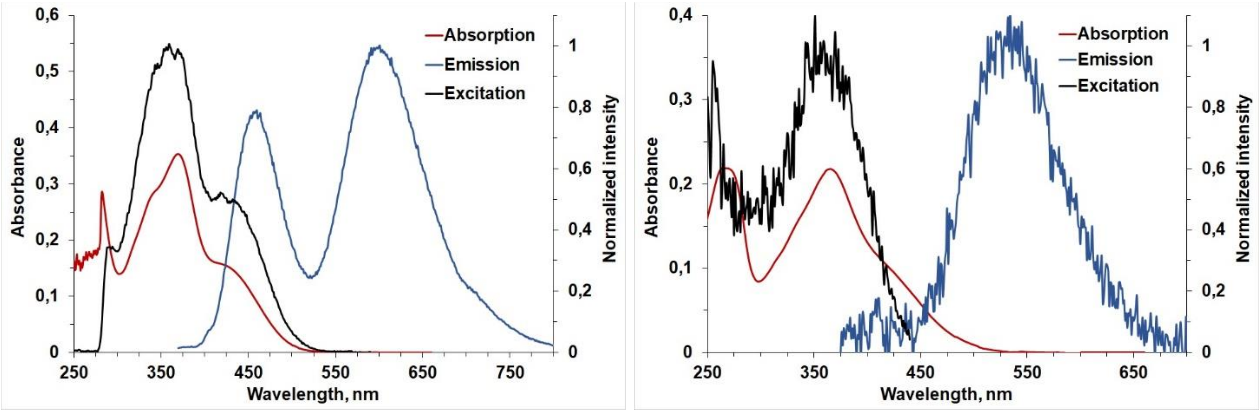

a b

Figure S25. Absorption, excitation and emission spectra of chromophore **8a** in toluene (a) and MeCN (b).

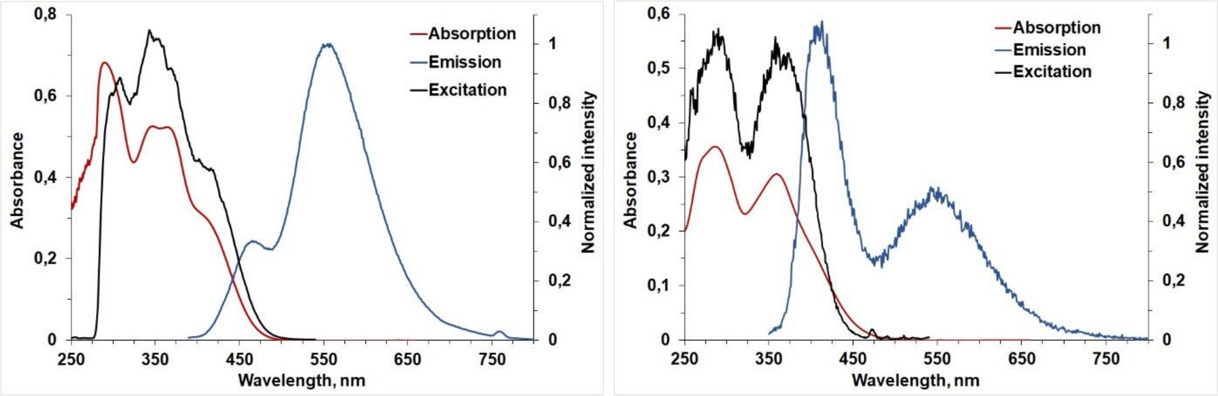

a b

Figure S26. Absorption, excitation and emission spectra of chromophore **8b** in toluene (a) and MeCN (b).

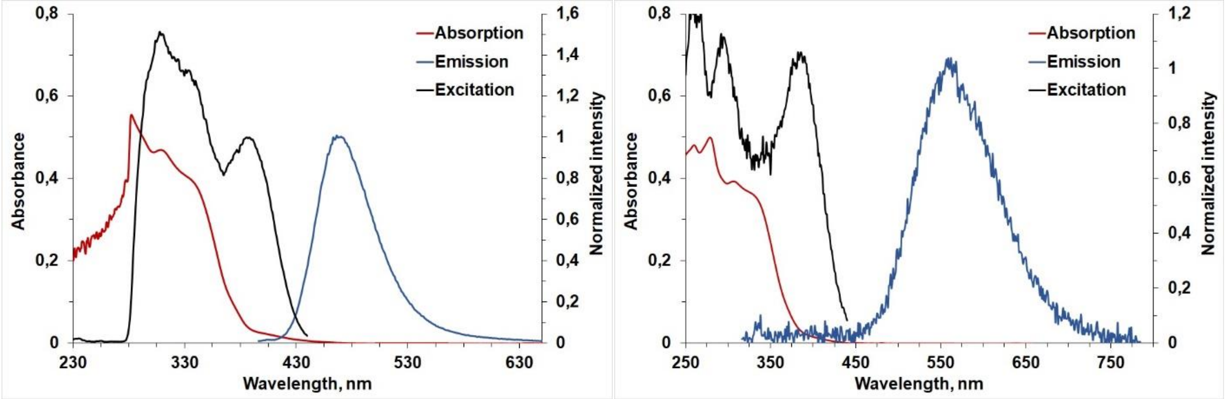

a b

Figure S27. Absorption, excitation and emission spectra of chromophore **9** in toluene (a) and MeCN (b).

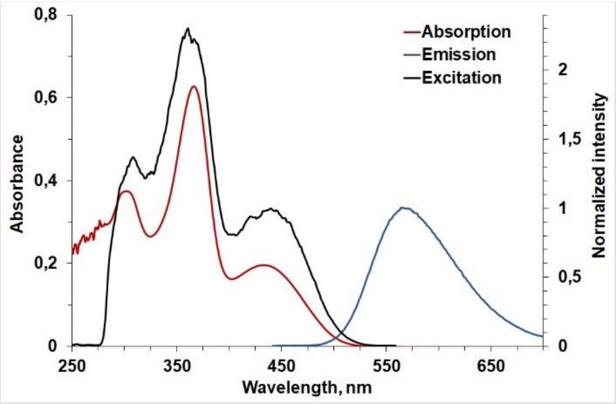

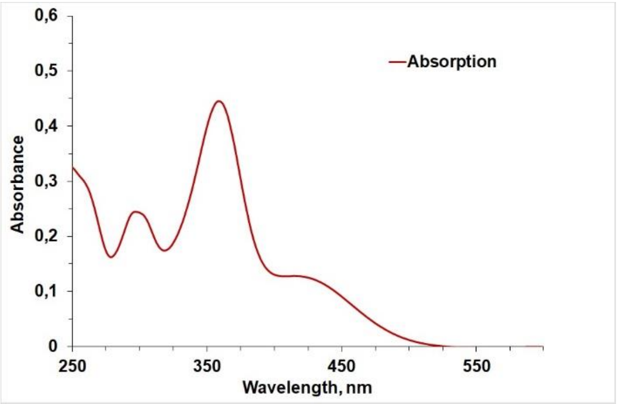

a b

Figure S28. Absorption, excitation and emission spectra of chromophore **11** in toluene (a) and absorption spectrum in MeCN (b).

5. Time-resolved fluorescence emission measurements

Table S3. Detailed data of the fluorescence lifetime measurements of **4a-c**, **5a-c**, **6a-c**, **10**, **7a-c**, **8a,b**, **9, 11**: τ – lifetime, *f* - fractional contribution, τ_{avg} – average lifetime, χ^2 - chi-squared distribution.

Compound	Solvent	λ_{em}, [nm]	τ_1, [ns]	f_1, %	τ_2, [ns]	f_2, %	τ_3, [ns]	f_3, %	τ_{avg}, [ns]	χ^2
**4a**	Toluene	490	1.53	23.4	2.06	76.6			**1.94**	**1.067**
	MeCN	541	2.07	17.1	2.84	82.9			**2.71**	**1.170**
**4b**	Toluene	470	1.59	33.1	2.05	66.9			**1.90**	**1.131**
	MeCN	545	2.01	7.7	3.36	92.3			**3.25**	**1.091**
**4c**	Toluene	430<br>456	0.54<br>0.54	77.2<br>78.2	1.46<br>1.50	22.8<br>21.8			**0.75**<br>**0.75**	**1.029**<br>**1.339**
	MeCN	490	1.11	6.6	2.65	93.4			**2.54**	**1.094**
**5a**	Toluene	450	0.93	27.5	1.55	72.5			**1.38**	**1.126**
	MeCN	535	2.49	83.9	3.40	16.1			**2.64**	**1.164**
**5b**	Toluene	445	1.38	45.8	1.80	54.2			**1.61**	**1.180**
	MeCN	535	1.88	4.8	3.30	95.2			**3.22**	**1.095**
**5c**	Toluene	415	0.41	89.4	2.38	10.6			**0.62**	**1.254**
	MeCN	490			3.66	100			**3.66**	**1.186**
**6a**	Toluene	460	1.88	7.0	8.92	93.0			**8.43**	**1.097**
	MeCN	Can not be detected								
**6b**	Toluene	430	1.69	10.0	5.50	90.0			**5.12**	**1.128**
	MeCN	565			5.94	100			**5.94**	**1.117**
**6c**	Toluene	430	1.77	75.8	4.77	24.2			**2.49**	**1.119**
	MeCN	408	2.16	24.3	4.96	27.2	12.34	48.5	**7.86**	**0.934**
**10**	Toluene	430	2.10	100					**2.10**	**1.049**
	MeCN	500	1.38	1.7	4.86	98.3			**4.80**	**1.091**
**7a**	Toluene	471	1.25	29.9	2.10	68.0	12.20	2.1	**2.06**	**1.080**
	MeCN	539	1.28	4.9	3.01	90.6	7.52	4.5	**3.13**	**1.198**
**7b**	Toluene	480<br>621	1.40<br>1.60	86.2<br>87.7	2.58<br>2.64	13.8<br>12.3			**1.56**<br>**1.73**	**1.024**<br>**1.044**
	MeCN	525	1.69	7.3	2.96	92.7			**2.87**	**1.011**
**7c**	Toluene	541	1.52	4.7	4.93	95.3			**4.77**	**1.079**
	MeCN	491	2.32	75.7	3.94	24.3			**2.71**	**1.125**
**8a**	Toluene	460<br>600	1.55	58	3.14<br>2.95	42<br>100			**2.22**<br>**2.95**	**1.082**<br>**1.170**
	MeCN	530	0.79	21.7	2.45	72.6	6.70	5.7	**2.33**	**1.060**
**8b**	Toluene	465<br>555	1.09	12.3	2.50<br>1.32	79.1<br>2.1	5.39<br>7.13	8.6<br>97.9	**2.58**<br>**7.01**	**1.093**<br>**1.015**
	MeCN	400<br>540	1.25<br>1.54	90.0<br>21.1	2.23<br>3.00	10.0<br>78.9			**1.35**<br>**2.69**	**1.159**<br>**1.090**
**9**	Toluene	468	1.64	7.9	3.20	92.1			**3.08**	**1.100**
	MeCN	560	1.55	13.4	3.31	86.6			**3.07**	**1.002**
**11**	Toluene	570					9.68	100	**9.68**	**1.043**
	MeCN	Not emissive								

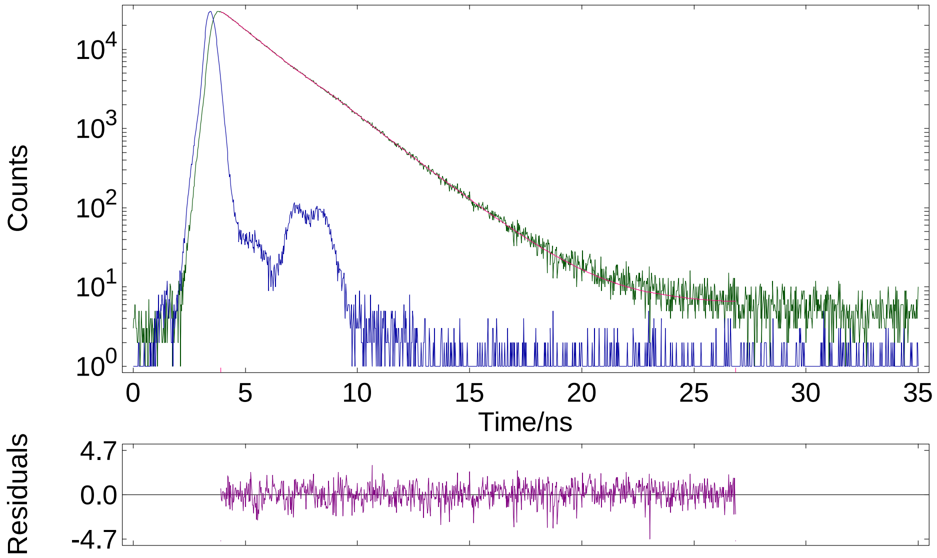

a

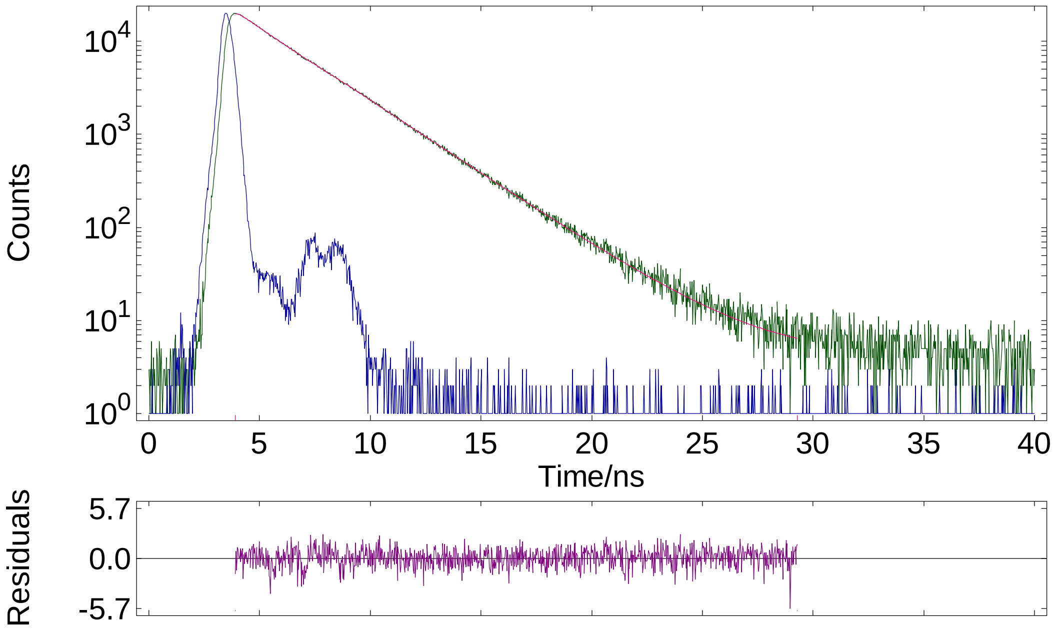

b

Figure S29. Time-resolved fluorescence lifetime decay profile of **4a** (a) in toluene, instrumental response function (IRF, blue). λ_{ex} = 375 nm, λ_{em} = 490 nm; (b) in MeCN, IRF (blue). λ_{ex} = 375 nm, λ_{em} = 541 nm.

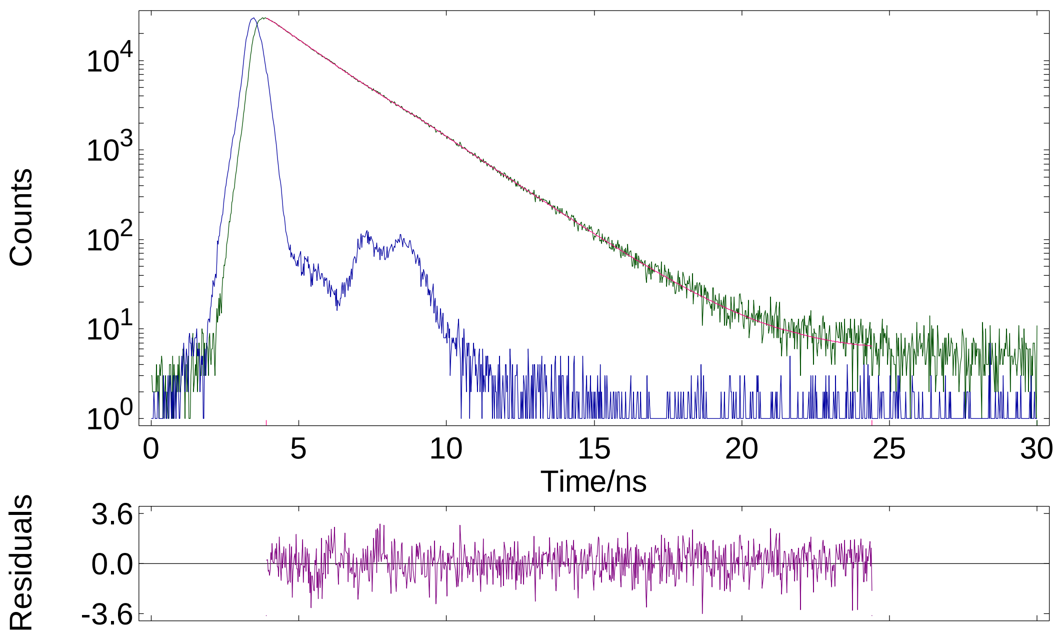

a

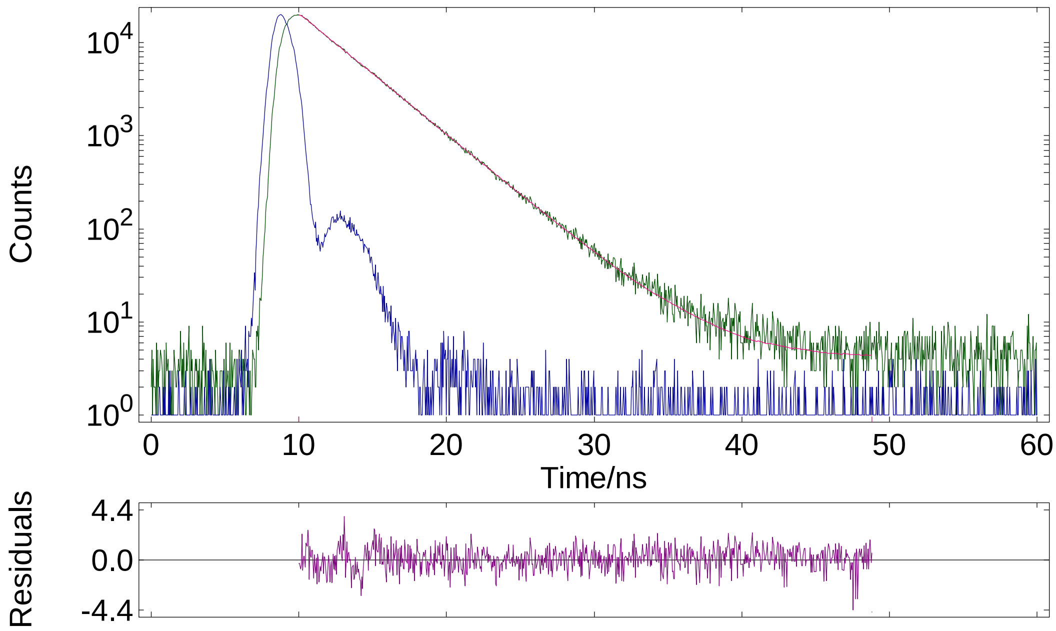

b

Figure S30. Time-resolved fluorescence lifetime decay profile of **4b** (a) in toluene, IRF (blue). λ_{ex} = 375 nm, λ_{em} = 470 nm; (b) in MeCN, IRF (blue). λ_{ex} = 375 nm, λ_{em} = 545 nm.

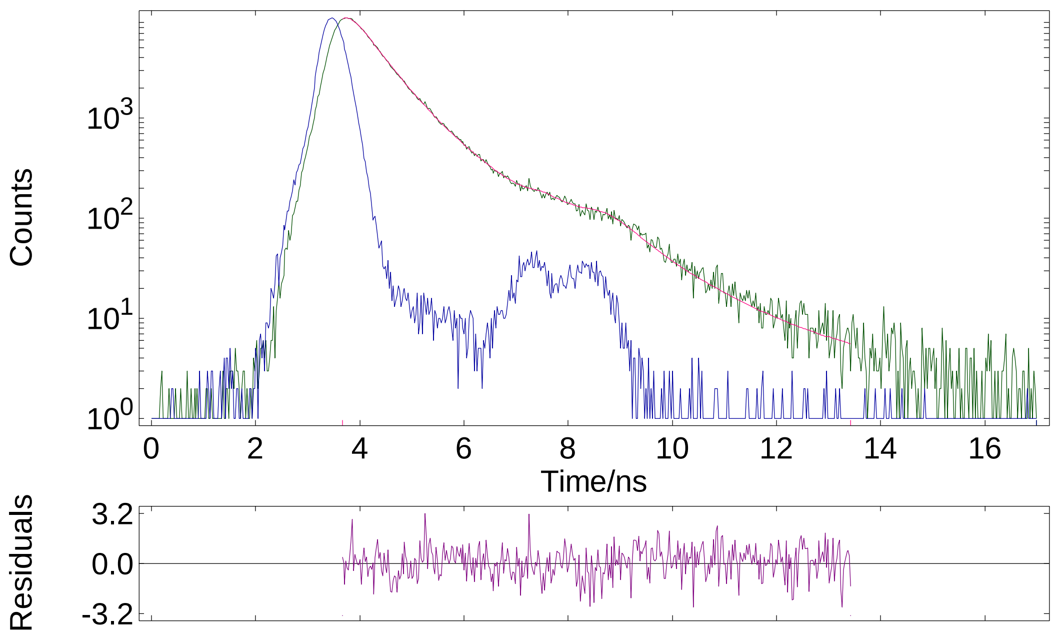
Counts
10^3
10^2
10^1
10^0
0 2 4 6 8 10 12 14 16
Time/ns
Residuals
3.2
0.0
-3.2

a

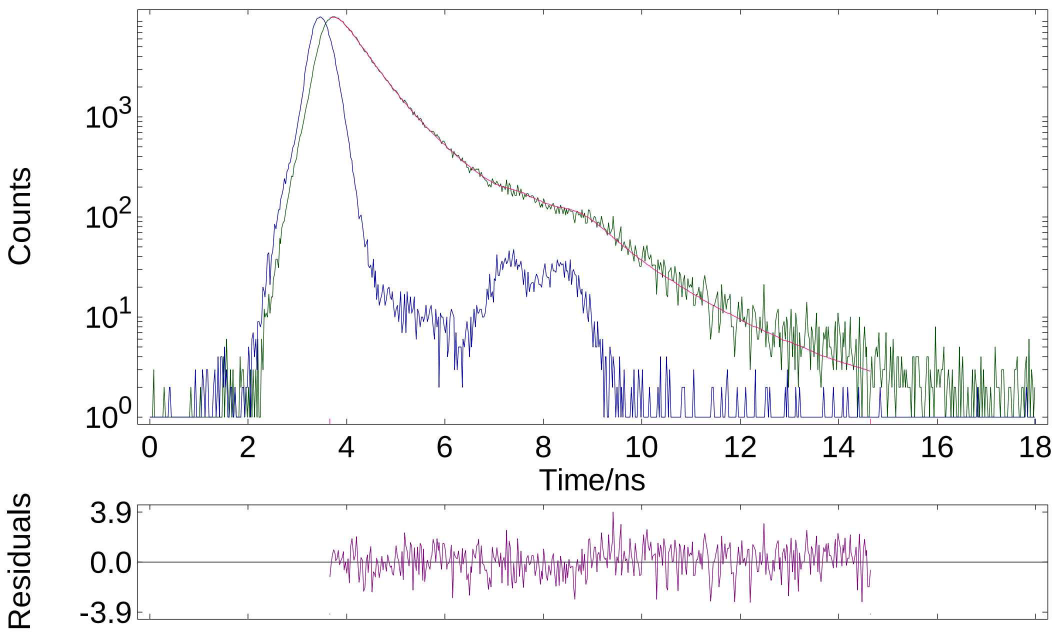
Counts
10^3
10^2
10^1
10^0
0 2 4 6 8 10 12 14 16 18
Time/ns
Residuals
3.9
0.0
-3.9

b

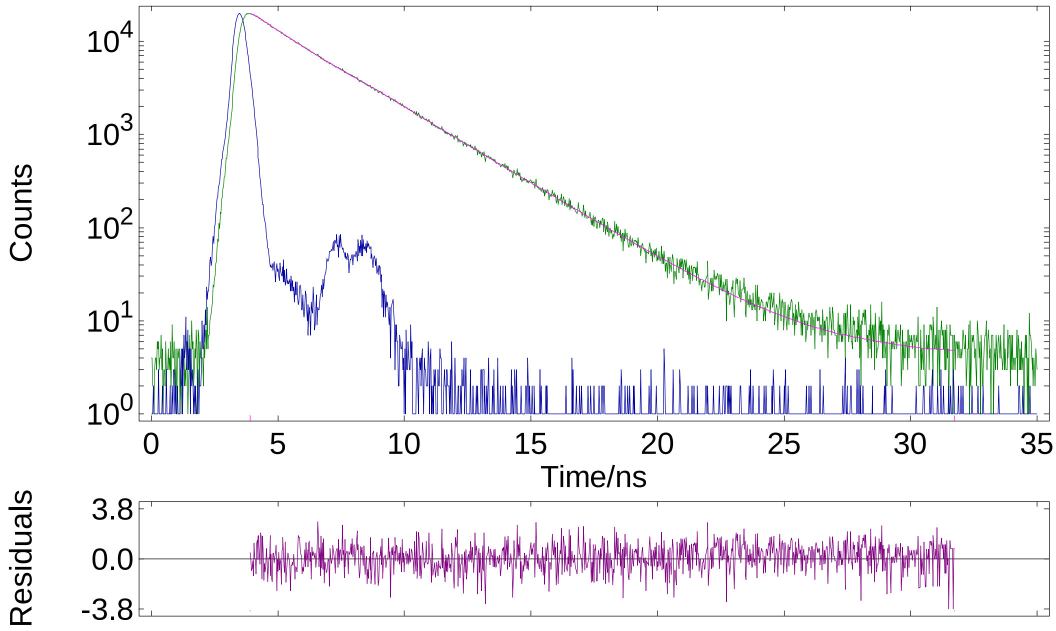

c

Figure 31. Time-resolved fluorescence lifetime decay profile of **4c** (a) in toluene, IRF (blue). λ_{ex} = 375 nm, λ_{em} = 430 nm; (b) in toluene, IRF (blue). λ_{ex} = 375 nm, λ_{em} = 456 nm; (c) in MeCN, IRF (blue). λ_{ex} = 375 nm, λ_{em} = 490 nm.

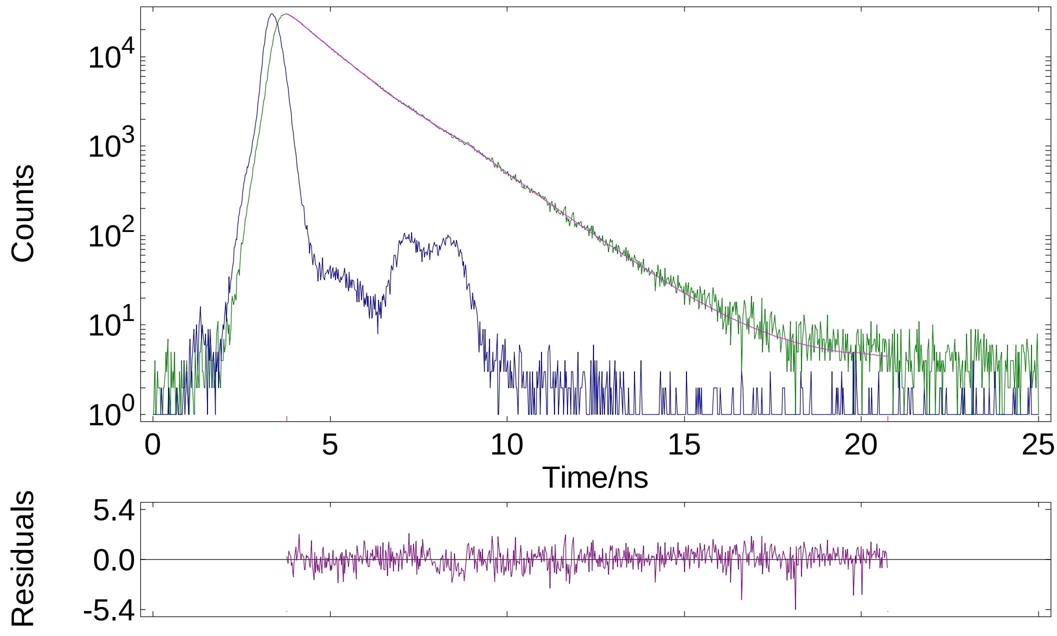

a

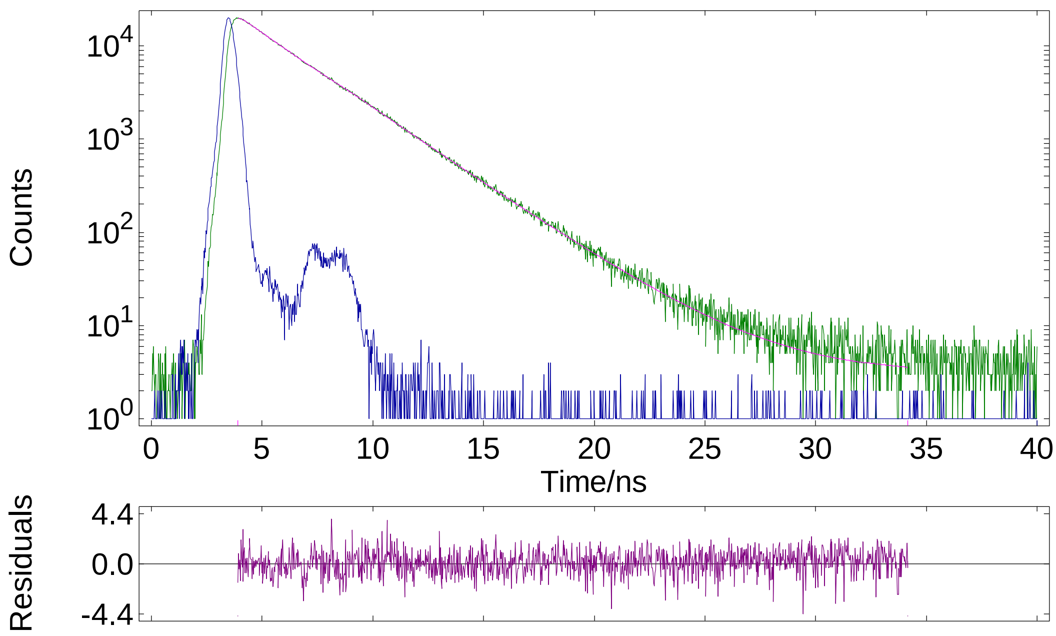

b

Figure 32. Time-resolved fluorescence lifetime decay profile of **5a** (a) in toluene, IRF (blue). λ_{ex} = 375 nm, λ_{em} = 450 nm; (b) in MeCN, IRF (blue). λ_{ex} = 375 nm, λ_{em} = 535 nm.

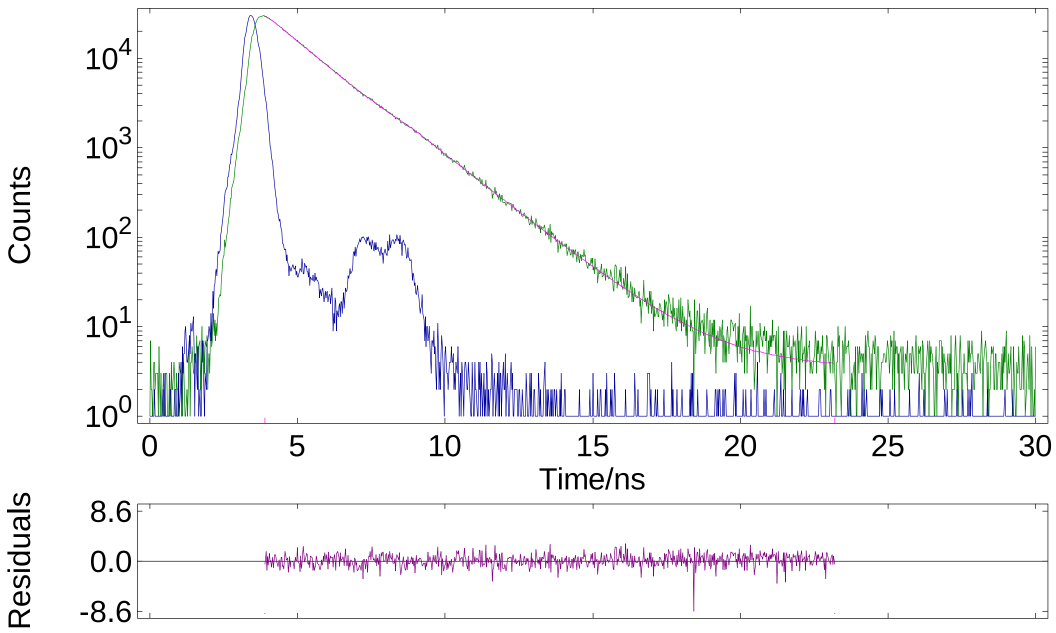

a

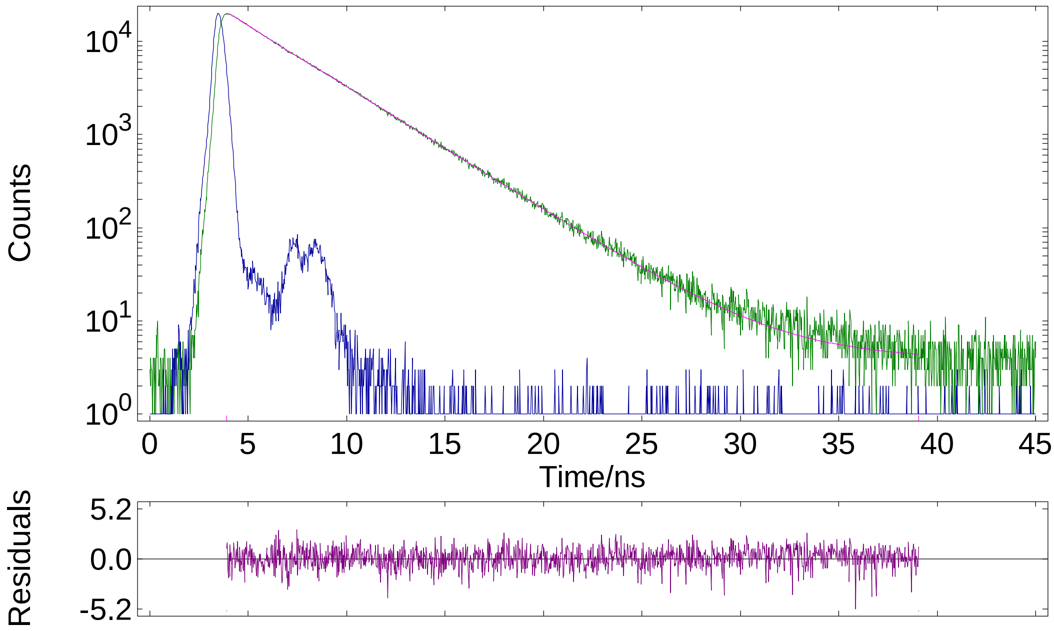

b

Figure S33. Time-resolved fluorescence lifetime decay profile of **5b** (a) in toluene, IRF (blue). λ_{ex} = 375 nm, λ_{em} = 445 nm; (b) in MeCN, IRF (blue). λ_{ex} = 375 nm, λ_{em} = 535 nm.

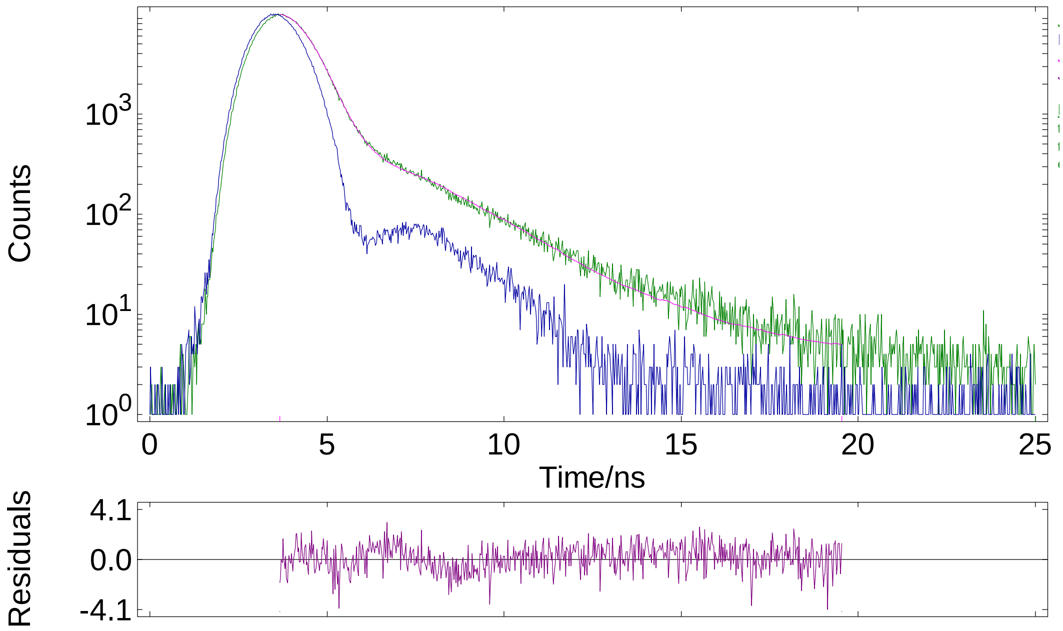

a

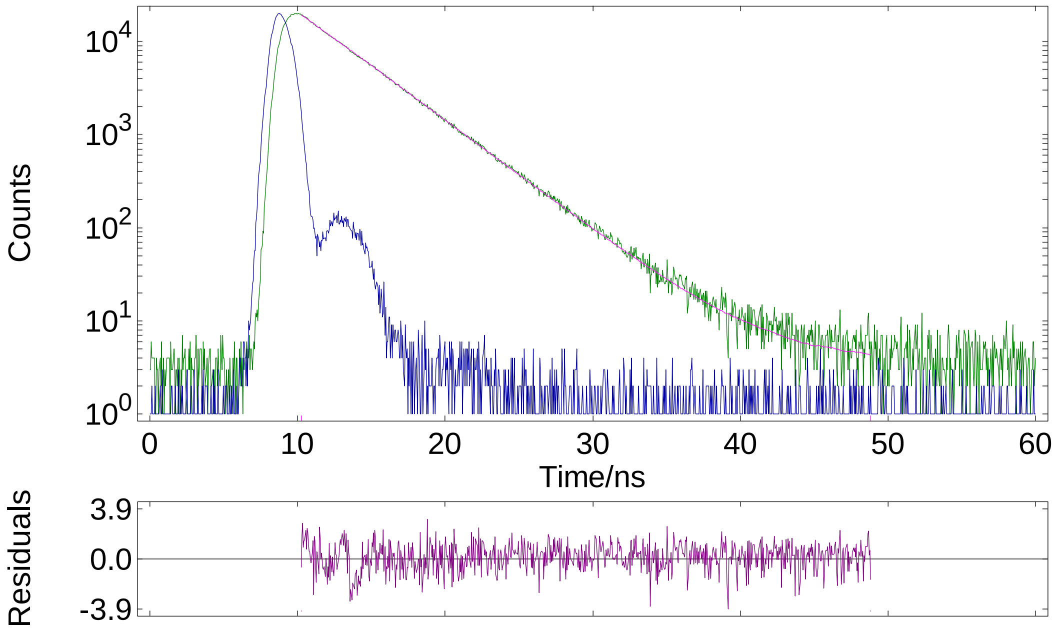

b

Figure S34. Time-resolved fluorescence lifetime decay profile of **5c** (a) in toluene, IRF (blue). λ_{ex} = 300 nm, λ_{em} = 415 nm; (b) in MeCN, IRF (blue). λ_{ex} = 300 nm, λ_{em} = 490 nm.

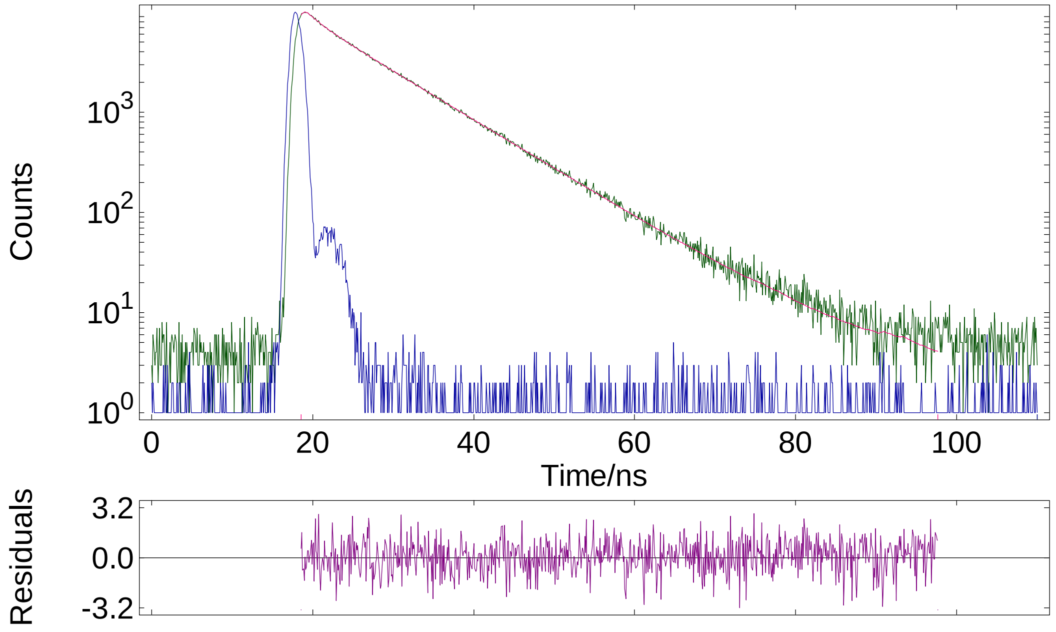

Figure S35. Time-resolved fluorescence lifetime decay profile of **6a** in toluene, IRF (blue). λ_{ex} = 300 nm, λ_{em} = 460 nm.

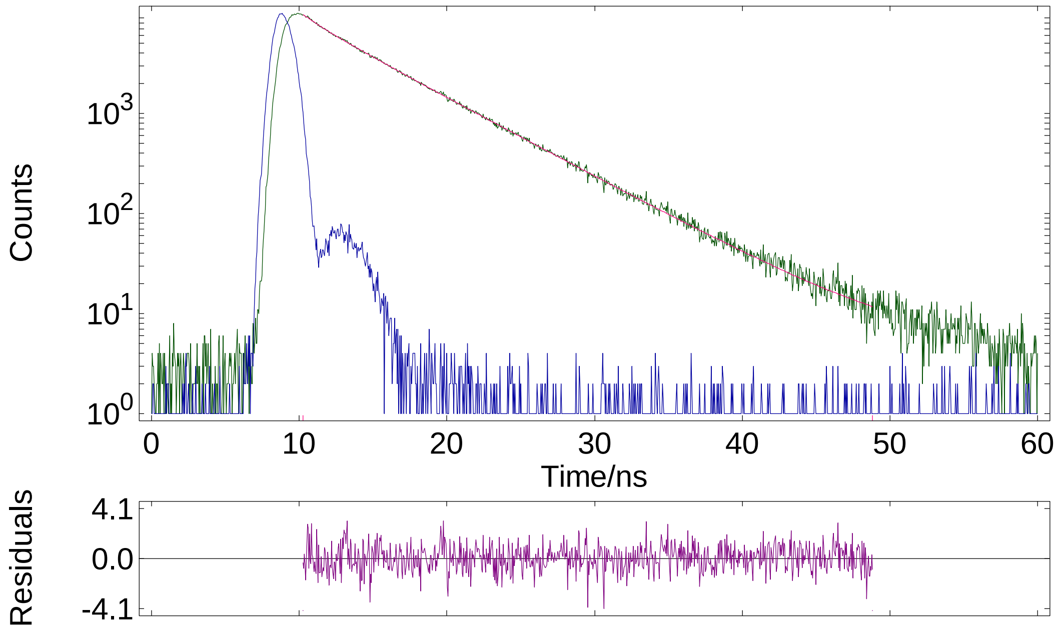

a

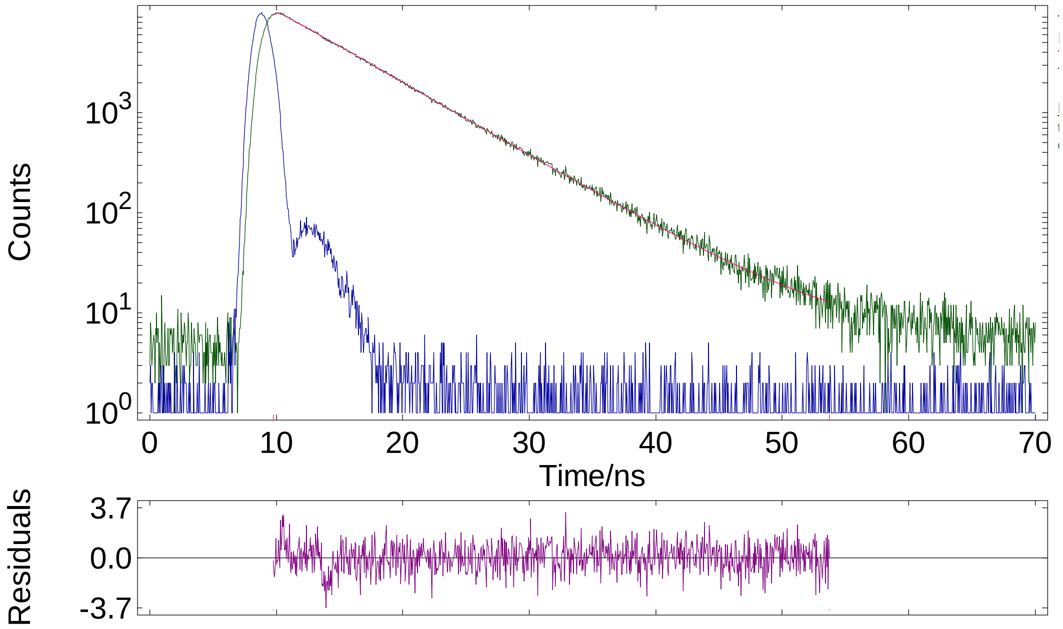

b

Figure S36. Time-resolved fluorescence lifetime decay profile of **6b** (a) in toluene, IRF (blue). λ_{ex} = 300 nm, λ_{em} = 430 nm; (b) in MeCN, IRF (blue). λ_{ex} = 300 nm, λ_{em} = 565 nm.

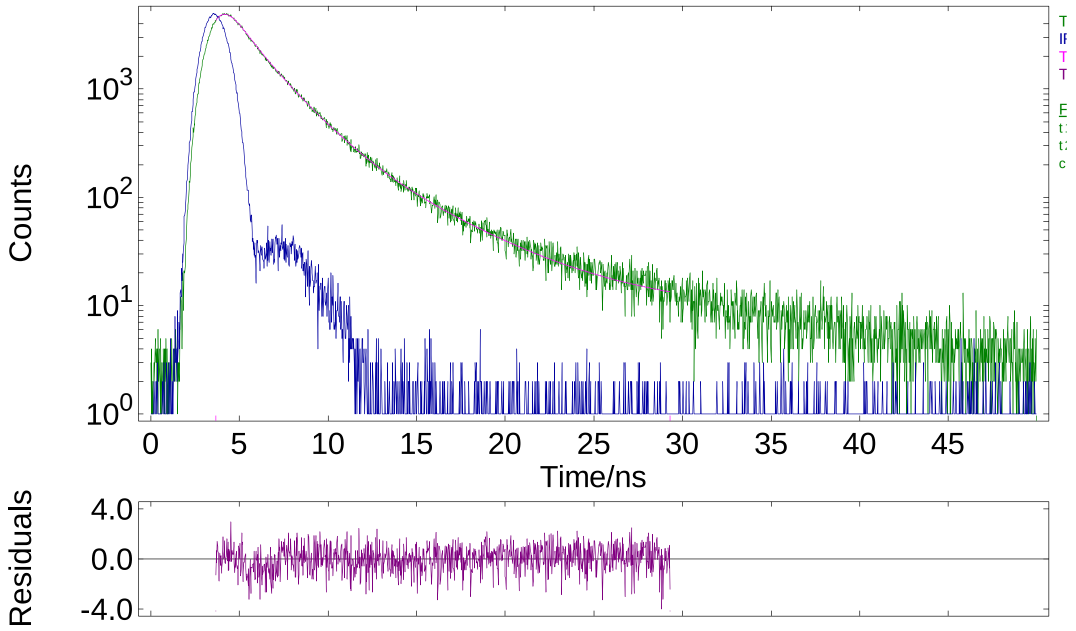

a

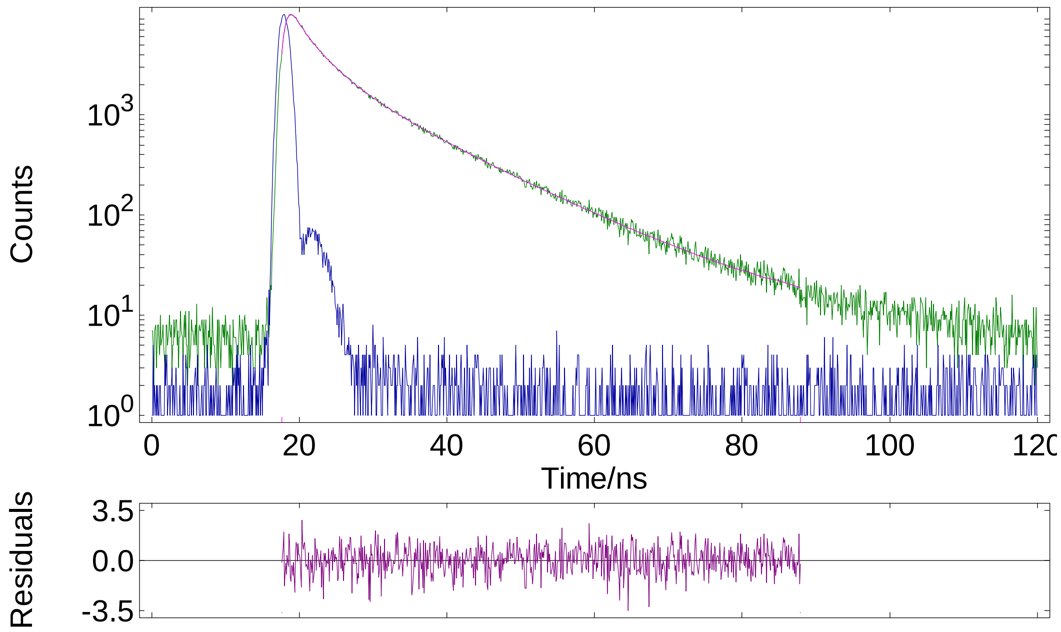

b

Figure S37. Time-resolved fluorescence lifetime decay profile of **6c** (a) in toluene, IRF (blue). λ_{ex} = 300 nm, λ_{em} = 430 nm; (b) in MeCN, IRF (blue). λ_{ex} = 300 nm, λ_{em} = 408 nm.

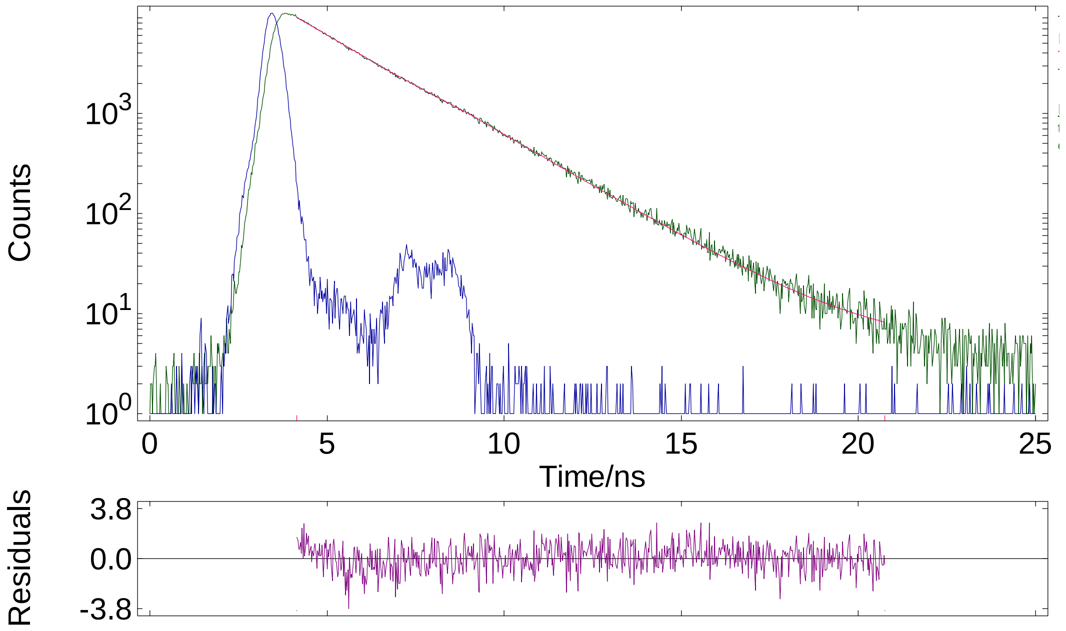

a

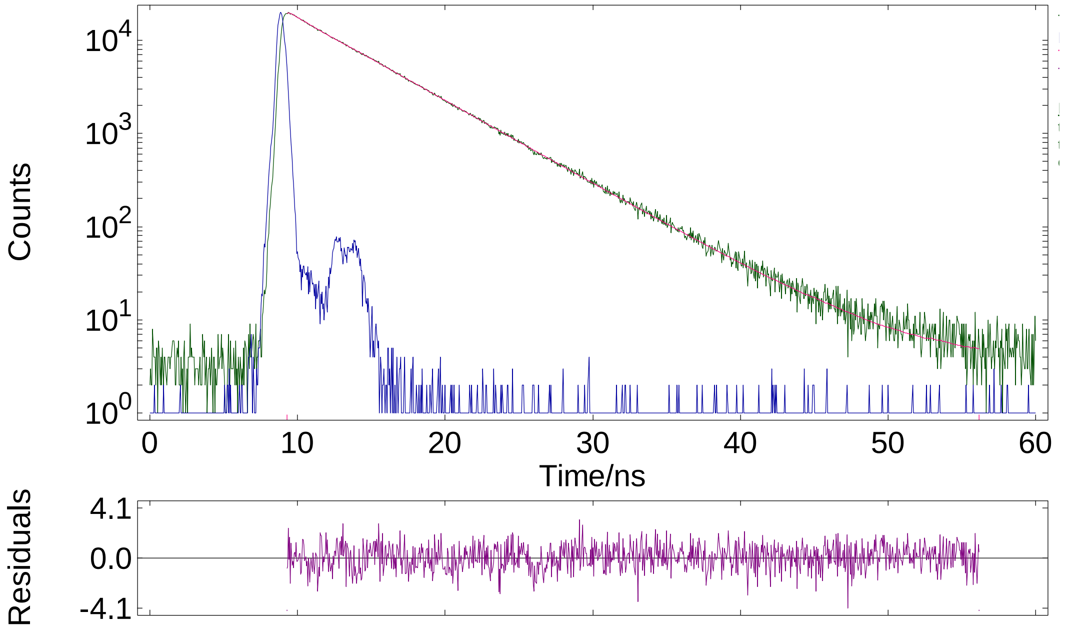

b

Figure S38. Time-resolved fluorescence lifetime decay profile of **10** (a) in toluene, IRF (blue). λ_{ex} = 375 nm, λ_{em} = 430 nm; (b) in MeCN, IRF (blue). λ_{ex} = 375 nm, λ_{em} = 500 nm.

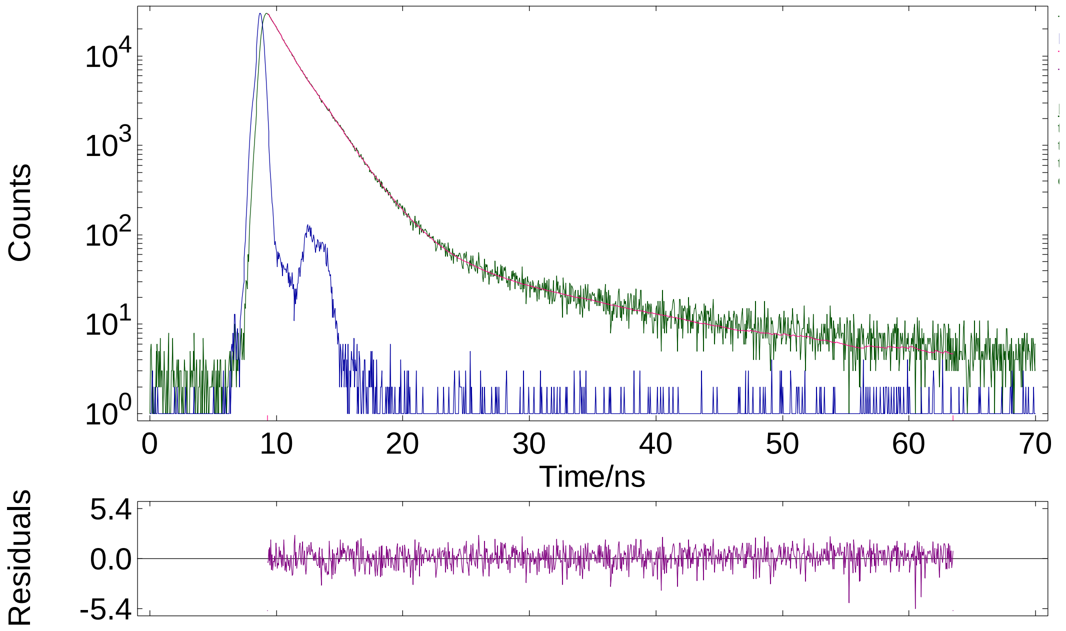

a

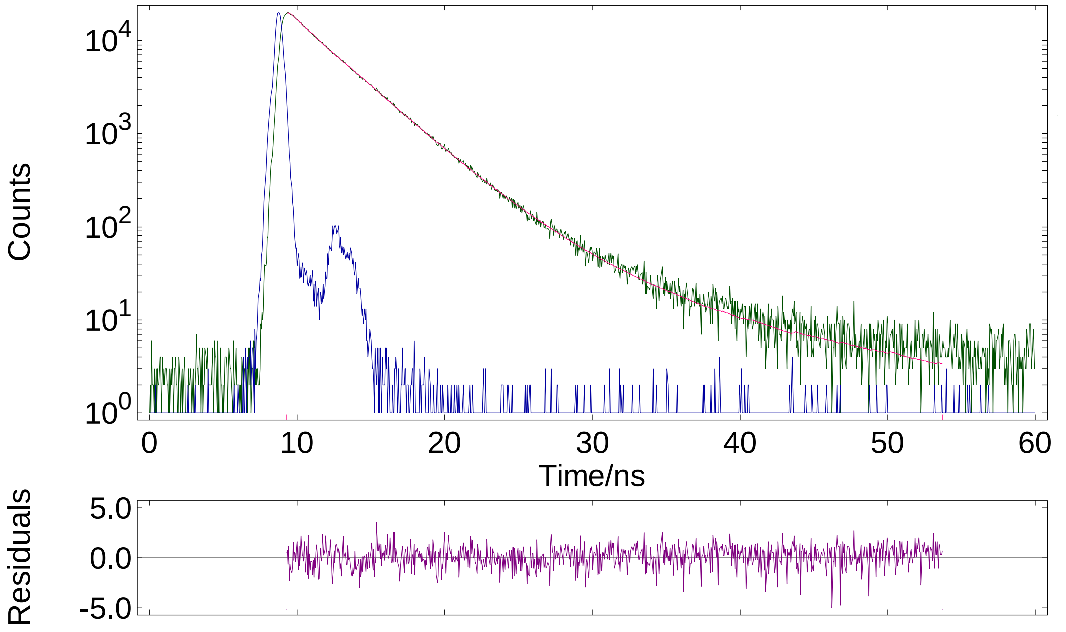

b

Figure S39. Time-resolved fluorescence lifetime decay profile of **7a** (a) in toluene, IRF (blue). λ_{ex} = 375 nm, λ_{em} = 471 nm; (b) in MeCN, IRF (blue). λ_{ex} = 375 nm, λ_{em} = 539 nm.

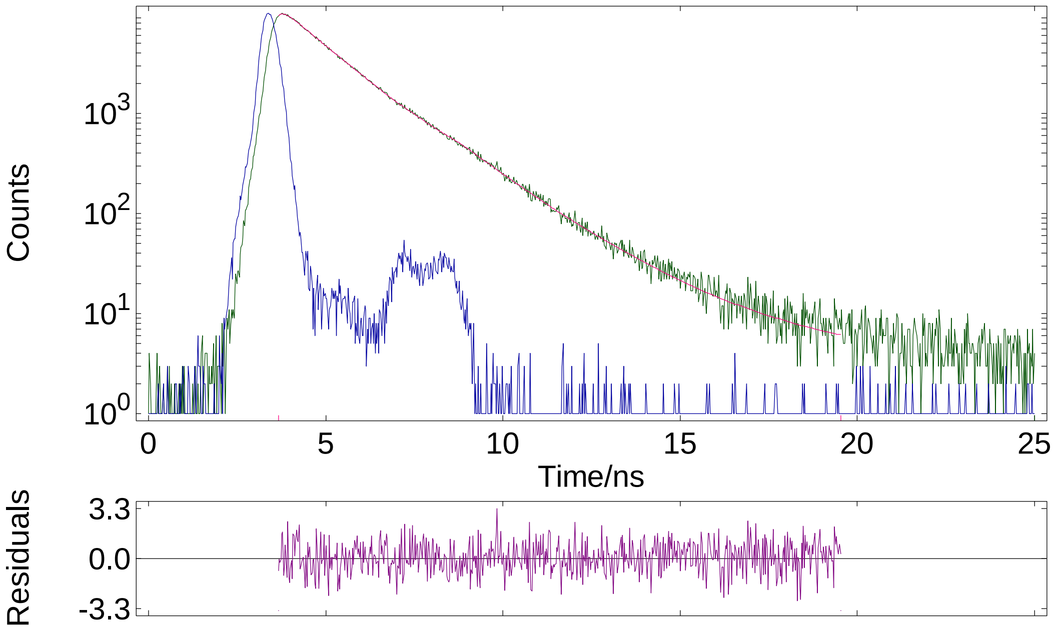
Counts
10^3
10^2
10^1
10^0
0
5
10
15
20
25
Time/ns
Residuals
3.3
0.0
-3.3

a

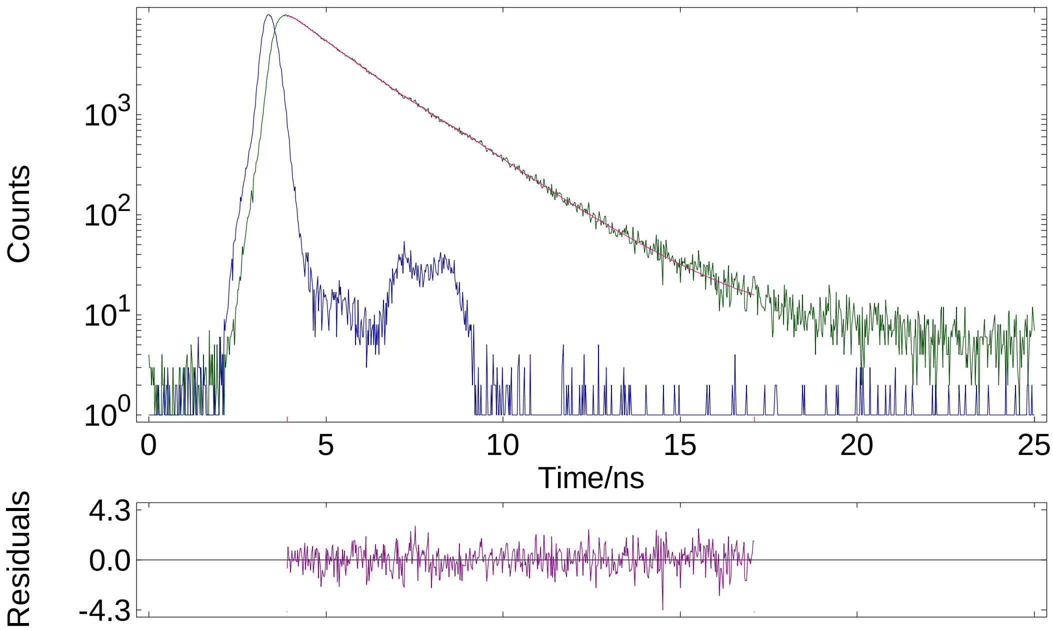
Counts
10^3
10^2
10^1
10^0
0
5
10
15
20
25
Time/ns
Residuals
4.3
0.0
-4.3

b

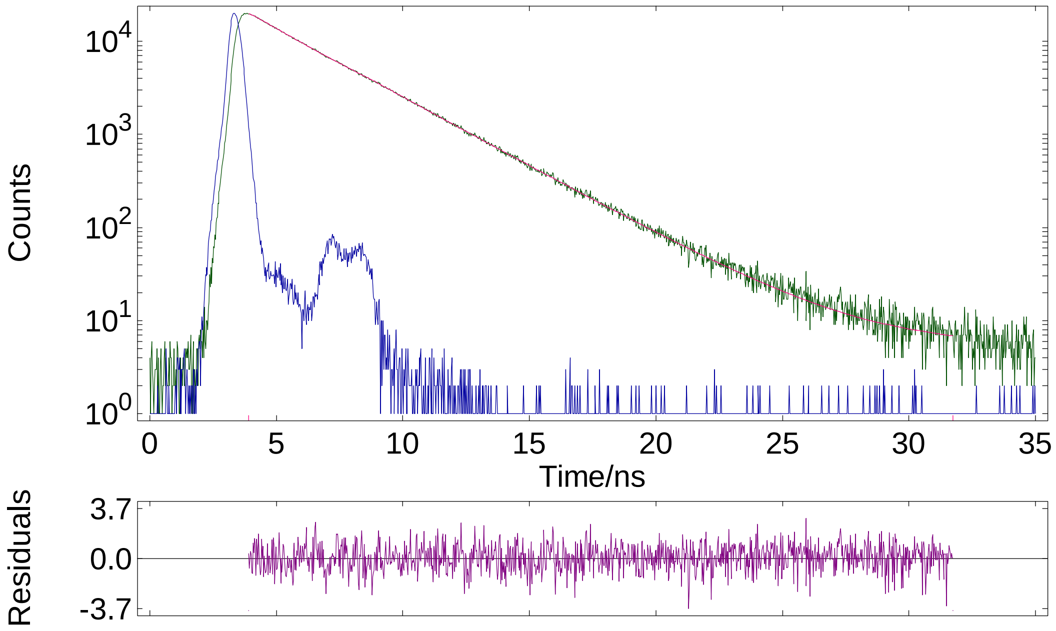

c

Figure S40. Time-resolved fluorescence lifetime decay profile of **7b** (a) in toluene, IRF (blue). λ_{ex} = 375 nm, λ_{em} = 480 nm; (b) in toluene, IRF (blue). λ_{ex} = 375 nm, λ_{em} = 621 nm; (c) in MeCN, IRF (blue). λ_{ex} = 375 nm, λ_{em} = 525 nm.

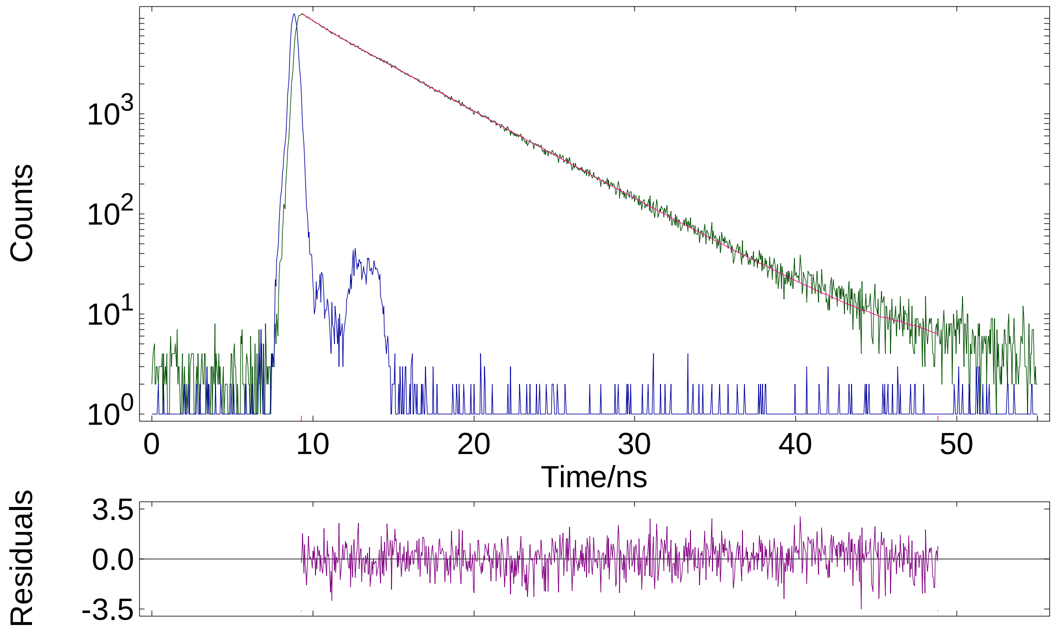

a

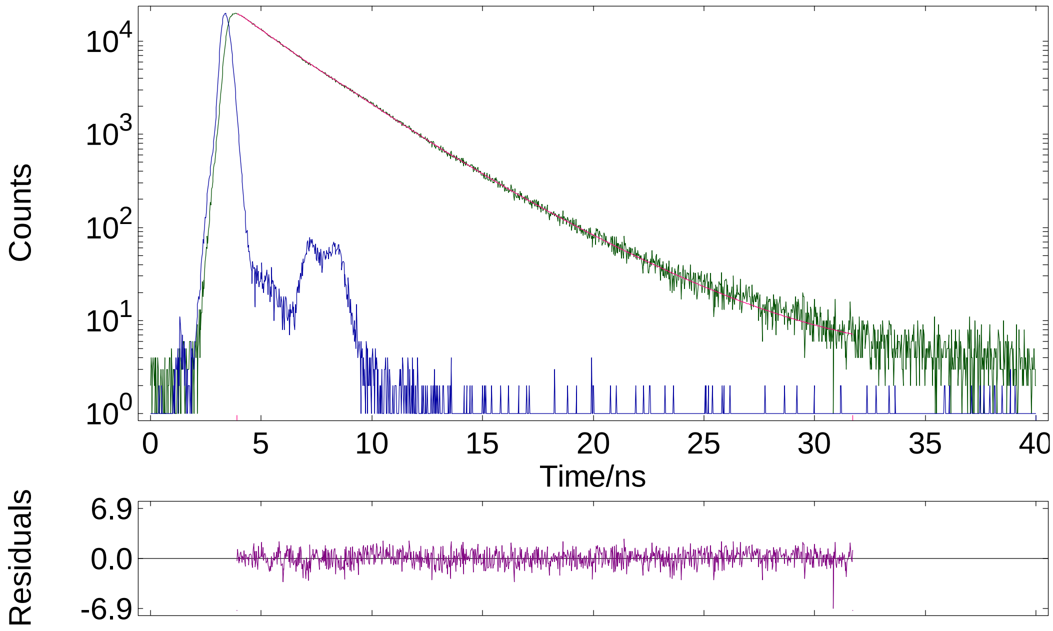

b

Figure S41. Time-resolved fluorescence lifetime decay profile of **7c** (a) in toluene, IRF (blue). λ_{ex} = 375 nm, λ_{em} = 541 nm; (b) in MeCN, IRF (blue). λ_{ex} = 375 nm, λ_{em} = 491 nm.

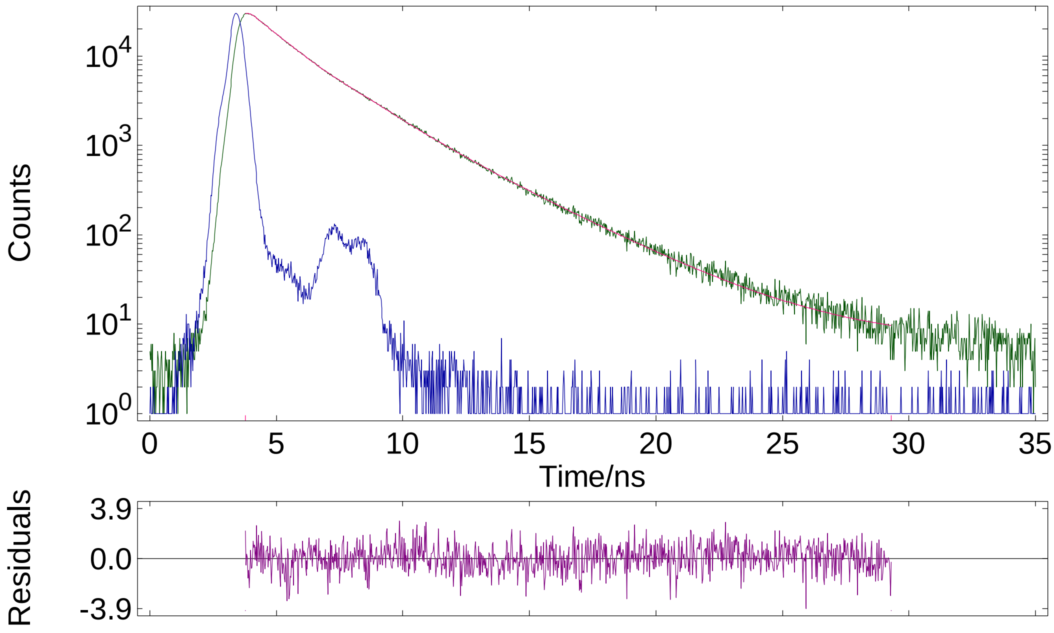

a

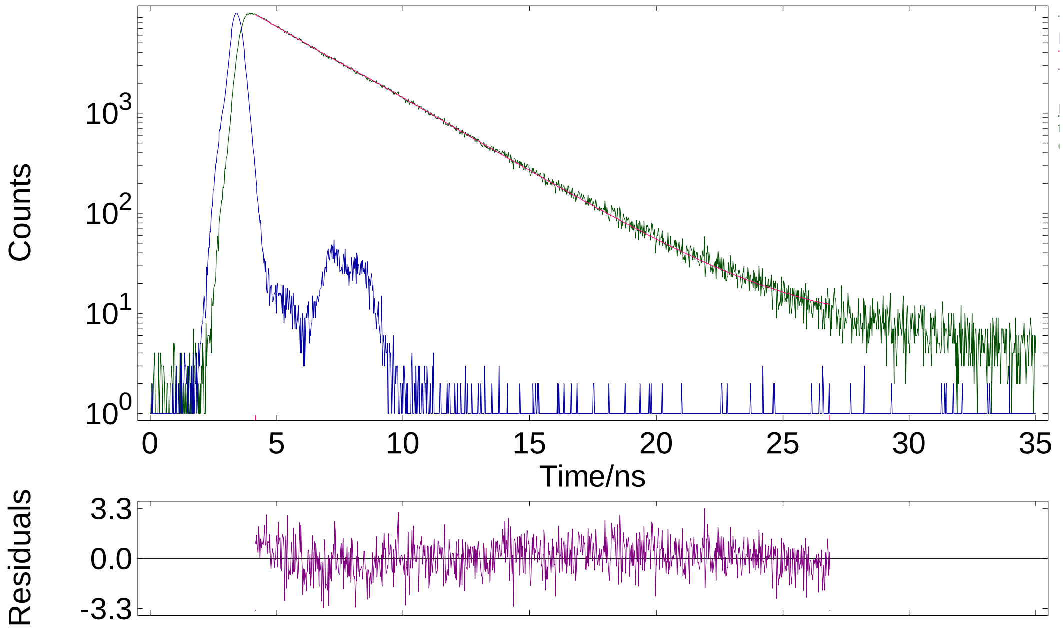

b

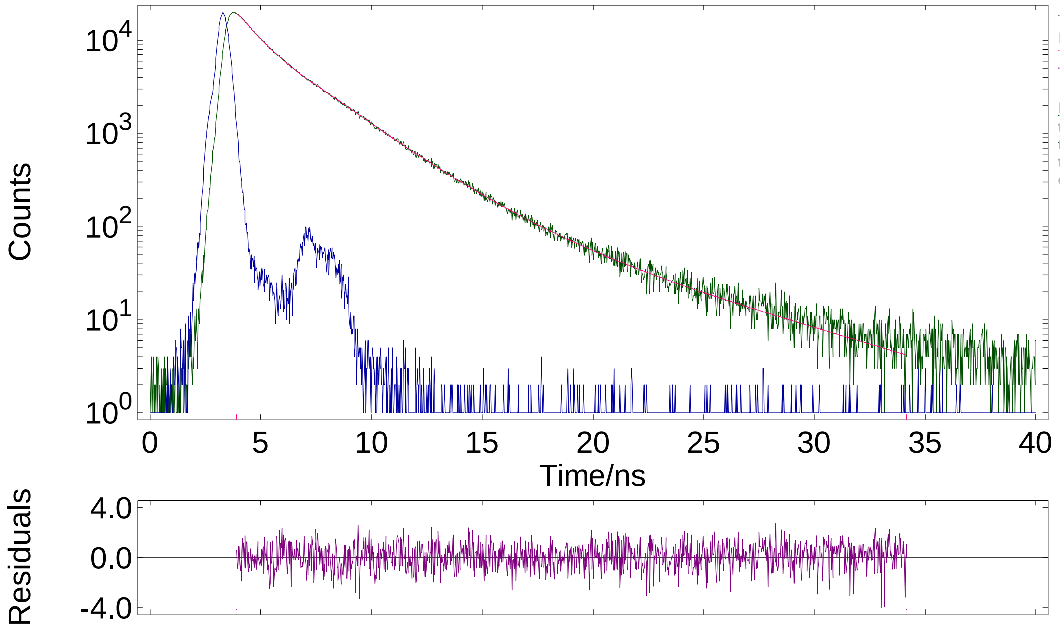

c

Figure S42. Time-resolved fluorescence lifetime decay profile of **8a** (a) in toluene, IRF (blue). λ_{ex} = 375 nm, λ_{em} = 460 nm; (b) in toluene, IRF (blue). λ_{ex} = 375 nm, λ_{em} = 600 nm; (c) in MeCN, IRF (blue). λ_{ex} = 375 nm, λ_{em} = 530 nm.

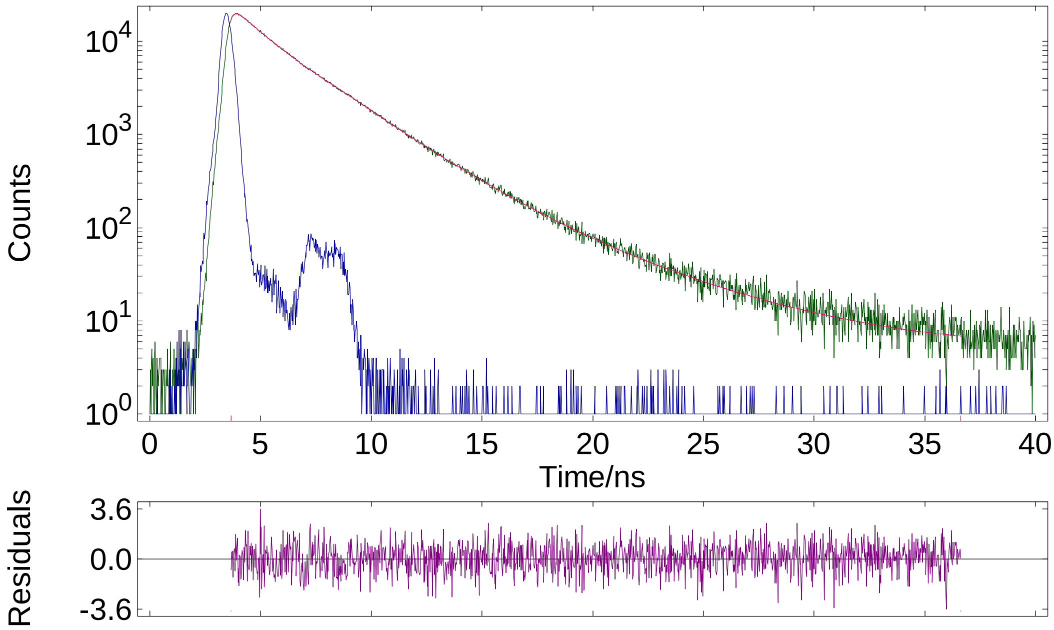

a

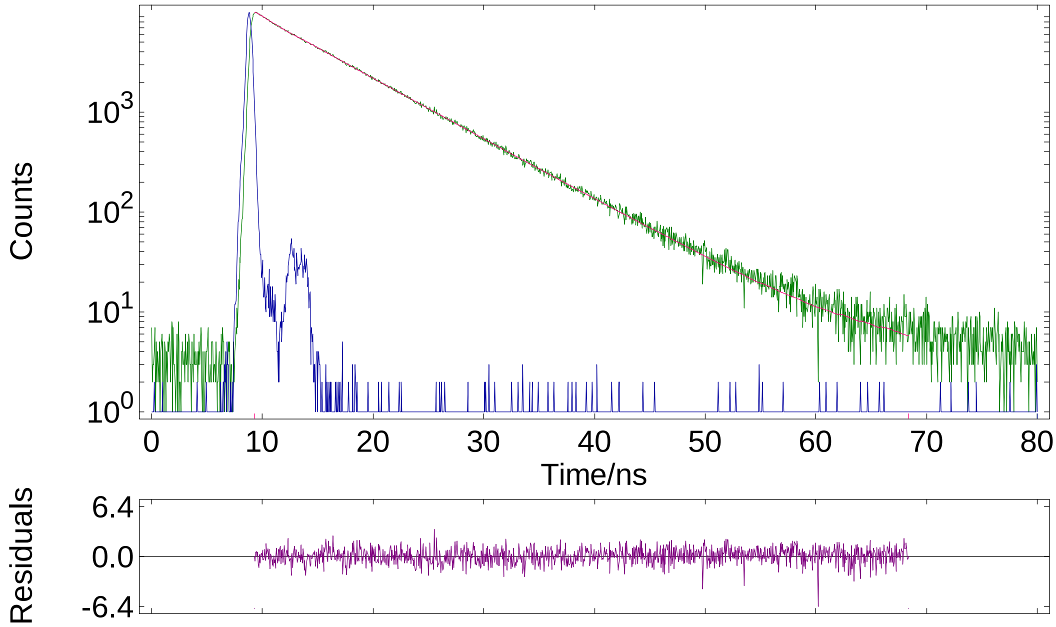

b

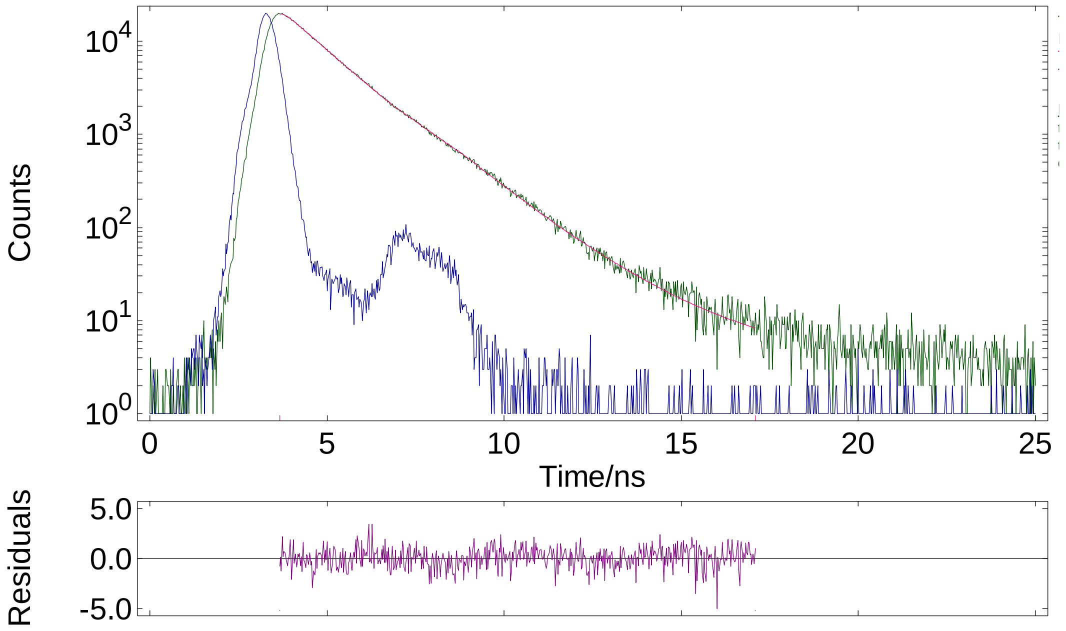

c

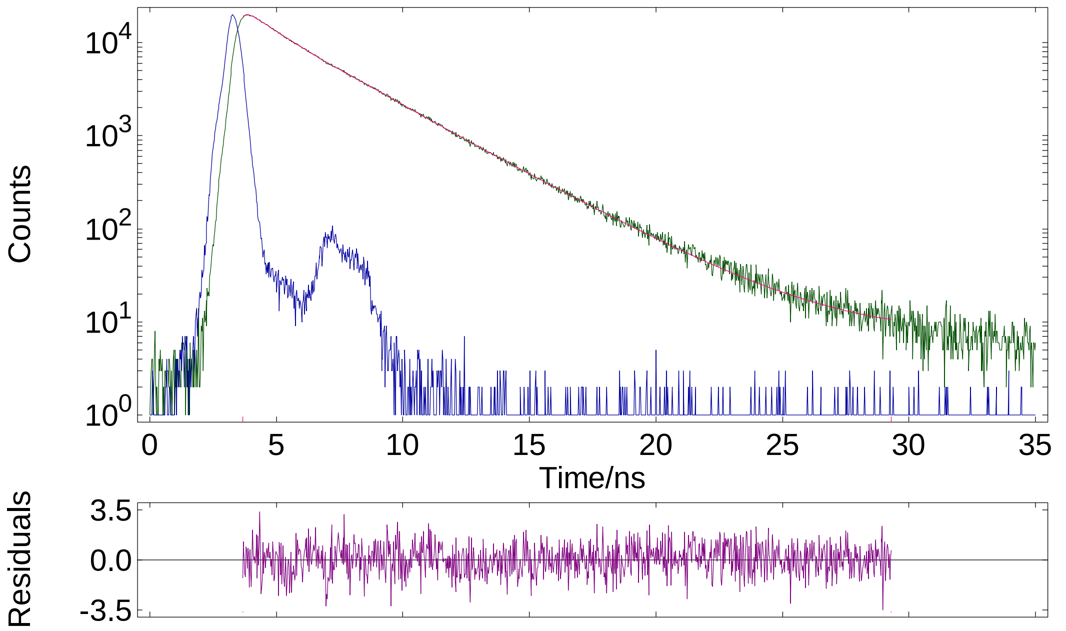

d

Figure S43. Time-resolved fluorescence lifetime decay profile of **8b** (a) in toluene, IRF (blue). λ_{ex} = 375 nm, λ_{em} = 465 nm; (b) in toluene, IRF (blue). λ_{ex} = 375 nm, λ_{em} = 555 nm; (c) in MeCN, IRF (blue). λ_{ex} = 375 nm, λ_{em} = 400 nm; (d) in MeCN, IRF (blue). λ_{ex} = 375 nm, λ_{em} = 540 nm.

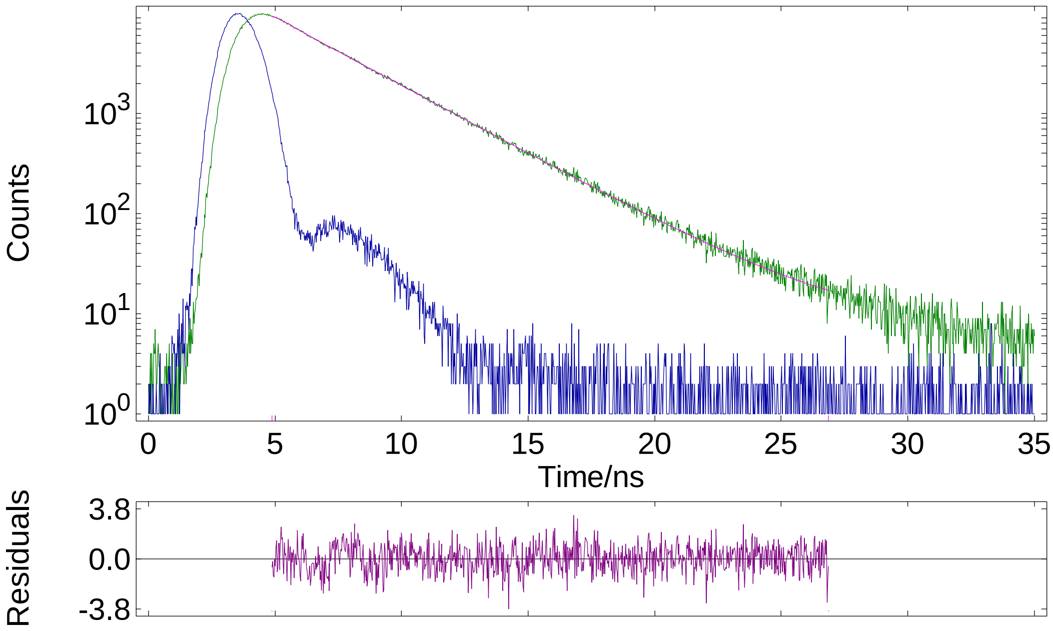

a

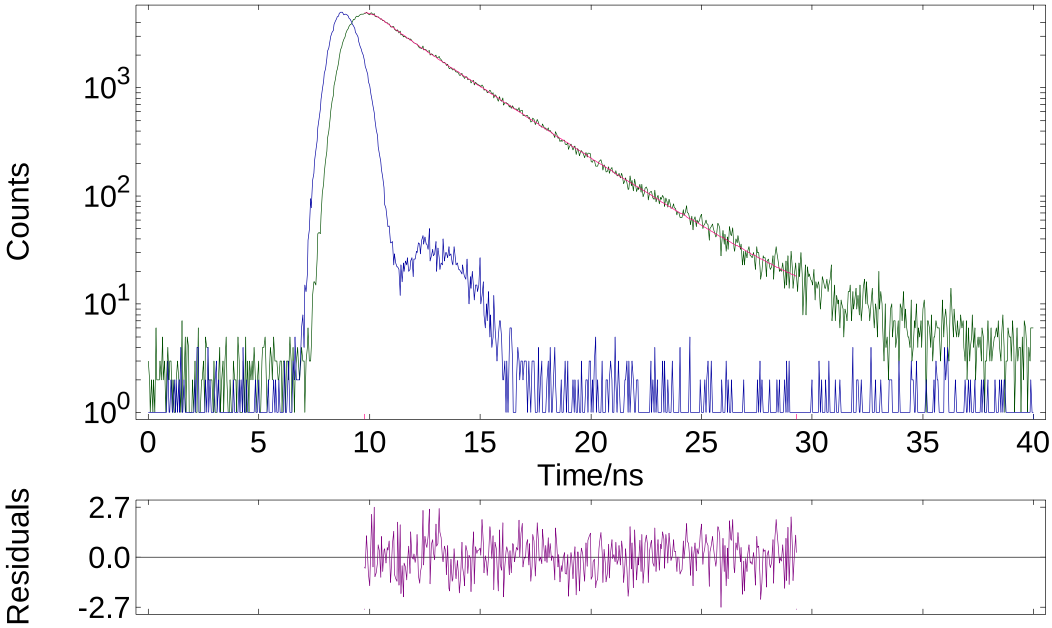

b

Figure S44. Time-resolved fluorescence lifetime decay profile of **9** (a) in toluene, IRF (blue). λ_{ex} = 300 nm, λ_{em} = 468 nm; (b) in MeCN, IRF (blue). λ_{ex} = 300 nm, λ_{em} = 560 nm.

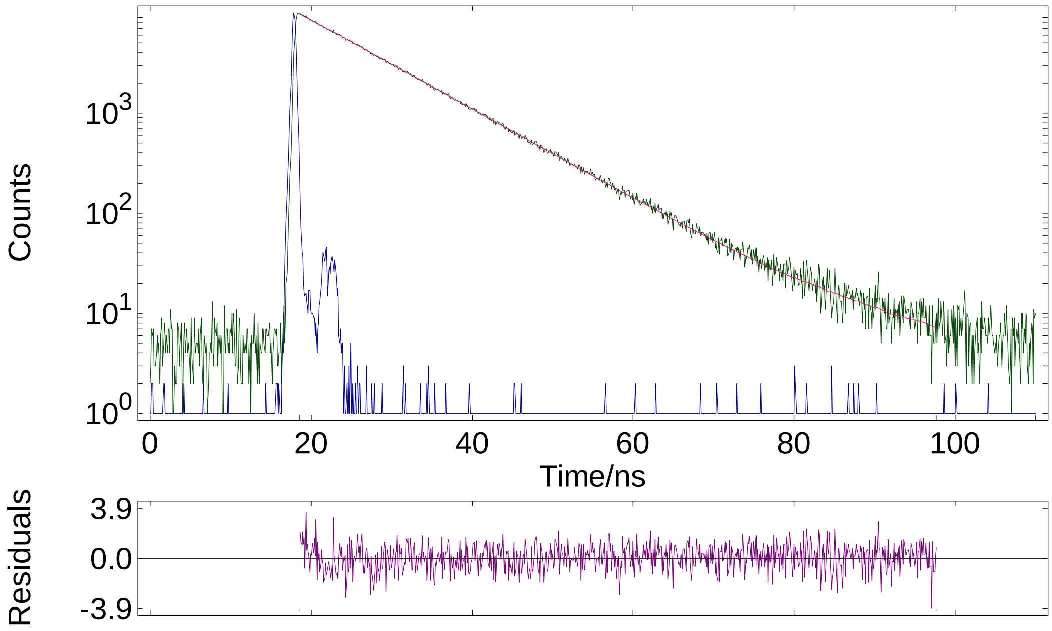

Figure S45. Time-resolved fluorescence lifetime decay profile of **11** in toluene, IRF (blue). λ_{ex} = 375 nm, λ_{em} = 570 nm.

6. Emission spectra of chromophores in solid state

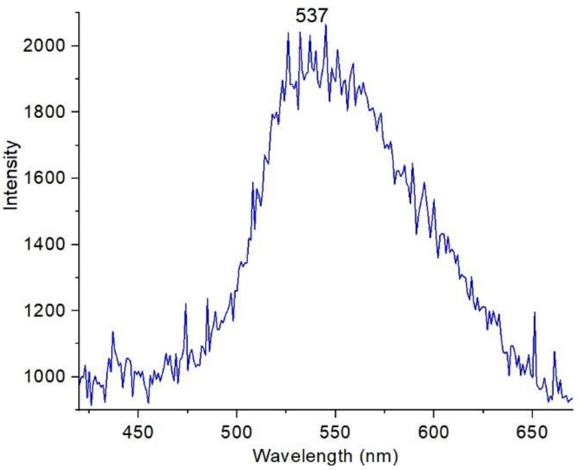

4a

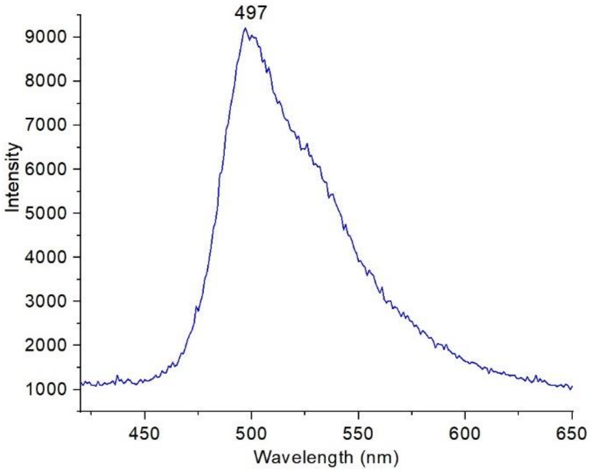

4b

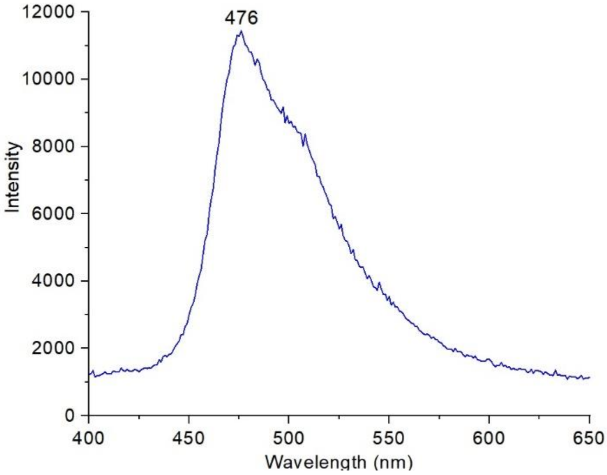

4c

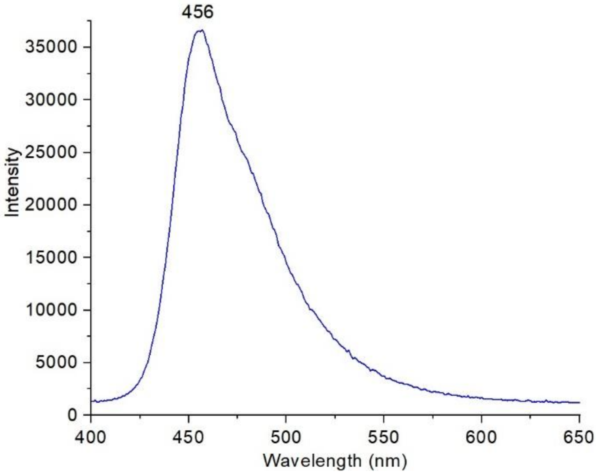

5a

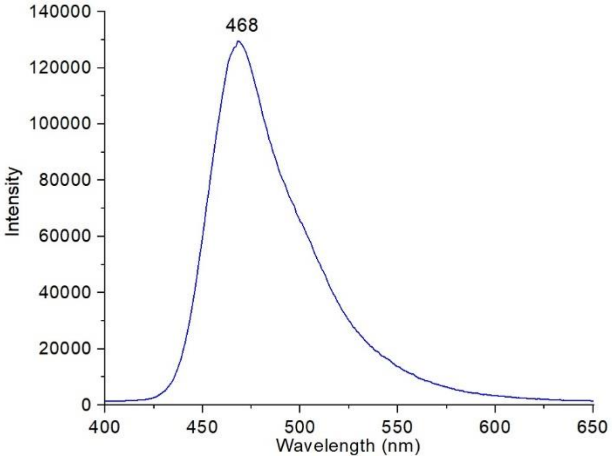

5b

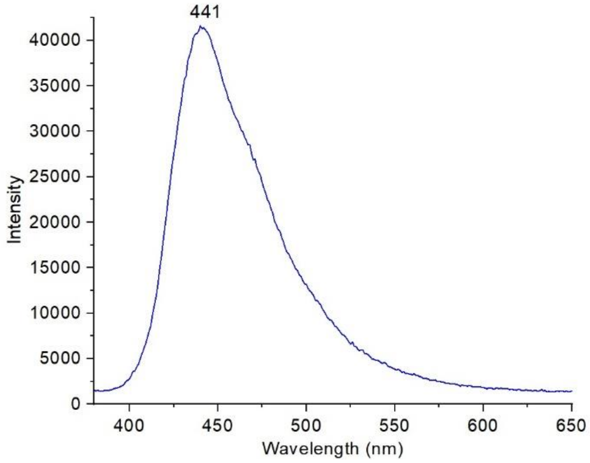

5c

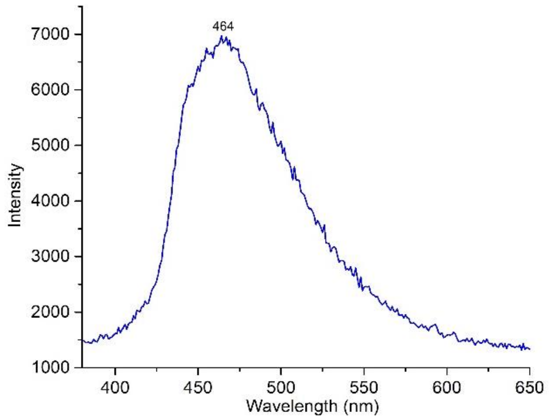

6a

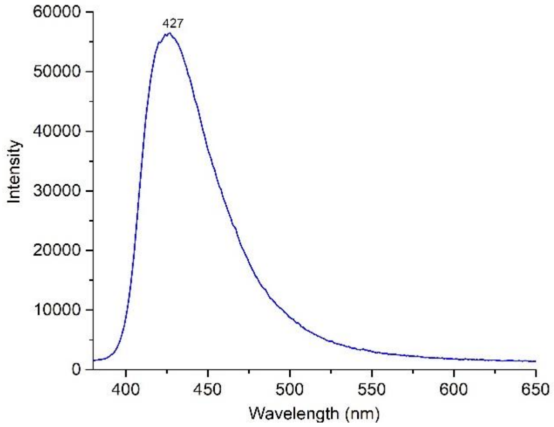

6b

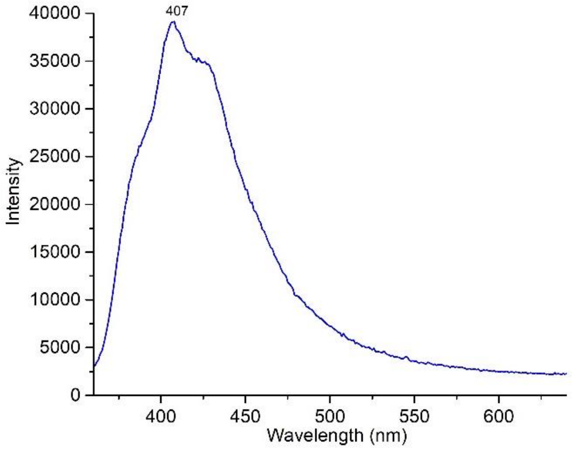

6c

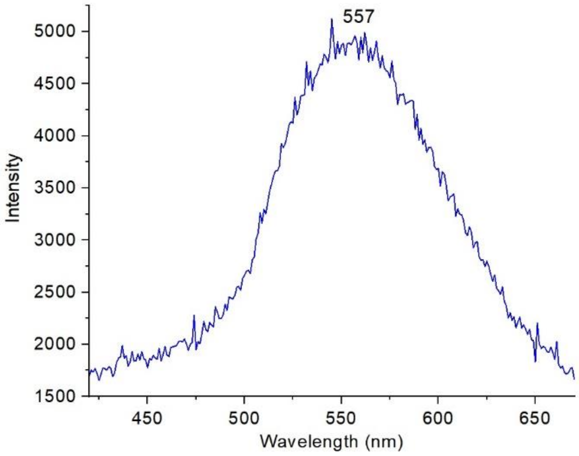

10

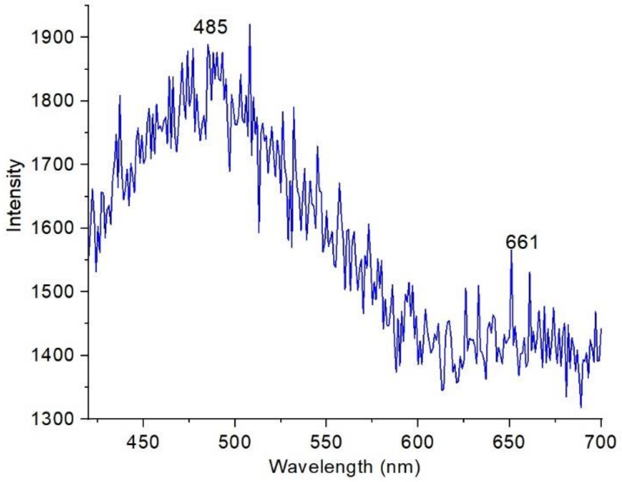

7a

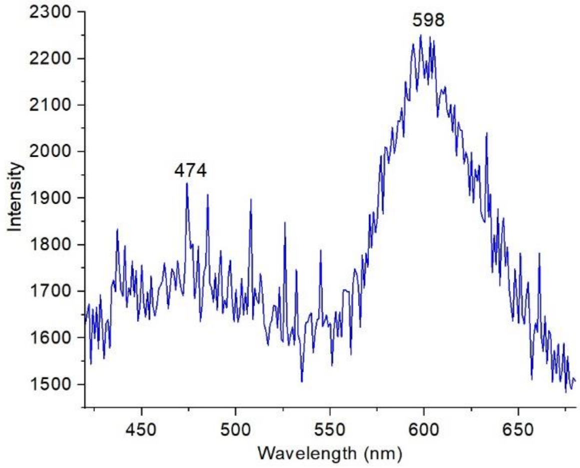

7b

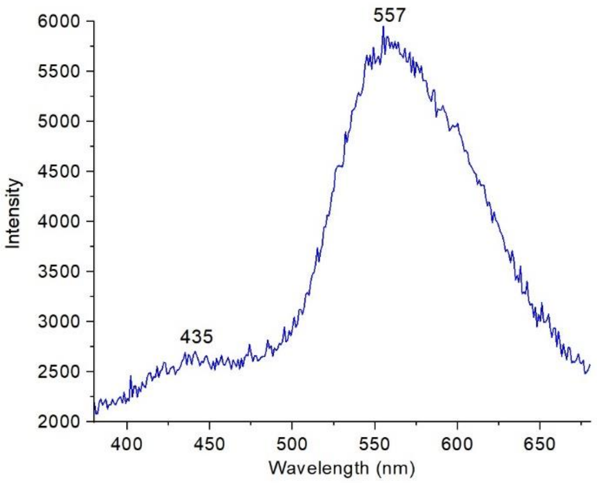

7c

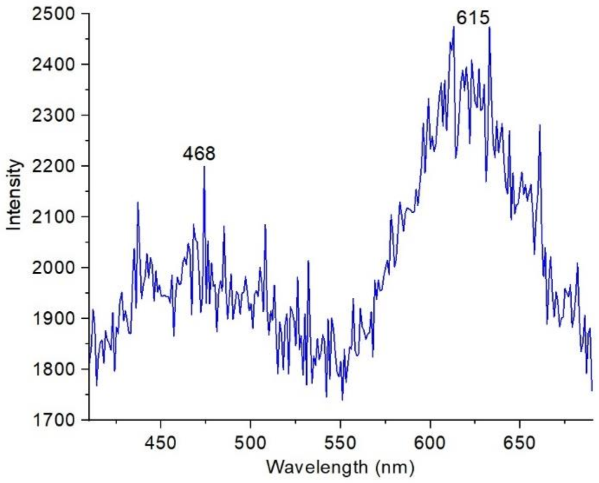

8a

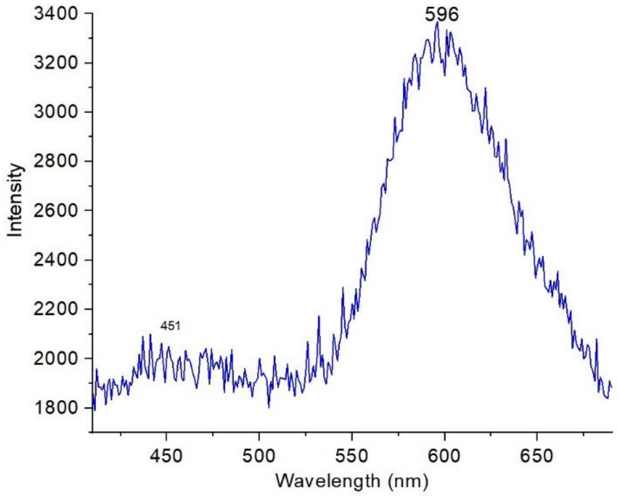

8b

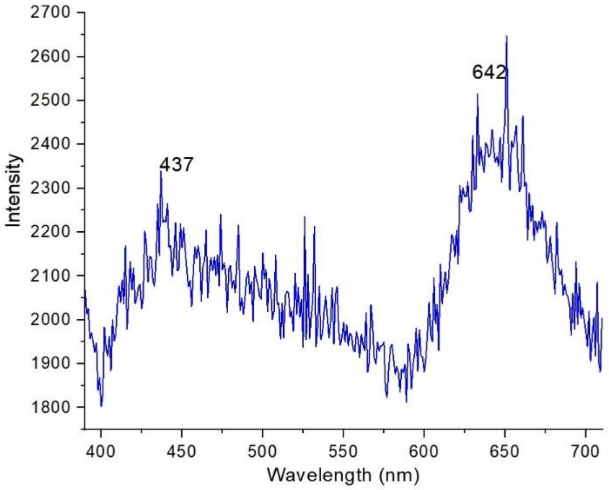

9

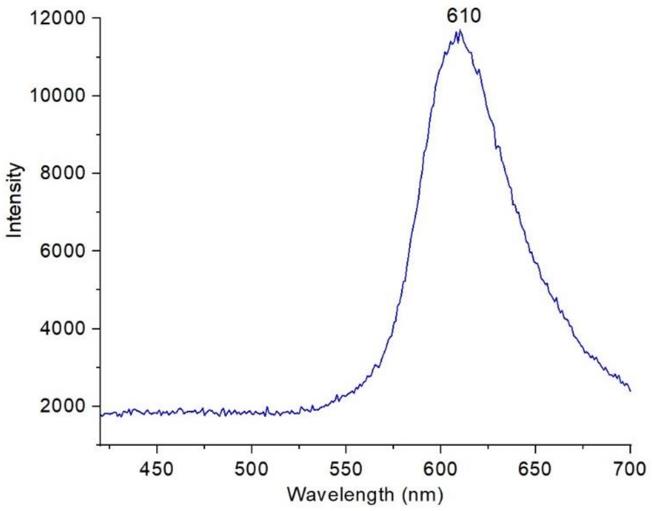

11

Figure S46. The emission spectra of compounds **4–11** in solid state.

Behavior of chromophores in MeCN/water mixture

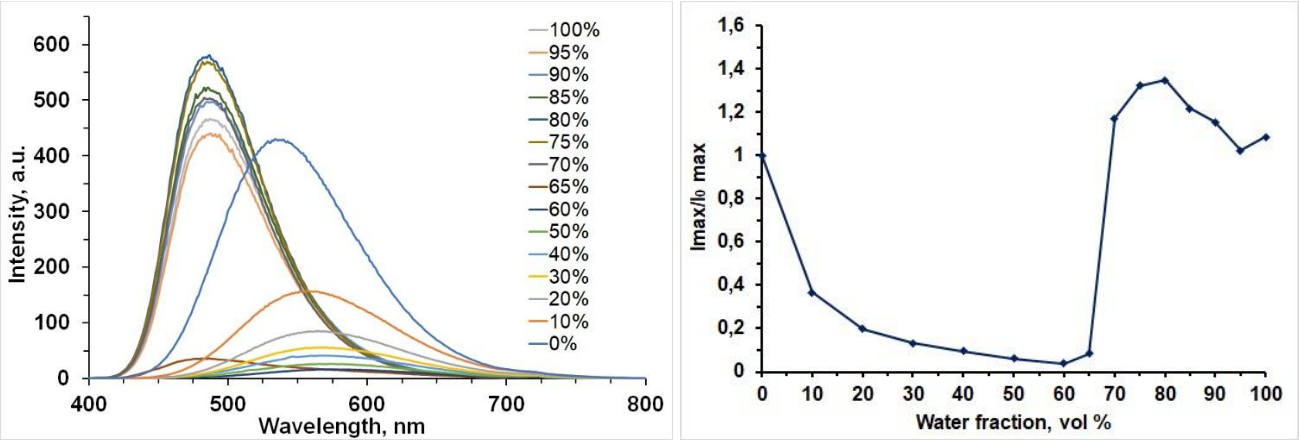

a b

Figure S47. (a) The fluorescence spectra of 10 mM **5b** in MeCN/H_2O mixtures with different water fractions (f_w). (b) A plot of I/I_0 versus the composition of the MeCN/H_2O mixture for **5b**.

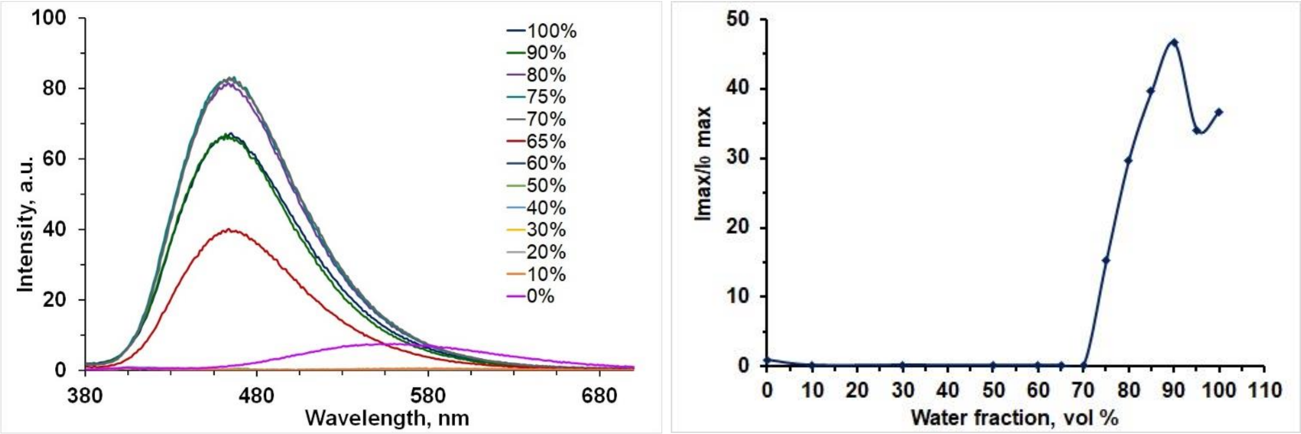

a b

Figure S48. (a) The fluorescence spectra of 10 mM **6b** in MeCN/H_2O mixtures with different water fractions (f_w). (b) A plot of I/I_0 versus the composition of the MeCN/H_2O mixture for **6b**.

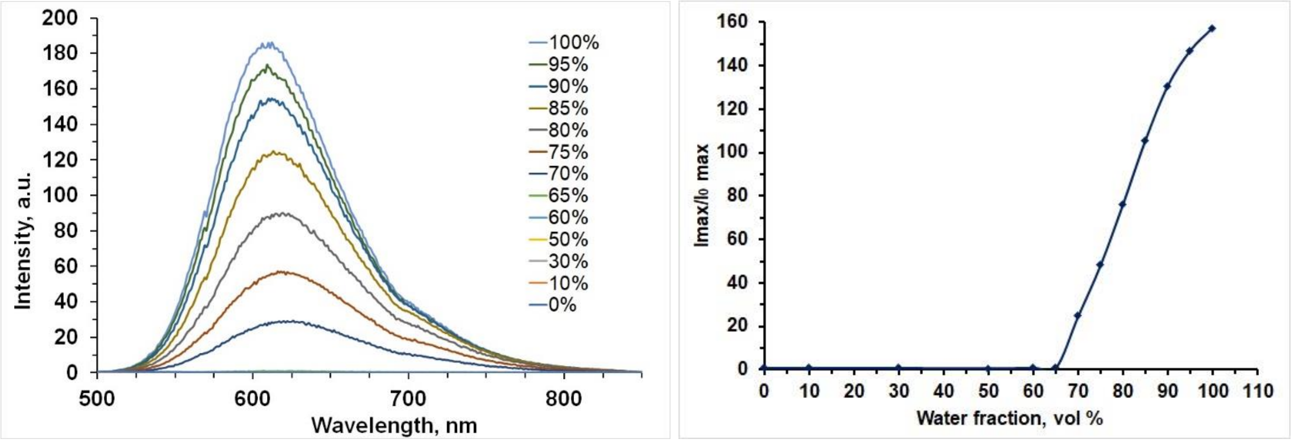

a b

Figure S49. (a) The fluorescence spectra of 10 mM **11** in MeCN/H_2O mixtures with different water fractions (f_w). (b) A plot of I/I_0 versus the composition of the MeCN/H_2O mixture for **11**.

7. Quantum-chemical calculations

Table S4. Calculated frontier molecular orbitals (HOMO, LUMO) of quinazolin-4(3*H*)-ones **4a-c**, **5a-c**, **6a-c**, **10** and 4-cyanoquinazolines **8a,b, 9, 11**.

**Comp.**	**HOMO**	**LUMO**	Energy band gap (ΔE)
**4a**	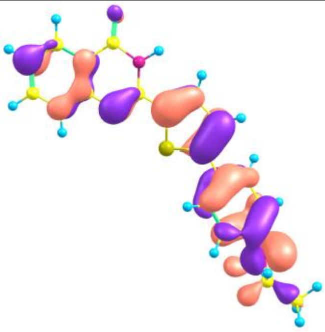 -5.6894	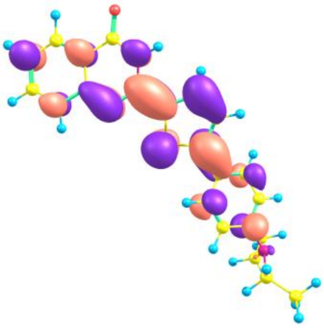 -2.0522	3.6372
**4b**	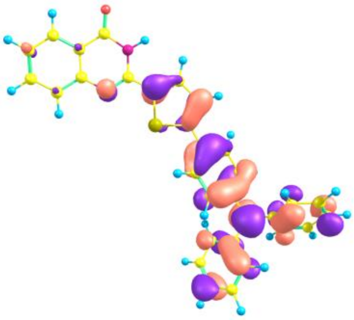 -5.2293	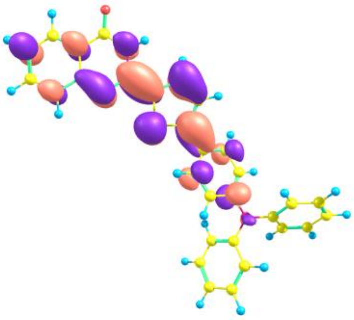 -2.0303	3.1990
**4c**	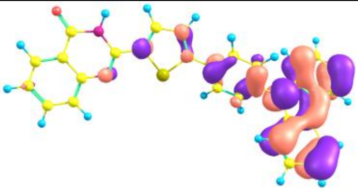 -5.5921	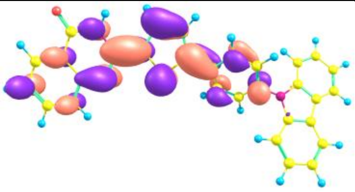 -2.2473	3.3448
**5a**	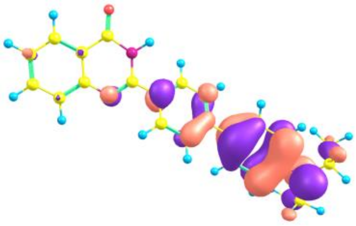 -5.2299	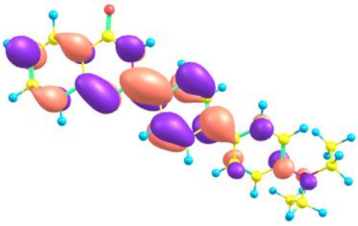 -1.6760	3.5539
**5b**	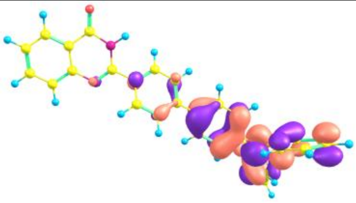 -5.2397	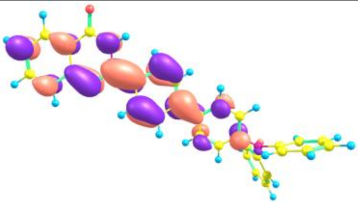 -1.8526	3.3871
**5c**	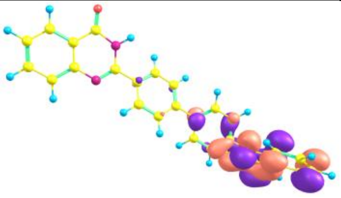 -5.5848	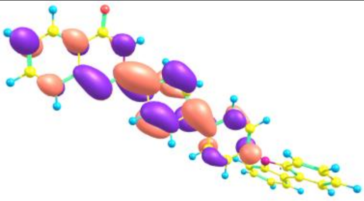 -2.0376	3.5472

**6a**	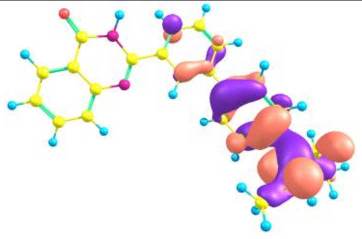 -5.8023	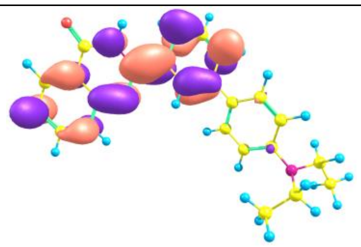 -1.7431	4.0592
**6b**	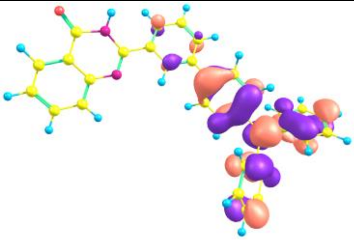 -5.1998	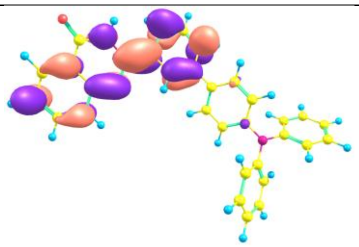 -1.7321	3.4677
**6c**	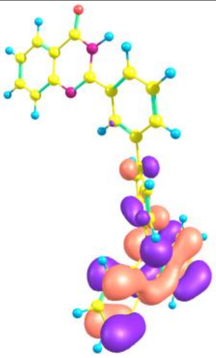 -5.5502	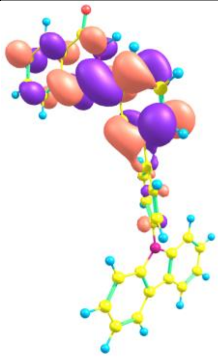 -1.8947	3.6555
**10**	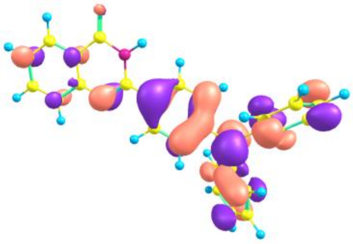 -5.3822	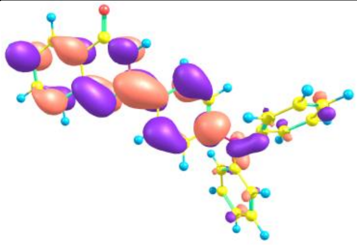 -1.6474	3.7348
**8a**	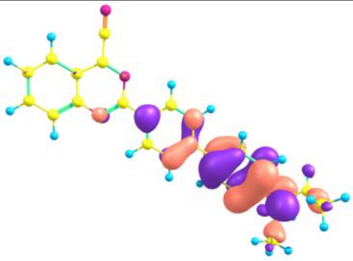 -5.1232	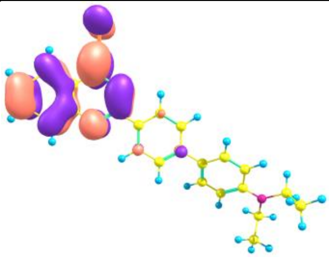 -2.6490	2.4742
**8b**	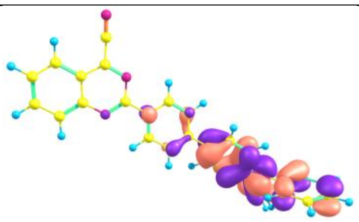 -5.1513	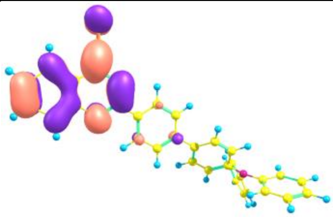 -2.7618	2.3895

**9**	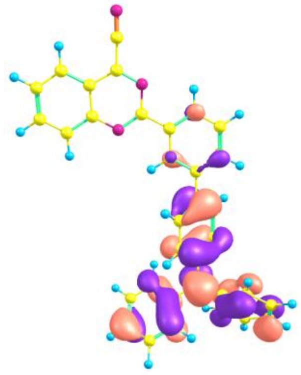 -5.1345	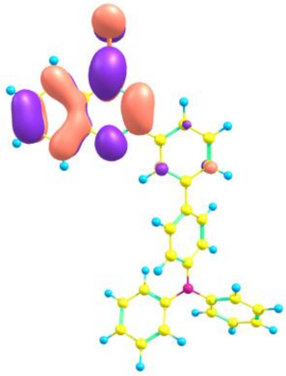 -2.8046	2.3299
**11**	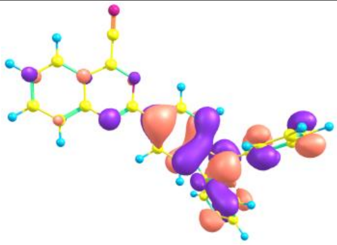 -5.2712	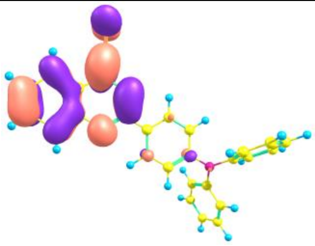 -2.6591	2.6121
